# Pharmacological blockage of transforming growth factor-β signalling by a Traf2- and Nck-interacting kinase inhibitor, NCB-0846

**DOI:** 10.1038/s41416-020-01162-3

**Published:** 2020-11-27

**Authors:** Teppei Sugano, Mari Masuda, Fumitaka Takeshita, Noriko Motoi, Toru Hirozane, Naoko Goto, Shigeki Kashimoto, Yuko Uno, Hideki Moriyama, Masaaki Sawa, Yuichi Nagakawa, Akihiko Tsuchida, Masahiro Seike, Akihiko Gemma, Tesshi Yamada

**Affiliations:** 1 Division of Chemotherapy and Clinical Research, National Cancer Center Research Institute, Tokyo, 104-0045 Japan; 2 Department of Pulmonary Medicine and Oncology, Graduate School of Medicine, Nippon Medical School, Tokyo, 113-8602 Japan; 3 Department of Functional Analysis, Fundamental Innovative Oncology Core Center (FIOC), National Cancer Center Research Institute, Tokyo, 104-0045 Japan; 4 Department of Diagnostic Pathology, National Cancer Center Hospital, Tokyo, 104-0045 Japan; 5 Department of Orthopedic Surgery, Keio University School of Medicine, Tokyo, 160-8582 Japan; 6 Carna Biosciences, Inc, Kobe, 650-0047 Japan; 7 Department of Gastrointestinal and Pediatric Surgery, Tokyo Medical University, Tokyo, 160-0023 Japan

**Keywords:** Target validation, Non-small-cell lung cancer

## Abstract

**Background:**

Metastasis is the primary cause of death in cancer patients, and its management is still a major challenge. Epithelial to mesenchymal transition (EMT) has been implicated in the process of cancer metastasis, and its pharmacological interference holds therapeutic promise.

**Methods:**

Traf2- and Nck-interacting kinase (TNIK) functions as a transcriptional coregulator of Wnt target genes. Given the convergence of Wnt and transforming growth factor-β (TGFβ) signalling, we examined the effects of a small-molecule TNIK inhibitor (named NCB-0846) on the TGFβ1-induced EMT of lung cancer cells.

**Results:**

NCB-0846 inhibited the TGFβ1-induced EMT of A549 cells. This inhibition was associated with inhibition of Sma- and Mad-Related Protein-2/3 (SMAD2/3) phosphorylation and nuclear translocation. NCB-0846 abolished the lung metastasis of TGFβ1-treated A549 cells injected into the tail veins of immunodeficient mice. The inhibition of EMT was mediated by suppression of the TGFβ receptor type-I (*TGFBR1*) gene, at least partly through the induction of microRNAs targeting the *TGFBR1* transcript [miR-320 (a, b and d) and miR-186].

**Conclusions:**

NCB-0846 pharmacologically blocks the TGFβ/SMAD signalling and EMT induction of lung cancer cells by transcriptionally downregulating *TGFBRI* expression, representing a potentially promising approach for prevention of metastasis in lung cancer patients.

## Background

Metastasis is the primary cause of death in cancer patients, and its management is still a major challenge in clinical oncology. EMT was originally described in the context of embryonic development, but is now known to play a central role in cancer metastasis.^1–3^ During the process of EMT, cancer cells lose epithelial cell adhesion/polarity and acquire a mesenchymal phenotype. Adherens junctions are disrupted, and their components such as E-cadherin, β-catenin, p120-catenin and α-catenin are often downregulated and/or dissociated, whereas mesenchymal cell markers including N-cadherin, vimentin and fibronectin are upregulated.^2^ EMT increases cell plasticity and motility, thereby permitting tumour cells to leave their original sites, enter the systemic circulation, and reconstruct tumours at distant sites.^4^ The development of therapeutic measures to inhibit EMT would therefore be of paramount importance for prevention of cancer metastasis.

The canonical TGFβ/SMAD signalling pathway is known to mediate EMT through the transactivation of TGFβ-responsive transcription factors such as the Snail/Slug, Twist and zinc finger E-box-binding homeobox (ZEB) families,^1^ but involvement of canonical Wnt/β-catenin signalling in the TGFβ-induced EMT is also evident.^1,5^ Disruption of the adherens junction liberates β-catenin.^6^ Snail^7^ and Twist^8^ are known Wnt target genes (https://web.stanford.edu/group/nusselab/cgi-bin/wnt/target_genes). LEF (lymphoid enhancer factor)/TCF (T-cell factor) transcription factors physically associate with SMADs, and TGFβ-dependent Wnt target gene expression requires both SMAD and LEF/TCF DNA-binding motifs.^9^ ICG-001, a small-molecule inhibitor of β-catenin and cyclic AMP response element–binding protein (CREB)-binding protein (CBP) interaction, has been shown to suppress TGFβ-induced EMT.^10^

TNIK is a component of the β-catenin/TCF4 transcriptional complex and is essential for the transcription of Wnt target genes.^11,12^ NCB-0005 (also renamed KY-05009) is a TNIK-inhibiting small-molecule compound that we developed previously.^13^ NCB-0005 reportedly suppresses the TGFβ1-induced activation of Wnt signalling and inhibits the nuclear translocation of β-catenin, resulting in reduction of EMT marker (N-cadherin and vimentin) expression.^14^ TNIK phosphorylates the conserved serine 154 residue of TCF4.^12^ TGFβ1 induces the phosphorylation of TCF4, and the induction is inhibited by NCB-0005.^14^ Kaneko et al. identified TNIK as one of the kinases responsible for α-helix 1 phosphorylation of SMADs, which reduces SMAD interaction with TGFβ/bone morphogenetic protein (BMP) receptor kinase.^15^ NCB-0005 inhibits TGFβ1-induced migration and invasion of lung cancer cells,^14^ but the degree of the EMT inhibition was modest and the underlying molecular mechanism has remained unexplored.

NCB-0846 is a newly identified small-molecule TNIK-inhibitory compound with a backbone structure different from NCB-0005.^16^ NCB-0846 inhibits the transcriptional coactivator function of TNIK by modulating its conformational structure. We have previously revealed that this compound abrogates colorectal cancer stemness through suppression of Wnt target gene expression. NCB-0846 also suppresses the expression of SMAD2, vimentin and EMT-activating transcription factors (TFs) including Wnt targets, Snail and Twist, in colorectal cancer cells.^16^ Given the convergence of TGFβ and Wnt signalling, we assumed that NCB-0846 might also inhibit EMT. Here we report that NCB-0846 blocks the TGFβ/SMAD signalling and EMT induction of lung cancer cells through novel miRNA-mediated silencing of the *TGFBR1* gene.

## Methods

### Ethical issues

All the animal experimental protocols in this study were reviewed and approved by the institutional ethics and recombination safety committees of the National Cancer Center Research Institute (Tokyo, Japan). The number of animals necessary for obtaining reliable results was minimised, with strict attention to animal rights and welfare protection.

### Antibodies and reagents

All antibodies used in this study and their suppliers are listed in Supplementary Table S1. Recombinant human TGFβ1 was from R&D Systems. NCB-0846 and its diastereomer, NCB-0970, have been described previously.^16^

### Cell lines

The human non-small-cell lung cancer (NSCLC) cell lines, A549 and NCI-H2228, were obtained from the Cell Bank of the American Type Culture Collection (ATCC). Cells were maintained in Eagle’s minimum essential medium (Nacalai Tesque) or RPMI1640 medium (Thermo Fisher Scientific) supplemented with 10% heat-inactivated foetal bovine serum (FBS) (Thermo Fisher Scientific) and 1% nonessential amino acid (Thermo Fisher Scientific) in a humidified incubator supplied with 5% CO_2_. Absence of mycoplasma contamination was monitored routinely using the e-Myco VALiD Mycoplasma PCR Detection Kit (iNtRon Biotechnology). None of these cell lines is listed in the International Cell Line Authentication Committee database of cross-contaminated or misidentified cell lines, and all the cell lines were authenticated by short tandem repeat (STR) DNA profiling by the JCRB Cell Bank, National Institutes of Biomedical Innovation, Health and Nutrition (Osaka, Japan).

### Immunoblot analysis

Whole-cell extracts and nuclear protein fractions were prepared using RIPA buffer (Cell Signaling Technology) and Nuclear and Cytoplasmic Extraction Reagents (Thermo Fisher Scientific), respectively. Protein concentration was determined using Direct Detect (Merck). Equal amounts of proteins (10 or 15 µg) were fractionated on 4–12% NuPAGE Bis-Tris gels (Thermo Fisher Scientific) and transferred to Immobilon-P membranes (Merck) as described previously.^17^ Following incubation with primary antibodies (listed in Supplementary Table S1) at 4 °C overnight, the blots were reacted with relevant horseradish peroxidase conjugated anti-mouse or anti-rabbit IgG antibody (Cell Signaling Technology). Blot signals were detected with the Western Lighting ECL Pro (PerkinElmer) and the LAS 4010 system (GE Healthcare), and signal intensity was quantified using the ImageQuant TL software package (GE Healthcare).

### Immunofluorescence microscopy

Cells were seeded at 2.5 or 5.0 × 10^4^ per well onto collagen-coated 8-well culture slides (BioCoat). Following 24-h serum starvation, the cells were cultured with dimethyl sulfoxide (DMSO) alone (control), TGFβ1 (5 ng/ml) and DMSO, TGFβ1 and NCB-0846 (1 µM) or TGFβ1 and NCB-970 (1 µM) for 48 h and fixed in 4% paraformaldehyde. The fixed cells were immunostained with primary antibodies (listed in Supplementary Table S1) as described previously.^18^ Following overnight incubation at 4 °C, the cells were incubated with relevant secondary antibodies conjugated with Alexa Fluor 488 or Alexa Fluor 568 (Thermo Fisher Scientific) and co-stained with TOTO3 (Thermo Fisher Scientific) or phalloidin (Thermo Fisher Scientific) for visualisation of nuclei or filamentous actin, respectively. Cells were examined with the LSM 5 PASCAL laser confocal microscopy system (Carl Zeiss).

### Wound-closure assay

A549 cells were seeded at 5.0 × 10^5^ per well in a 6-well plate and serum-starved for 24 h prior to creation of a scratch on the confluent cell monolayer. The wounded monolayers were then treated with DMSO alone (control), TGFβ1 (5 ng/ml) and DMSO, TGFβ1 and NCB-0846 (3 µM), or TGFβ1 and NCB-970 (3 µM) for 48 h. The degree of migration was determined by measuring the width of cell-free areas in triplicate.

### Automated real-time cell migration assay

Cells were plated (1.0 × 10^4^ cells/well) in triplicate onto a microelectrodes-embedded xCelligence E-culture plate (ACEA Biosciences). When cells reached confluence (indicated by steady-state impedance), scratches were created with sterile P200 micropipette tips and the cells were treated with DMSO alone (control), TGFβ1 (5 ng/ml) and DMSO, TGFβ1 and NCB-0846 (3 µM), or TGFβ1 and NCB-970 (3 µM) for 40 h. The impedance of cell monolayers before and after scratching as well as the recovery of full impedance were monitored in real time with the xCelligence instrument (ACEA Biosciences).

### Cell invasion assay

Cell invasion was assessed using 16-well transwell plates with 8-µm pores (CIM plate 16; ACEA Biosciences) precoated with 20 µl of Matrigel (BD Biosciences) diluted 1:40 in MEM medium. Microelectrodes were located on the underside of membranes in the upper chambers. Medium containing 10% FCS was added to the lower chamber, and cells were seeded into the upper chamber at 4 × 10^4^ per well in serum-free medium. A549 cells were treated with DMSO alone (control), TGFβ1 (5 ng/ml) and DMSO, TGFβ1 and NCB-0846 (3 µM), or TGFβ1 and NCB-970 (3 µM). Impedance of migrated cells was monitored in real time with the xCelligence instrument. Data analysis was carried out using RTCA software 2.0 supplied with the instrument (ACEA Biosciences).

### Quantitative PCR

Total RNA was isolated with a RNeasy Mini Kit or miRNeasy Mini Kit (Qiagen) in accordance with the manufacturer’s instructions. cDNA was prepared using a High-Capacity cDNA reverse transcription kit (Applied Biosystems) or MicroRNA Reverse Transcription Kit (Applied Biosystems). The relative expression of mRNA and miRNA was measured using the TaqMan Gene Expression Assay (Applied Biosystems) or TaqMan MicroRNA Assay (Applied Biosystems), respectively. Amplification was quantified using the comparative threshold cycle (CT) method.^19^ β-Actin (*ACTB*) or *U6* was used as an internal control (Applied Biosystems). Predesigned primer and probe sets are listed in Supplementary Table Supplementary Table S2.

### miRNA microarray analysis

Total RNA was prepared from the A549 cells treated with DMSO alone (control), TGFβ1 (5 ng/ml) and DMSO, TGFβ1 and NCB-0846 (3 µM) or TGFβ1 and NCB-970 (3 µM) using a miRNeasy Mini Kit (Qiagen) and subjected to miRNA expression profiling with the use of an Agilent Human miRNA Microarray kit 8 × 60 K rel.21.0 (Agilent) in accordance with the manufacturer’s protocol. The scanned images were analysed with Feature Extraction Software 11.5.1.1 (Agilent) using default parameters to obtain the background subtracted value. The GeneView files were generated using Agilent’s Feature Extraction software version 11.5.1.1. The microarray data were deposited in the NCBIs Gene Expression Omnibus database under the accession number GSE95766 (released on March 08, 2017).

### Oligonucleotide transfection

siRNA targeting *TGFBR1* (siTβRI) and a nonspecific negative control (siNC) were obtained from Applied Biosystems (Supplementary Table S2). All miRNA mimics and inhibitors as well as their controls were purchased from Applied Biosystems (Supplementary Table S2). siTβRI or siNC was transfected into cells at a final concentration of 5 nM with Lipofectamine 2000 (Invitrogen), and the transfection medium was replaced after 12-h serum starvation. The cells were then incubated with or without TGFβ1 (5 ng/ml) for 48 h. All mimics and inhibitors were transfected with Lipofectamine 2000 at a final concentration of 50 nM and incubated at 37 °C for 48 h.

### Luciferase reporter gene assay

A549 cells transfected with a luciferase reporter plasmid carrying three copies of a SMAD-binding element (SBE), pGL4.48-*luc2P*/SBE/Hygro (Promega), were treated with DMSO (control) or TGF-β1 (2 ng/ml) for 60 min and with NCB-0846 or NCB-970 at various concentrations for 5 h, and their luciferase activities were measured.

### Evaluation of metastatic potential

Eight-week-old male severe combined immunodeficiency (SCID) mice (C.B-17/Icr-scid; Charles River) were housed in open-top cages under a 12-h light-dark cycle with free access to standard chow and water ad libitum and maintained in a specific pathogen-free environment. A549 cells were serum-starved for 24 h and stimulated with TGF-β (5 ng/ml) and DMSO (control), NCB-0846 (3 µM) or NCB-970 (3 µM) for 48 h, and 5.0 × 10^5^ cells in 100 µL of PBS were injected into the anesthetised mice through the tail vein. Seven weeks after the injection of cells treated as indicated, the mice were euthanatised by cervical dislocation, and the digital images of entire lung tissue sections (HE stained) were captured with a BZ-X700 auto-microscope (Keyence). The areas occupied by metastases were quantified using the Hybrid Cell Count software (Keyence).

### Statistical analysis

Data were expressed as mean (±SD) of three independent experiments and evaluated using Student *t*-test (unless otherwise stated). Differences were considered to be significant at *P* < 0.05.

## Results

### NCB-0846 blocks TGFβ1-induced EMT

**Fig. 1:**
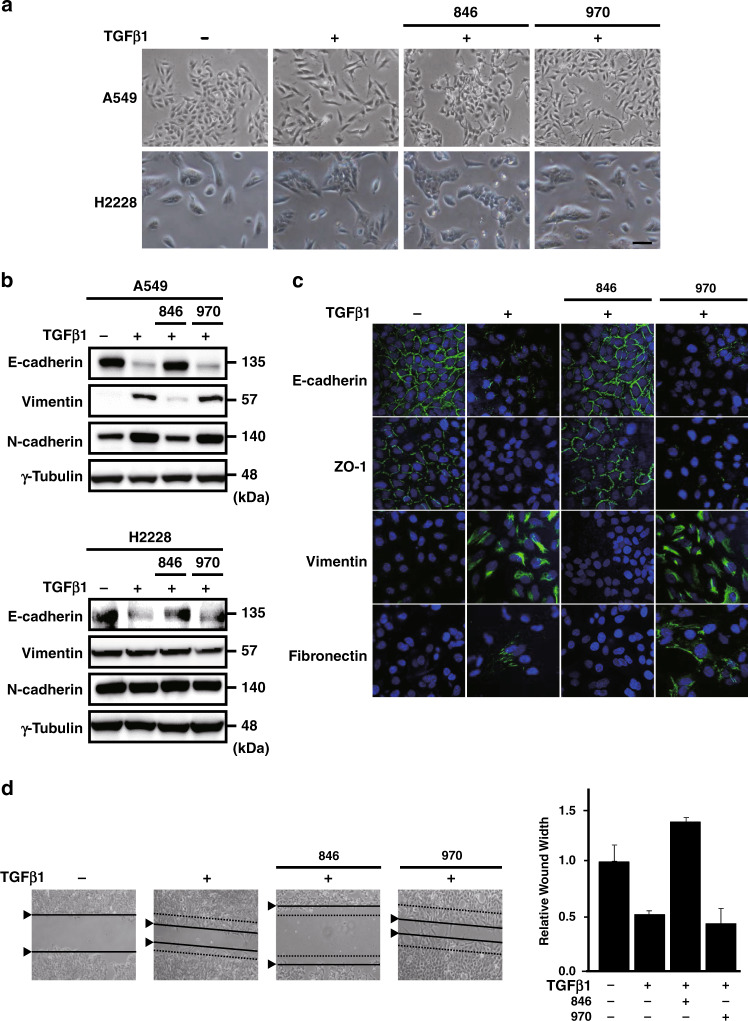
NCB-0846 inhibits TGFβ1-triggered EMT. **a** Morphological conversion of A549 (upper panels) and H2228 (lower panels) cells. Following 24-h serum starvation, cells were treated with DMSO alone (control), TGFβ1 (5 ng/mL) and DMSO, TGFβ1 and NCB-0846 (3 µM), or TGFβ1 and NCB-0970 (3 µM) for 48 h and photographed. Scale bar, 100 µm. **b** Immunoblot analysis of epithelial (E-cadherin) and mesenchymal cell markers (vimentin and N-cadherin) and γ-tubulin (loading control) in A549 (upper panels) and H2228 (lower panels) cells treated as indicated. **c** Immunofluorescence confocal microscopy of epithelial (E-cadherin and ZO-1) and mesenchymal cell markers (vimentin and fibronectin) in A549 cells treated as indicated. The nuclei were stained with TOTO3 (blue). Scale bar, 20 μm. **d** Cell migration evaluated by a wound-closure assay. Dotted and solid lines indicate the edges of wounds immediately and 48 h after creation of the wounds, respectively. The width of wounds relative to their baseline (set to one) is shown in the bar graph (right).

We tested the effects of a small-molecule TNK modulator, NCB-0846, on TGFβ1-induced EMT in two NSCLC cell lines (A549 and H2228). NCB-0846, but not its inactive diastereomer NCB-0970,^16^ inhibited the TGFβ1-induced morphological conversion from the cobblestone-like epithelial phenotype to the spindle cell-like mesenchymal phenotype in A549 cells and modestly in H2228 cells (Fig. 1a). This inhibitory effect of NCB-0846 on the TGFβ1-triggered EMT of A549 cells was supported by restoration of E-cadherin expression and suppression of the mesenchymal cell markers, vimentin and N-cadherin, whereas NCB-970 had no such effect on the expression of these EMT markers. The marker switching was not obvious in H2228 cells (Fig. 1b). Furthermore, NCB-0846, but not NCB-0970, upregulated the gene expression of E-cadherin (*CDH1*) and downregulated that of N-cadherin (*CDH2*) and vimentin (*VIM*) in A549 cells (Supplementary Fig. S1). Immunofluorescence microscopy supported these observations. NCB-0846 restored the cell membrane localisation of E-cadherin and a tight junction protein, zonula occludens-1 (ZO-1), and suppressed the expression of mesenchymal cell markers (Fig. 1c). We were unable to detect any cells that showed dual positivity for E-cadherin and vimentin. The proper distribution of ZO-1 in NCB-0846-treated A549 cells indicated the restoration of epithelial cell polarity. NCB-0846, but not NCB-0970, decreased the TGFβ1-induced motility of A549 cells in a conventional wound-closure assay (Fig. 1d) and also as demonstrated by automated real-time cell tracing (Supplementary Fig. S2). Real-time trans-Matrigel cell monitoring also demonstrated complete inhibition of TGFβ1-induced cell invasion by NCB-0846 (Supplementary Fig. S3). However, the motility and invasion of A549 cells treated with TGFβ1 and NCB-0864 was reduced to below that with the DMSO control, indicating the cytotoxic effect of NCB-0846.

### Abrogation of stemness is not involved in EMT inhibition

NCB-0846, but not NCB-0970, inhibited the expression of TNIK as well as the expression of Wnt target gene products, including Axin1, Axin2 and cMyc, in A549 lung cancer cells (Supplementary Fig. S4a). NCB-0846 abrogates the stemness of colorectal cancer by blocking the transcription of Wnt target genes.^16,20^ We had previously observed that NCB-0846 downregulated the expression of stem cell markers, such as CD133, CD44 and aldehyde dehydrogenase-1 (ALDH1) in colorectal cancer cells,^16^ whereas such an effect was barely evident in the lung cancer cells (Supplementary Fig. S4b), suggesting that the effects of NCB-0846 are cell context-dependent and that its cancer stemness-inhibitory activity is not necessary for EMT suppression.

### Effects on SMAD signalling

Canonical TGFβ1 signalling is initiated by binding of TGFβ1 to the TGFβ type-II receptor (TGFβRII). TGFβRII is a serine/threonine protein kinase, which transphosphorylates and activates the TGFβ type-I receptor (TGFβRI). The activated TGFβRI recruits and phosphorylates Sma- and Mad-Related Proteins-2 and -3 (SMAD2/3), thus facilitating the formation of a complex with SMAD4, and translocation of the complex into the nucleus,^21,22^ where the SMAD complex mediates the transcription of EMT-activating transcription factors; including the Snail, Twist and zinc finger E-box-binding homeobox (ZEB) families.^23^

**Fig. 2:**
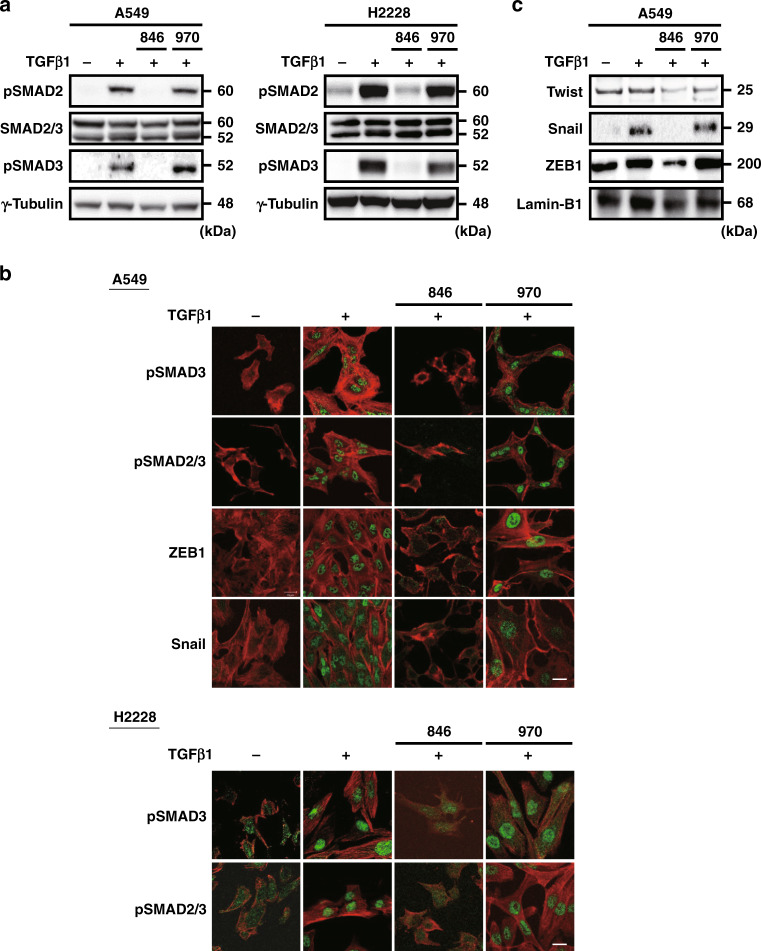
NCB-0846 blocks TGFβ1-mediated SMAD activation. **a** Immunoblot analysis of SMAD2 phosphorylated at the 465/467 serine residues (pSMAD2), total SMAD2/3, SMAD3 phosphorylated at the serine 465/467 residues (pSMAD3), and γ-tubulin (loading control) in A549 (left) and H2228 (right) cells treated as indicated. **b** Immunofluorescence confocal microscopy of pSMAD3, phosphorylated SMAD2 (Ser465/467)/SMAD3 (Ser423/425) (pSMAD2/3), ZEB1 and Snail in A549 cells and pSMAD3 and pSMAD2/3 in H2228 cells treated as indicated. Filamentous actin is co-stained with phalloidin (red). Scale bar, 20 μm. **c** Immunoblot analysis of EMT-related transcription factors (Twist, Snail and ZEB1) and Lamin-B1 (loading control) in the nuclear protein extracts prepared from A549 cells treated as indicated.

NCB-0846, but not NCB-0970, completely inhibited TGFβ1-driven phosphorylation of SMAD2 and SMAD3 (Fig. 2a) as well as their subsequent nuclear translocation (Fig. 2b) in A549 cells. Paradoxically, the inhibition of SMAD phosphorylation and nuclear translocation in H2228 cells occurred despite the lack of TGFβ1-driven EMT induction. In addition, a luciferase reporter assay for SMAD-binding element (SBE) confirmed that NCB-0846 dose-dependently inhibited TGFβ1-driven SMAD-mediated transcriptional activity (Supplementary Fig. S5). Consistently, NCB-0846 suppressed the expression of Snail completely and that of ZEB1 modestly in A549 cells (Fig. 2c).

### Repression of TGFBR1 gene expression

**Fig. 3:**
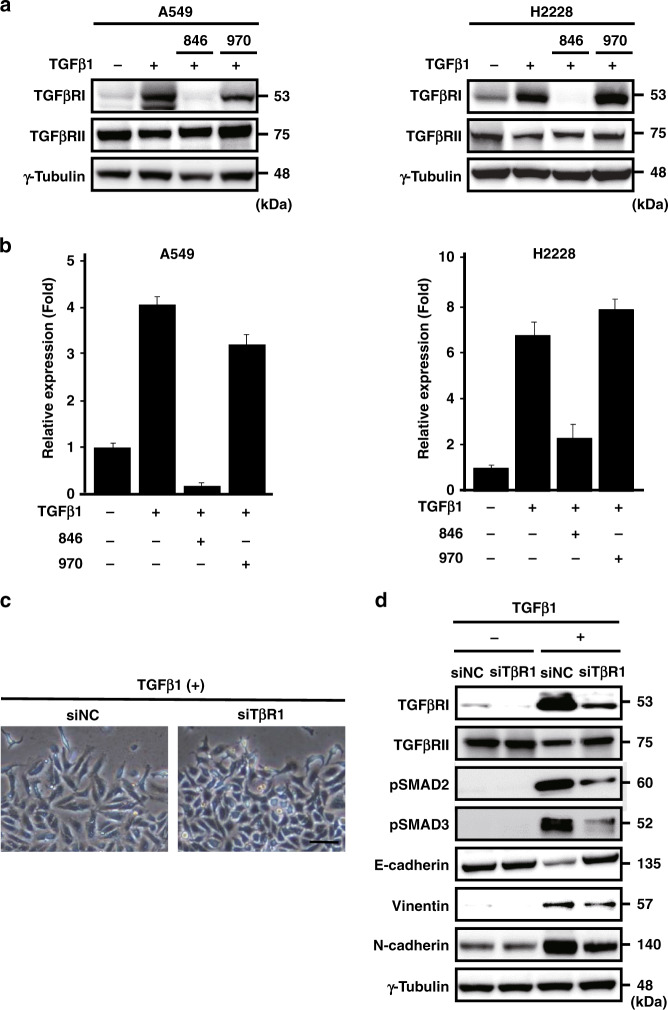
NCB-0846 downregulates the *TBFBRI* transcript. **a** Immunoblot analysis of TGFβRI, TGFβRII and γ-tubulin (loading control) in A549 (left) and H2228 (right) cells treated as indicated. Note that NCB-0846 specifically inhibited the expression of TGFβRI protein. **b** Relative expression of the *TGFBR1* gene (normalised to *ACTB*) in A549 and H228 cells treated with DMSO alone (control), TGFβ1 (5 ng/mL) and DMSO, TGFβ1 and NCB-0846 (3 µM) or TGFβ1 and NCB-0970 (3 µM) for 48 h. The values for cells treated with DMSO alone are set to one. Error bars represent standard deviations (SD) of triplicate experiments. **c** Morphology of *TGFBR1*-knockdown cells. A549 cells transfected with 50 nM control siRNA (siNC) or siRNA to *TGFBR1* (siTβRI) were incubated with TGFβ1 (5 ng/mL) for 48 h and photographed. Scale bar, 100 μm. **d** Immunoblot analysis of TGFβRI, TGFβRII, pSMAD2, pSMAD3, E-cadherin, vimentin, N-cadherin and γ-tubulin (loading control) in A549 cells transfected with control siRNA (siNC) or siRNA to *TGFBR1* (siTβRI) and incubated without (−) or with (+) TGFβ1 (5 ng/mL) for 48 h.

To clarify the mechanism underlying the inhibitory effect of NCB-0846 on TGFβ1-induced SMAD signalling, we looked at its upstream receptors TGFβRI and TGFβRII. TGFβ1 did not affect the expression of TGFβRII, but strongly induced the expression of TGFβRI (Fig. 3a). NCB-0846 inhibited the TGFβ1-mediated induction of TGFβRI protein (Fig. 3a) and *TGFBR1* gene expression (Fig. 3b).

To confirm the involvement of TGFβRI in the EMT inhibition by NCB-0846, we introduced a small interfering RNA (siRNA) recognising *TGFBR1* (siTβR1) into A549 cells. siTβR1, but not control RNA (siNC), restored the cobblestone-like appearance and cell-to-cell adhesion of TGFβ1-treated cells (Fig. 3c). *TGFBR1* knockdown restored the cell membrane localisation (Supplementary Fig. S6) of E-cadherin, downregulated the expression of phosphorylated SMAD2/3 (pSMAD2/3) (Fig. 3d), and inhibited the nuclear localisation of pSMAD2/3, ZEB1 and Snail proteins (Supplementary Fig. S6). These results confirmed that NCB-0846 inhibited EMT through downregulation of *TGFBR1* gene expression.

### *TGFBR1*-targeting miRNAs

**Fig. 4:**
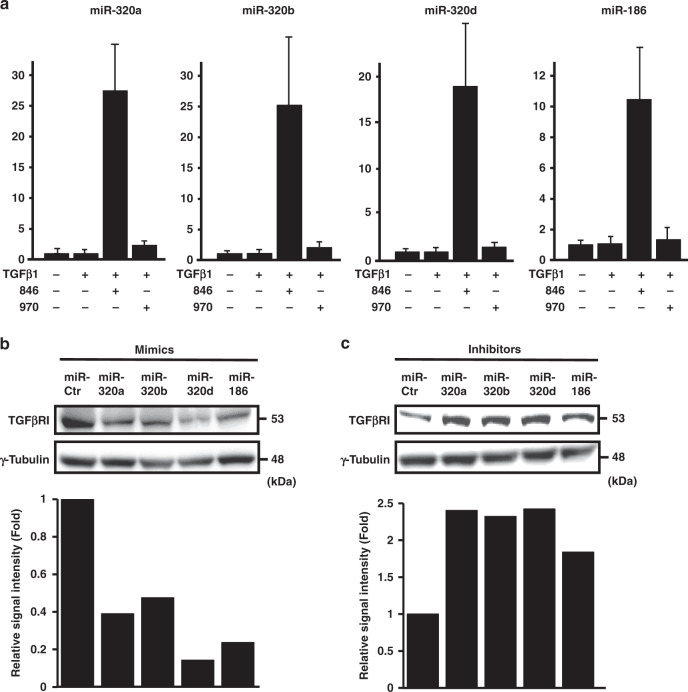
miR-320s and miR-186 target *TGFBRI*. **a** The induction of miR-320s and miR-186 by NCB-0846. A549 cells were treated with DMSO alone (control), TGFβ1 (5 ng/mL) and DMSO, TGFβ1 and NCB-0846 (3 µM), or TGFβ1 and NCB-0970 (3 µM), and the expression of indicated miRNAs was quantified in triplicate by qRT-PCR. The values for cells treated with DMSO alone are set to one. Error bars represent SD. **b** Reduced expression of TGFβRI protein by introduction of miR-320s and miR-186 mimics. A549 cells were transfected with the indicated miRNA mimics, and 48 h later TGFβRI protein was detected by immunoblotting (upper panels, γ-tubulin as a loading control). The bottom panel indicates signal intensity relative to the control mimic (miR-Ctr). **c** Increased expression of TGFβRI protein by introduction of miR-320s and miR-186 inhibitors. A549 cells were transfected with the indicated miRNA inhibitors, and 48 h later TGFβRI protein was detected by immunoblotting (upper panels, γ-tubulin as a loading control). The bottom panel indicates signal intensity relative to the control inhibitor (miR-Ctr).

Noncoding microRNAs (miRNAs) have been increasingly recognised as key players in gene silencing. miRNAs bind to specific target sequences present in the 3’ untranslated regions (UTR) of transcripts and post-transcriptionally silence the corresponding gene expression.^24^ To better understand the mechanism underlying the reduction of *TGFBR1* expression by NCB-0846, we profiled the miRNA expression of A549 cells treated with TGFβ1 and NCB-0846 or NCB-0970 using oligonucleotide miRNA microarrays (Supplementary Table S3). Among 61 miRNAs whose expression was increased > 2-fold by NCB-0846 treatment, we found using two publicly available algorithms (TargetScan; http://www.targetscan.org and TargetSpy; http://webclu.bio.wzw.tum.de/targetspy) that miR-186-5p and miR-320a, 320b, and 320d were predicted to bind to the 3’UTR of *TGFBR1* (Supplementary Fig. S7). Sharp induction of miR-186-5p and miR-320 (a, b and d) by NCB-0846 was confirmed by real-time quantitative reverse transcription polymerase chain reaction (qRT-PCR) (Fig. 4a). Transient transfection with miR-320 (a, b and d) and miR-186 mimics resulted in reduced expression of TGFβRI (Fig. 4b), whereas transient transfection with inhibitors of miR-320 (a, b and d) and miR-186 increased the expression of TGFβRI protein (Fig. 4c). These data indicated that NCB-0846 repressed the expression of TGFβRI at least partially through the induction of these miRNAs.

### Potential of NCB-0846 to inhibit metastasis

**Fig. 5:**
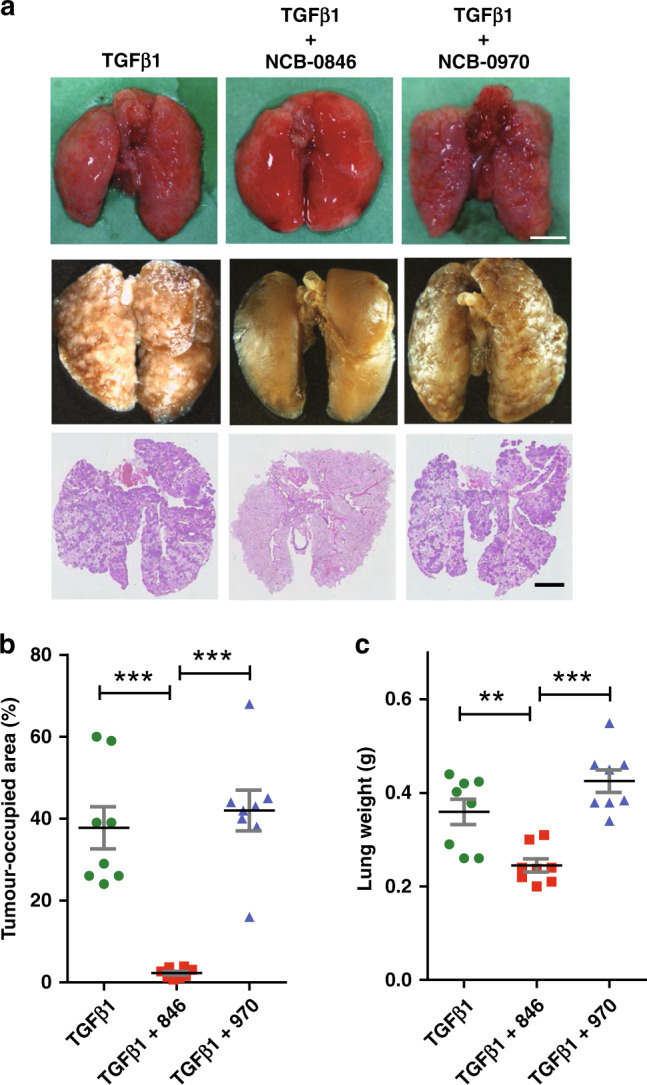
NCB-0846 inhibits metastatic potential. **a** Representative images of lungs (from top to bottom: unfixed, formalin-fixed, and HE-stained tissue sections) resected from mice 7 weeks after injection of A549 cells treated with TGF-β1 (5 ng/mL) and DMSO, TGF-β1 and NCB-0846 (3 µM) or TGF-β1 and NCB-0970 (3 µM) for 48 h. Scale bar represents 5 mm. **b** Summed areas of metastases relative to the total lung areas of mice injected with A549 cells treated as described in the legend to Fig. 5a. Error bars represent SD. ***P < 0.001. **c** Wet lung weights of mice injected with A549 cells treated as described in the legend to Fig. 5a. Error bars represent SD. ***P < 0.001; **P < 0.01.

Finally, we explored whether the EMT inhibitory activity of NCB-0846 affects metastasis. A549 cells were treated with TGFβ1 in the absence or presence of either NCB-0846 or NCB-0970 for 48 h in vitro, and then injected into immunodeficient mice (eight per group) via the tail vein. Seven weeks after injection, the mice were sacrificed, and their lung metastases were digitally quantified in tissue sections (Fig. 5a). This animal experiment is used mainly to evaluate the trans-endothelial migration/extravasation capability of cancer cells embolised in peripheral lung vessels immediately after systemic injection.

Metastatic lesions occupied 38% of the lung area in mice injected with TGFβ1-treated cells but were significantly (*P* < 0.001) reduced in the lungs of mice injected with NCB-0846-treated cells (Fig. 5b). We confirmed the complete absence of metastasis by microscopic inspection of tissue sections. In parallel, the average lung weights of mice injected with NCB-0846-treated cells were significantly decreased due to lack of the pulmonary metastatic burden, compared with those of mice injected with cells that had been treated with DMSO (control) or NCB-0970 (Fig. 5c). These results support the notion that inhibition of EMT by NCB-0846 compromises the TGFβ1-induced metastatic potential of lung cancer cells.

## Discussion

TNIK is a member of the STE20 (sterile 20) serine/threonine protein kinase family, which comprises TNIK, misshapen-like kinase 1 (MINK1), and mitogen-activated protein kinase kinase kinase kinase 4 (MAP4K4).^25–27^ We and others almost simultaneously identified TNIK as a component of the β-catenin and TCF4 transcriptional complex in human and mouse, respectively, through similar large-scale proteome analyses.^11,28^ TNIK was essential for colorectal cancer growth and the transactivation of Wnt target genes (such as *MYC*, *AXIN2* and *CD44*) by TCF4.^12^ Increased expression of TNIK is associated with an unfavourable clinical outcome in patients with pancreatic, colorectal and hepatocellular carcinomas,^29–31^ and the *TNIK* gene is amplified in 7% of gastric cancers.^32^ A recent meta-analysis of cancer genome-sequencing studies identified TNIK as a driver oncogene.^33^ Based on these experimental and clinical findings, various classes of TNIK inhibitors have been developed.^20,34^

In this study we found that the activity of TGFβ signalling was dynamically regulated by the expression level of a cell surface receptor of TGFβ1, TGFβRI (Fig. 3). Stimulation of NSCLC cells with TGFβ1 induced the expression of TGFβRI, and a small-molecule TNIK inhibitor, NCB-0846, downregulated the *TGFBR1* gene expression and inhibited the TGFβ1-induced EMT of NSCLC cells. NCB-0970 has a similar inhibitory profile over the human kinome except for STE20 member kinases.^16^ We carefully included the diastereomer as a negative control in all of the experiments we conducted to eliminate any off-target effects of NCB-0846. Furthermore, the EMT inhibition was recapitulated by siRNA-mediated knockdown of *TGFBR1* (Fig. 3c, d and Supplementary Fig. S6). We also found that the silencing of the *TGFBR1* gene was mediated at least partially by miR-186-5p and miR-320a, 320b and 320d (Fig. 4) that target the 3’ UTR of *TGFBR1* transcript (Supplementary Fig. S7).

The TGFβ/SMAD signalling pathway is the central regulator of EMT and has been considered a good target for drug development. As phosphorylation of SMAD proteins by TGFβRI is essential for downstream SMAD signalling, several small-molecule inhibitors have been developed to pharmacologically inhibit the kinase activity of TGFβRI.^35,36^ Galunisertib (LY2157299) is an orally available small-molecule kinase inhibitor of TGFβRI, and its anti-tumour activity has been confirmed in animal models of various cancers. Galunisertib in combination with gemcitabine significantly improved the overall survival of patients with unresectable pancreatic cancer over gemcitabine monotherapy.^37^ NCB-0846 blocked TGFβ/SMAD signalling by downregulating *TGFBRI* gene expression. This novel mode of action (MOA) distinguishes the compound from the preceding TGFβRI/ALK5 kinase inhibitors.

Several lines of evidence indicate the involvement of Wnt signalling in TGFβ-induced EMT. TNIK is an essential regulator of Wnt signalling, and two small-molecule TNIK inhibitors with different chemical structures (NCB-0005 and NCB-0846) reproducibly blocked the TGFβ/SMAD signalling and EMT of NSCLC cells, confirming the feasibility of targeting TNIK. Pharmacological TNIK inhibition would be potentially applicable to the management of various EMT-associated disorders; not only metastasis, but also cancer stemness and chemotherapy resistance. NCB-0846 is now under preclinical development aimed at investigational new drug (IND) application.

## Supplementary information

Supplementary Figures S1-7

Supplementary Tables S1-3

## Data Availability

The datasets generated and/or analysed during the present study are not publicly available but may be available from the corresponding author on reasonable request. The miRNA microarray data have been deposited in the NCBIs Gene Expression Omnibus database under the accession number GSE95766.
