# Supplementary material for: Pharmacological blockage of transforming growth factor-β signalling by a Traf2- and Nck-interacting kinase inhibitor, NCB-0846
Source: Br J Cancer. 2020 Nov 27;124(1):228–36. doi: 10.1038/s41416-020-01162-3 (PMC7782820; doi:10.1038/s41416-020-01162-3)
Supplement: Supplementary file 1 — Supplementary Figures S1-7 [file 41416_2020_1162_MOESM1_ESM.pdf]

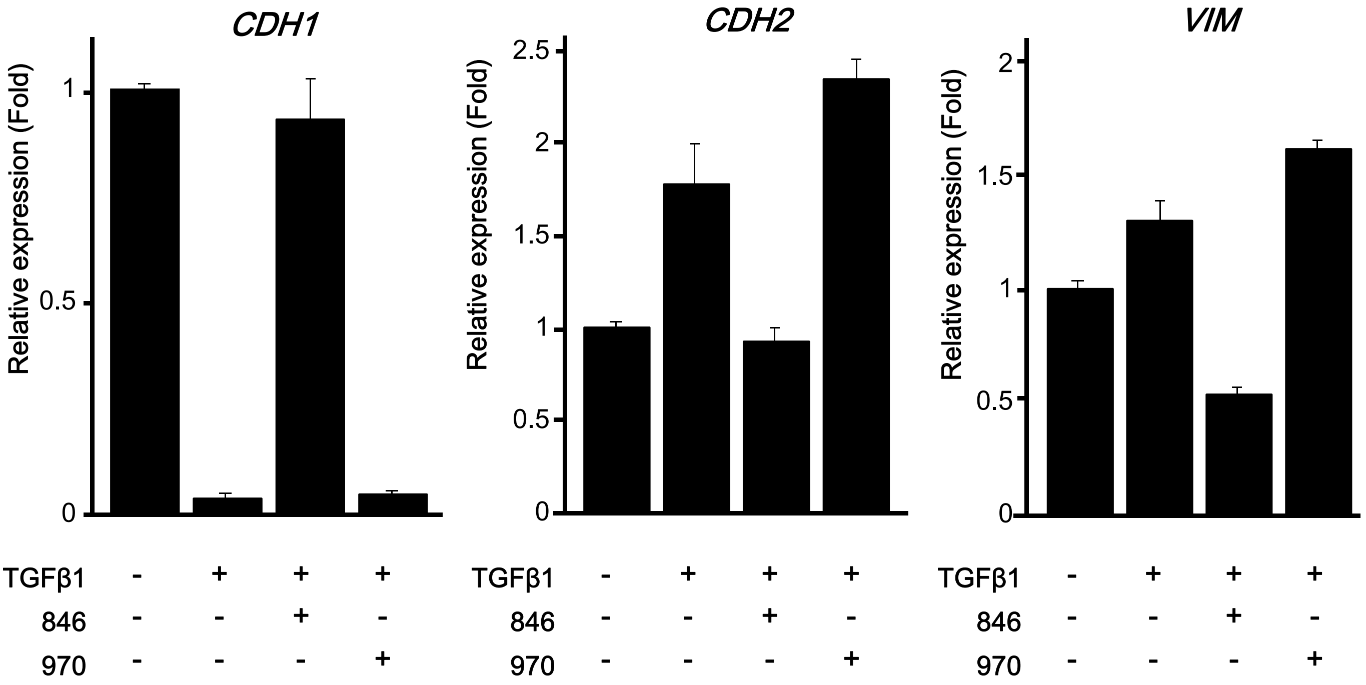

Supplementary Figure S1. NCB-0846 regulates the expression of EMT-related genes.
A549 cells were treated with DMSO alone (control), TGFβ1 (5 ng/mL) and DMSO, TGFβ1 and NCB-0846 (3 μM), or TGFβ1 and NCB-0970 (3 μM) for 48 hours, and the relative expression levels of the E-cadherin (*CDH1*), N-cadherin (*CDH2*), and vimentin (*VIM*) genes were quantified (normalized to *ACTB*) in triplicate by qRT-PCR. The mean values for cells treated with DMSO alone are set to one. Error bars represent SD.

Supplementary Figure S1

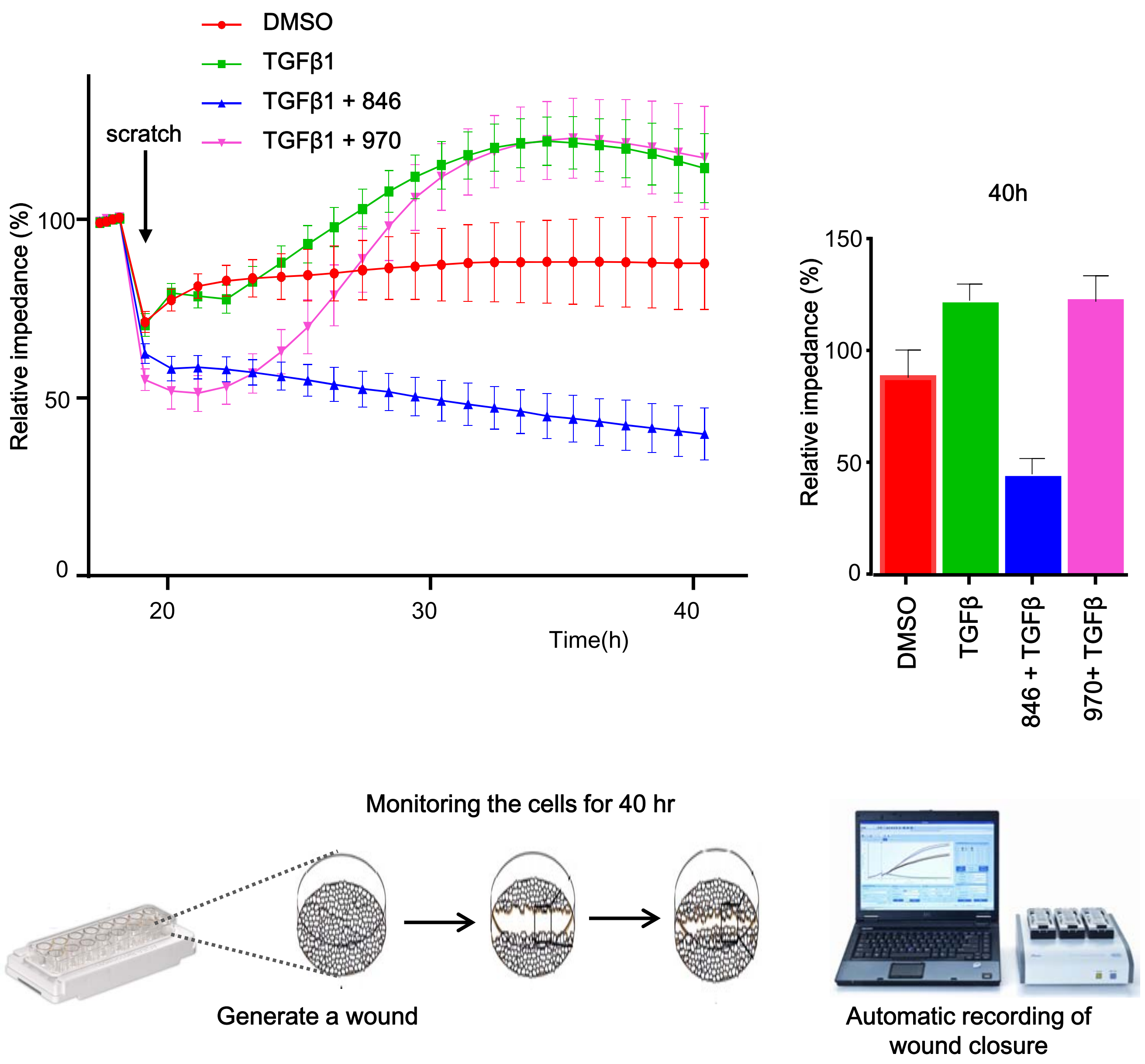

Supplementary Figure S2. Real-time monitoring of cell migration using the xCELLigence system.

The migration of A549 cells into denuded wound areas was monitored by measuring the electrical impedance of cell monolayers in the presence of DMSO (control), TGFβ1 (5 ng/mL) and DMSO (vehicle), TGFβ1 and NCB-0846 (3 μM), or TGFβ1 and NCB-0970 (3 μM) for 40 hours. The impedance before scratching is set at 100%. Error bars indicate SD of triplicate experiments.

Supplementary Figure S2

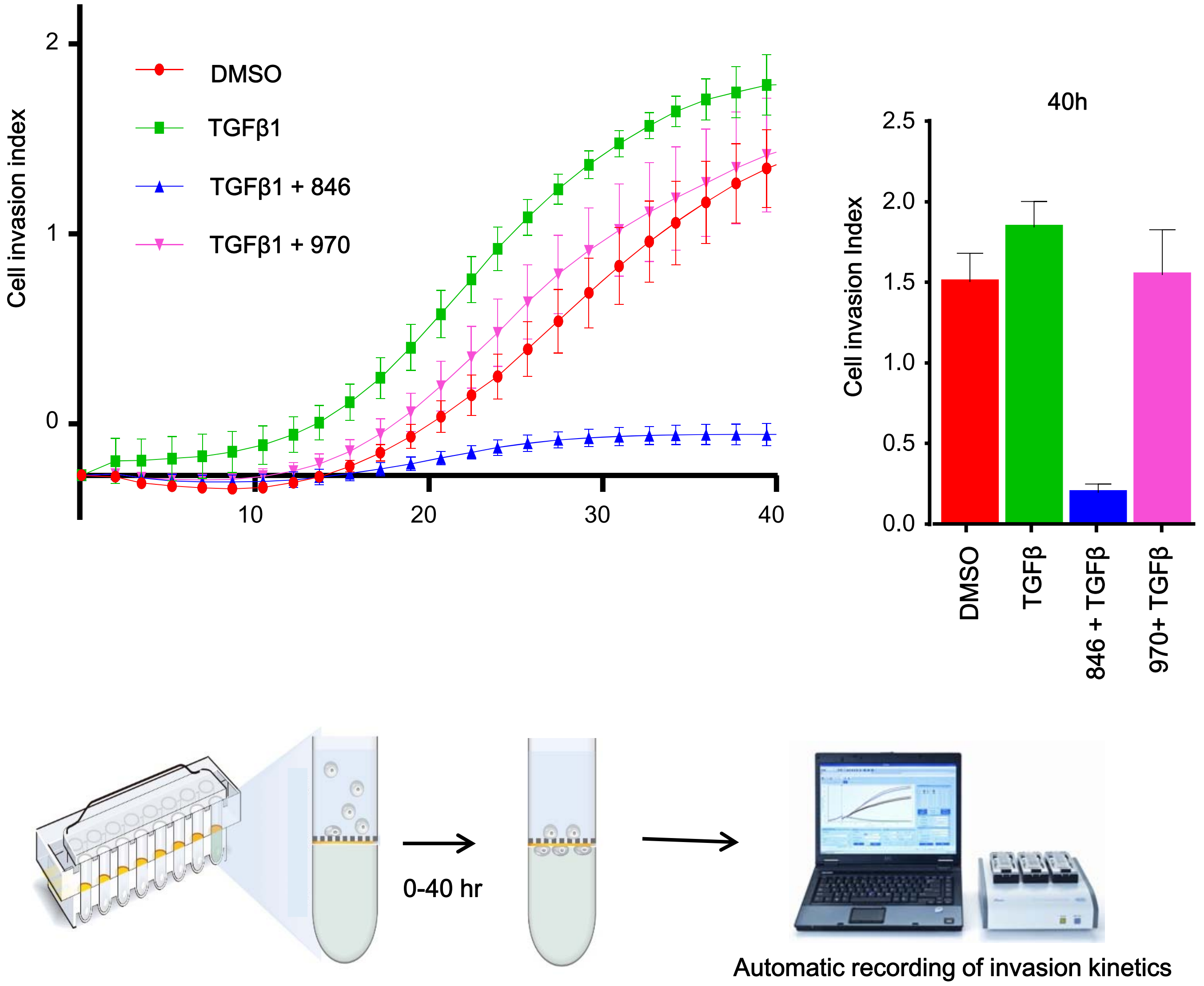

Supplementary Figure S3. Real-time monitoring of cell invasion using the xCELLigence system.

A549 cells were seeded onto a Cell Invasion and Migration (CIM)-plate pre-coated with Matrigel in triplicate, and cell invasion was monitored for 40 hours in the presence of DMSO (control), TGFβ1 (5 ng/mL) and DMSO (vehicle), TGFβ1 and NCB-0846 (3 μM), or TGFβ1 and NCB-0970 (3 μM). Error bars indicate SD of triplicate experiments.

Supplementary Figure S3

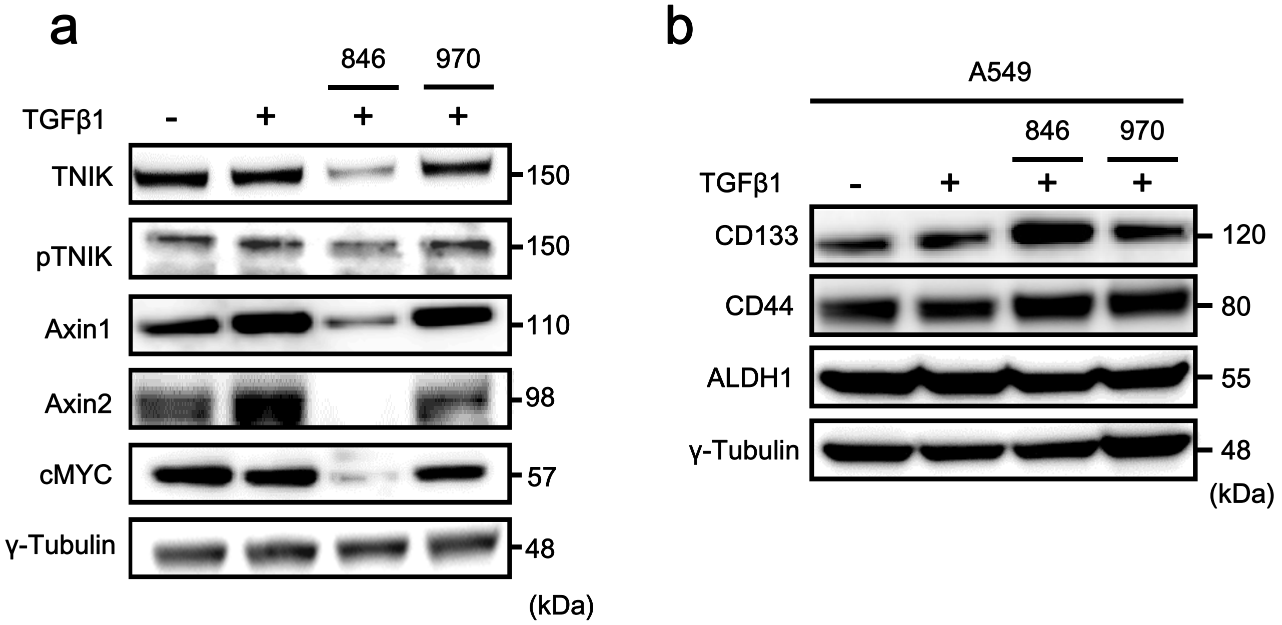

Supplementary Figure S4. Effects of NCB-0846 on the expression of Wnt signal target gene products and cancer stem cell markers.

(a) Immunoblot analysis of TNIK, TNIK phosphorylated at the 465/467 serine residues (pTNIK), Axin1, Axin2, cMYC, and γ-tubulin (loading control) in A549 cells treated with DMSO alone (control), TGFβ1 (5 ng/mL) and DMSO, TGFβ1 and NCB-0846 (3 μM), or TGFβ1 and NCB-0970 (3 μM) for 48 hours.

(b) Immunoblot analysis of cancer stem cell markers (CD133, CD44, and ALDH1) in A549 cells treated as indicated.

Supplementary Figure S4

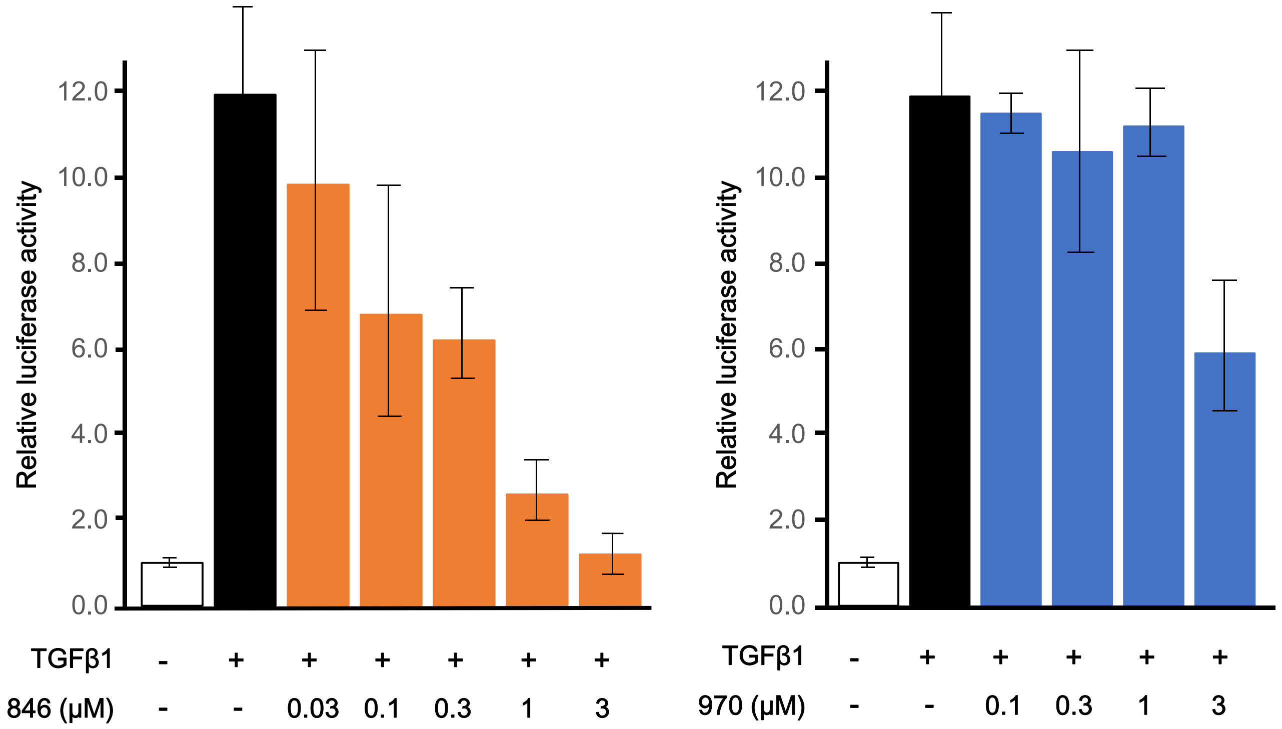

Supplementary Figure S5. NCB-0846 blocks TGFβ1-induced SMAD transcriptional activity.
A549 cells transfected with SBE-luciferase reporter plasmid were treated with DMSO (control) or TGF-β1 (2 ng/ml) for 60 minutes and with NCB-0846 or NCB-970 at the indicated concentrations for 5 hours, and their luciferase activities were measured. All the quantitative data are shown as the mean ± SD of three independent experiments.

Supplementary Figure S5

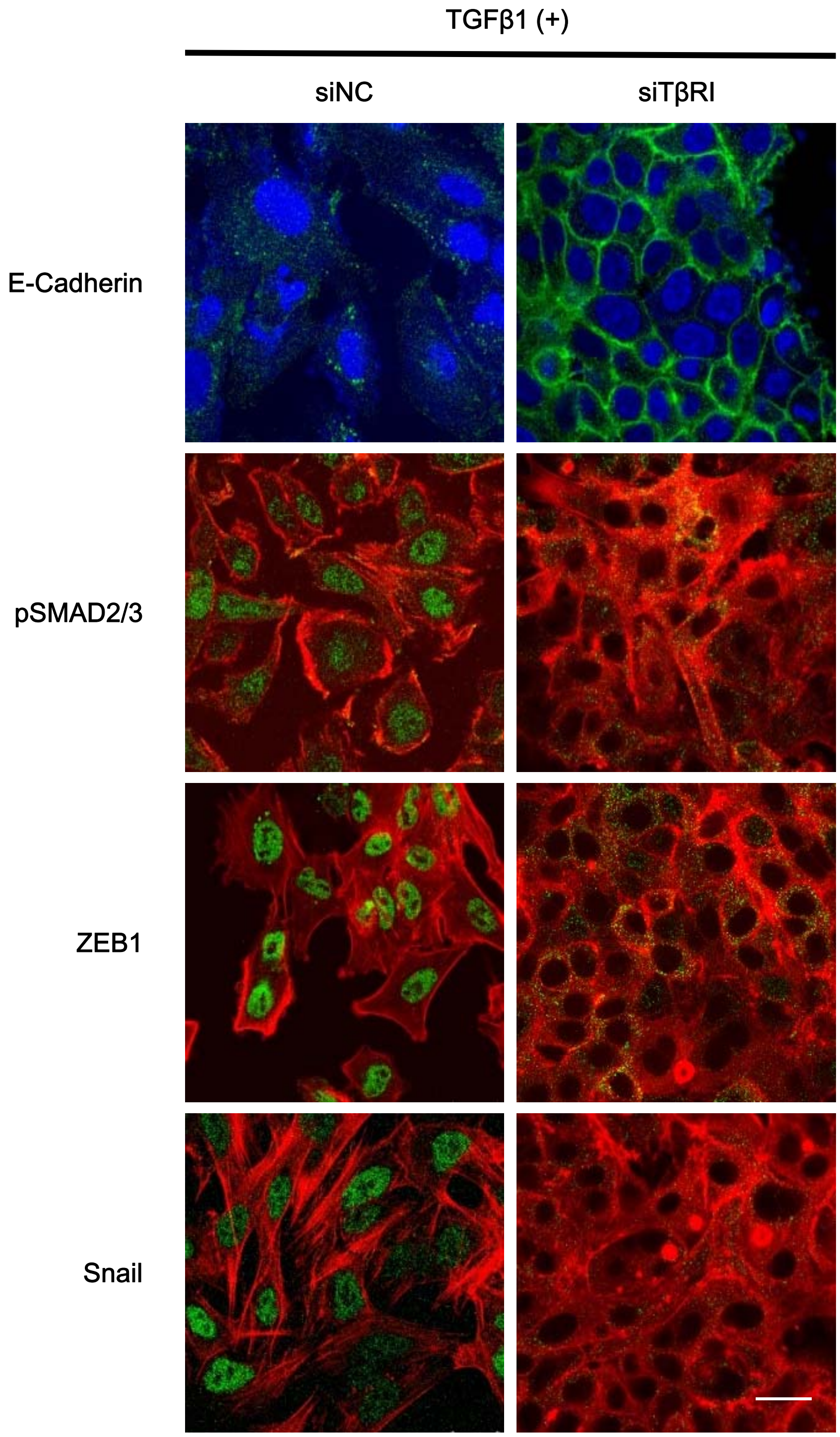

Supplementary Figure S6. *TGFBR1* knockdown affects the localization of EMT-related proteins.

Following 12-hour serum starvation, A549 cells were transfected with control siRNA (siNC) or siRNA for *TGFBR1* (siTβRI) and treated with TGFβ1 (5 ng/ml) for 48 hours. The expression and localization of E-cadherin, pSMAD2/3, ZEB1, and Snail were determined by immunofluorescence microscopy (green). The nuclei are stained with TOTO-3 (blue), and filamentous actin is stained with phalloidin (red). Scale bar represents 20 μm.

Supplementary Figure S6

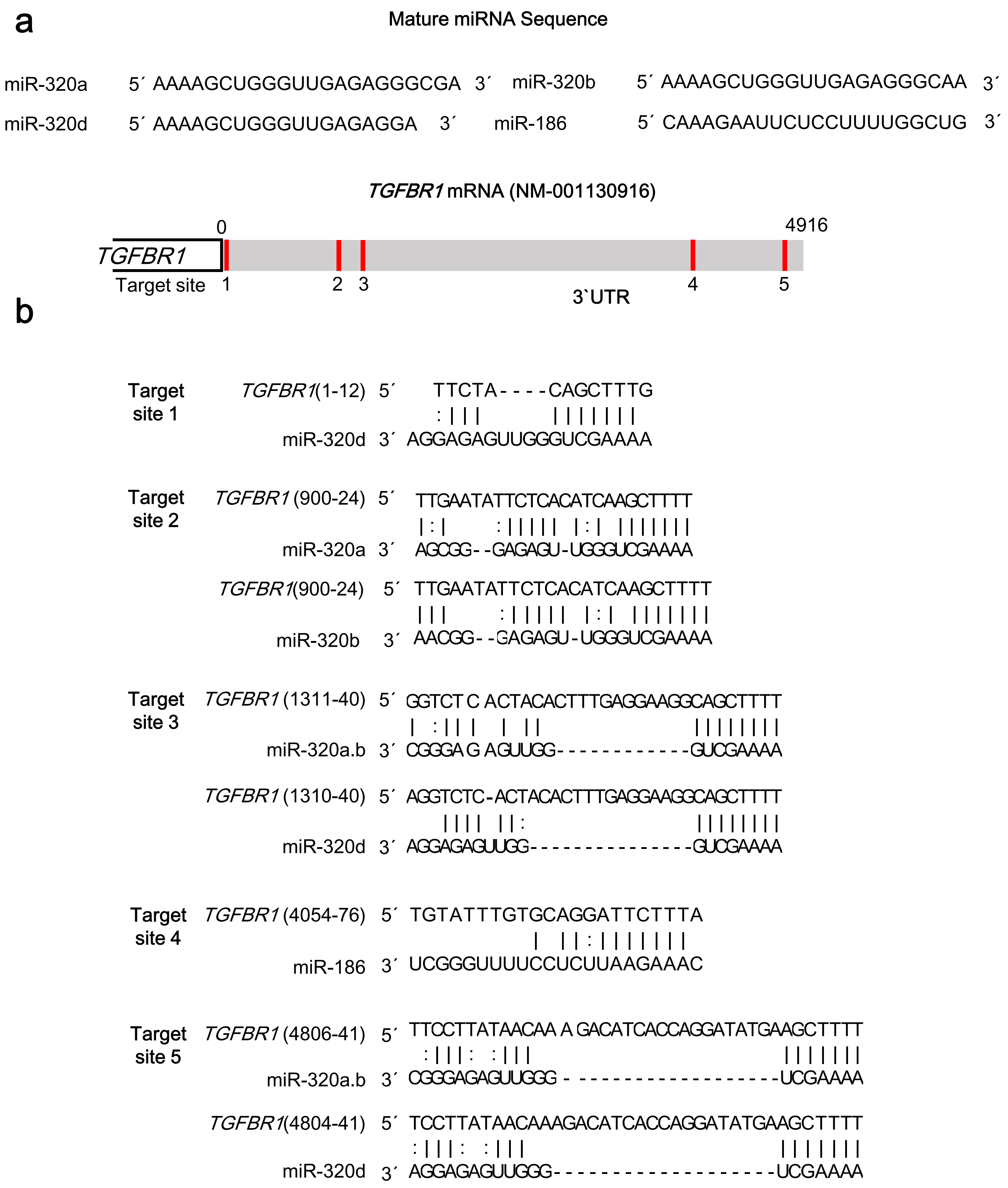

Supplementary Figure S7. *TGFBR1*-targeting miRNAs

(a) Sequences of mature miR-320 (a, b, and d) and miR-186 and schematic representation of their predicted binding sites to the 3′-UTR of the *TGFBR1* gene.

(b) Alignment of the 3′-UTR sequences of the *TGFBR1* gene with miR-320 (a, b, and d) and miR-186.

Supplementary Figure S7
