# Supplementary material for: Pharmacological blockage of transforming growth factor-β signalling by a Traf2- and Nck-interacting kinase inhibitor, NCB-0846
Source: Br J Cancer. 2020 Nov 27;124(1):228–36. doi: 10.1038/s41416-020-01162-3 (PMC7782820; doi:10.1038/s41416-020-01162-3)
Supplement: Supplementary file 2 — Supplementary Tables S1-3 [file 41416_2020_1162_MOESM2_ESM.pdf]

Supplementary Table S1. Primary antibodies used in this study.

Figure		Application	Antigen	Species	Clone	Resource	Catalog #	Dilution
**Figure 1**	**b**	IB	E-cadherin	Mouse monoclonal	36	BD Biosciences	610182	250
		IB	Vimentin	Rabbit monoclonal	D21H3	CST	5741	1000
		IB	N-cadherin	Rabbit monoclonal	D4R1H	CST	13116	1000
		IB	γ-Tublin	Mouse monoclonal	GTU-88	SIGMA	T6557	1000
	**c**	IF	E-cadherin	Mouse monoclonal	36	BD Biosciences	610182	50
		IF	ZO-1	Mouse monoclonal	ZO1-1A12	Thermo Fisher Scientific	33-9100	100
		IF	Vimentin	Rabbit monoclonal	D21H3	CST	5741	100
		IF	Fibronectin	Mouse monoclonal	10/Fibronectin	BD Biosciences	610077	100
**Figure 2**	**a**	IB	pSMAD2	Rabbit monoclonal	138D4	CST	3108	500
		IB	pSMAD3	Rabbit monoclonal	C25A9	CST	9520	500
		IB	SMAD2/3	Rabbit monoclonal	D27F4	CST	8828S	500
		IB	γ-Tublin	Mouse monoclonal	GTU-88	SIGMA	T6557	1000
	**b**	IF	pSMAD3	Rabbit monoclonal	C25A9	CST	9520	100
		IF	pSMAD2/3	Rabbit monoclonal	D27F4	CST	8828S	200
		IF	ZEB1	Rabbit monoclonal	D80D3	CST	3396	200
		IF	Snail	Rabbit monoclonal	C15D3	CST	3879	200
	**c**	IB	Twist	Mouse monoclonal	2C1a	Abcam	ab50887	50
		IB	Snail	Rabbit monoclonal	C15D3	CST	3879	500
		IB	ZEB1	Rabbit monoclonal	D80D3	CST	3396	1000
		IB	Lamin-B1	Rabbit polyclonal		Abcam	ab16048	1000
**Figure 3**	**a**	IB	TGFβRI	Rabbit polyclonal	sc-398	SANTA CRUZ	lot B2013	200
		IB	TGFβRII	Rabbit polyclonal	sc-400	SANTA CRUZ	lot H1012	200
		IB	γ-Tublin	Mouse monoclonal	GTU-88	SIGMA	T6557	1000
	**d**	IB	TGFβRI	Rabbit polyclonal	sc-398	SANTA CRUZ	lot B2013	200
		IB	TGFβRII	Rabbit polyclonal	sc-400	SANTA CRUZ	lot H1012	200
		IB	pSMAD2	Rabbit monoclonal	138D4	CST	3108	500
		IB	pSMAD3	Rabbit monoclonal	C25A9	CST	9520	500
		IB	E-cadherin	Mouse monoclonal	36	BD Biosciences	610182	250
		IB	Vimentin	Rabbit monoclonal	D21H3	CST	5741	1000
		IB	N-cadherin	Rabbit monoclonal	D4R1H	CST	13116	1000
		IB	γ-Tublin	Mouse monoclonal	GTU-88	SIGMA	T6557	1000
**Figure 4**	**b, c**	IB	TGFβRI	Rabbit polyclonal	sc-398	SANTA CRUZ	lot B2013	200
		IB	γ-Tublin	Mouse monoclonal	GTU-88	SIGMA	T6557	1000
**Supplementary Figure S4**	**a**	IB	TNIK	Mouse monoclonal	53	BD Biosciences	612250	2000
		IB	pTNIK	Rabbit monoclonal	#35	in-house		200
		IB	Axin1	Rabbit monoclonal	C95H11	CST	2074	1000
		IB	Axin2	Rabbit monoclonal	76G6	CST	2151	500
		IB	cMyc	Rabbit monoclonal	D84C12	cell signaling	1472-1	1000
		IB	γ-Tublin	Mouse monoclonal	GTU-88	SIGMA	T6557	1000
	**b**	IB	CD133	Mouse monoclonal	W6B3C1	Miltenyi Biotec	130-092-395	100
		IB	CD44	Mouse monoclonal	156-3C11	Thermo Fisher Scientific	MS668P0	250
		IB	ALDH1	Rabbit monoclonal	D4R9V	cell signaling	12035	1000
		IB	γ-Tublin	Mouse monoclonal	GTU-88	SIGMA	T6557	1000
**Supplementary Figure S6**		IF	E-cadherin	Mouse monoclonal	36	BD Biosciences	610182	50
		IF	pSMAD2/3	Rabbit monoclonal	D27F4	CST	8828S	200
		IF	ZEB1	Rabbit monoclonal	D80D3	CST	3396	200
		IF	Snail	Rabbit monoclonal	C15D3	CST	3879	200

Abbreviation: IB, immunoblotting; IF, immunofluorescence; BD, Becton & Dickinson; CST, Cell Signaling Technology.

Supplementary Table S2. Real-time PCR primer and probe sets, miRNA mimics, and miRNA inhibitors used in this study.

Figure		Gene Symbol	Species	Resource	Catalog #
**Figure 3**	**b**	*TGFBR1*	human	Applied Byosistems	Hs00610320_m1
		ACTB	human	Applied Byosistems	Hs99999903_m1
	**c, d**	siTβR1 (*TGFBR1*)	human	Applied Byosistems	S14071
		siNC (negative control)	human	Applied Byosistems	AM4611
**Figure 4**	**a**	miR-320a	human	Applied Byosistems	002277
		miR-320b	human	Applied Byosistems	002844
		miR-320d	human	Applied Byosistems	241066
		miR-186-5p	human	Applied Byosistems	002285
		U6 snRNA	human	Applied Byosistems	001973
	**b**	miR-320a mimic	human	Applied Byosistems	MC11621
		miR-320b mimic	human	Applied Byosistems	MC13132
		miR-320d mimic	human	Applied Byosistems	MC13131
		miR-186-5p mimic	human	Applied Byosistems	MC11753
		miR-Ctr (negative control)	human	Applied Byosistems	4464058
	**c**	miR-320a inhibitor	human	Applied Byosistems	MH11621
		miR-320b inhibitor	human	Applied Byosistems	MH13132
		miR-320d inhibitor	human	Applied Byosistems	MH13131
		miR-186-5p inhibitor	human	Applied Byosistems	MH11753
		miR-Ctr (negative control)	human	Applied Byosistems	4464077
**Supplementary Figure S1**		*CDH1*	human	Applied Byosistems	Hs01023895_m1
		CDH2	human	Applied Byosistems	Hs00983056_m1
		VIM	human	Applied Byosistems	Hs00958111_m1
		ACTB	human	Applied Byosistems	Hs99999903_m1

Supplementary Table S3. miRNA microarray data.

Systematic Name	DMSO (Control)	TGFβ1 + DMSO	TGFβ1 + NCB-0846	TGFβ1 + NCB-0970
hsa-let-7a-3p	0.1	0.1	0.1	0.1
hsa-let-7a-5p	840.274	835.795	802.223	908.568
hsa-let-7b-3p	0.1	0.1	0.1	0.1
hsa-let-7b-5p	274.805	253.127	275.279	258.323
hsa-let-7c-3p	0.1	0.1	0.1	0.1
hsa-let-7c-5p	148.225	132.295	151.678	137.077
hsa-let-7d-3p	0.1	0.1	0.1	0.1
hsa-let-7d-5p	64.4341	61.8796	57.1928	66.2934
hsa-let-7e-3p	0.1	0.1	0.1	0.1
hsa-let-7e-5p	157.354	222.668	172.245	268.667
hsa-let-7f-1-3p	0.1	0.1	0.1	0.1
hsa-let-7f-2-3p	0.1	0.1	0.1	0.1
hsa-let-7f-5p	698.544	692.16	645.401	756.955
hsa-let-7g-3p	0.1	0.1	0.1	0.1
hsa-let-7g-5p	163.07	157.289	133.835	179.799
hsa-let-7i-3p	0.1	0.1	0.1	0.1
hsa-let-7i-5p	472.961	522.9	444.36	590.277
hsa-miR-100-3p	0.1	0.1	0.1	0.1
hsa-miR-100-5p	107.448	191.276	105.525	250.087
hsa-miR-101-3p	10.1981	11.1553	2.4956	14.9508
hsa-miR-101-5p	0.1	0.1	0.1	0.1
hsa-miR-103a-2-5p	0.1	0.1	0.1	0.1
hsa-miR-103a-3p	136.754	125.829	111.085	180.334
hsa-miR-103b	0.1	0.1	0.1	0.1
hsa-miR-105-3p	0.1	0.1	0.1	0.1
hsa-miR-105-5p	0.1	0.1	0.1	0.1
hsa-miR-106a-3p	0.1	0.1	0.1	0.1
hsa-miR-106b-3p	0.1	0.1	0.1	0.1
hsa-miR-106b-5p	102.111	96.0757	76.8515	134.696
hsa-miR-107	146.083	133.784	139.001	187.576
hsa-miR-10a-3p	0.1	0.1	0.1	0.1
hsa-miR-10a-5p	25.1412	19.19	19.7574	17.5567
hsa-miR-10b-3p	0.1	0.1	0.1	0.1
hsa-miR-10b-5p	8.99454	7.64448	6.81585	7.02133
hsa-miR-1178-3p	0.1	0.1	0.1	0.1
hsa-miR-1178-5p	0.1	0.1	0.1	0.1
hsa-miR-1179	0.1	0.1	0.1	0.1
hsa-miR-1180-3p	0.1	0.1	0.1	0.1
hsa-miR-1180-5p	0.1	0.1	0.1	0.1
hsa-miR-1181	4.08338	3.64703	7.04148	0.1
hsa-miR-1182	0.1	0.1	0.1	0.1
hsa-miR-1183	0.1	0.1	0.1	0.1
hsa-miR-1184	0.1	0.1	0.1	0.1
hsa-miR-1185-1-3p	1.59198	0.1	2.05331	0.1
hsa-miR-1185-2-3p	0.1	0.1	0.1	0.1
hsa-miR-1185-5p	0.1	0.1	0.1	0.1
hsa-miR-1193	0.1	0.1	0.1	0.1
hsa-miR-1197	0.1	0.1	0.1	0.1
hsa-miR-1199-3p	0.1	0.1	0.1	0.1
hsa-miR-1199-5p	0.1	0.1	0.1	0.1

hsa-miR-1200	0.1	0.1	0.1	0.1
hsa-miR-1202	20.54	27.0059	36.3362	29.6413
hsa-miR-1203	0.1	0.1	0.1	0.1
hsa-miR-1204	0.1	0.1	0.1	0.1
hsa-miR-1205	0.1	0.1	0.1	0.1
hsa-miR-1206	0.1	0.1	0.1	0.1
hsa-miR-1207-3p	0.1	2.23204	0.1	2.15523
hsa-miR-1207-5p	45.7403	37.7841	55.4974	28.318
hsa-miR-1208	0.1	0.1	0.1	0.1
hsa-miR-122-3p	0.1	0.1	0.1	0.1
hsa-miR-1224-3p	0.1	0.1	0.1	0.1
hsa-miR-1224-5p	0.1	0.1	2.11655	0.1
hsa-miR-1225-5p	43.2725	35.7447	52.5087	28.3145
hsa-miR-122-5p	0.1	0.1	0.1	0.1
hsa-miR-1226-3p	0.1	0.1	0.1	0.1
hsa-miR-1226-5p	0.1	0.1	0.1	0.1
hsa-miR-1227-3p	0.1	0.1	0.1	0.1
hsa-miR-1227-5p	6.04207	5.93852	7.76467	2.39279
hsa-miR-1228-3p	0.1	0.1	0.1	0.1
hsa-miR-1228-5p	0.1	0.1	0.1	0.1
hsa-miR-1229-3p	0.1	0.1	0.1	0.1
hsa-miR-1229-5p	5.90695	6.24197	7.97529	3.52077
hsa-miR-1231	0.1	0.1	0.1	0.1
hsa-miR-1233-3p	0.1	0.1	0.1	0.1
hsa-miR-1233-5p	0.1	0.1	0.1	0.1
hsa-miR-1234-3p	0.1	0.1	0.1	0.1
hsa-miR-1236-3p	0.1	0.1	0.1	0.1
hsa-miR-1236-5p	0.1	0.1	0.1	0.1
hsa-miR-1237-3p	0.1	0.1	0.1	0.1
hsa-miR-1237-5p	0.1	0.1	0.1	0.1
hsa-miR-1238-3p	0.1	0.1	0.1	0.1
hsa-miR-1238-5p	0.1	0.1	0.1	0.1
hsa-miR-1243	0.1	0.1	0.1	0.1
hsa-miR-124-3p	0.1	0.1	0.1	0.1
hsa-miR-1244	0.1	0.1	0.1	0.1
hsa-miR-1245a	0.1	0.1	0.1	0.1
hsa-miR-1245b-3p	0.1	0.1	0.1	0.1
hsa-miR-1245b-5p	0.1	0.1	0.1	0.1
hsa-miR-124-5p	0.1	0.1	0.1	0.1
hsa-miR-1246	12.9665	47.5727	130.001	33.4813
hsa-miR-1247-3p	0.1	0.1	0.1	0.1
hsa-miR-1247-5p	0.1	0.1	0.1	0.1
hsa-miR-1248	0.1	0.1	0.1	0.1
hsa-miR-1249-3p	0.1	0.1	0.1	0.1
hsa-miR-1249-5p	2.45674	2.26772	2.31613	1.64706
hsa-miR-1250-3p	0.1	0.1	0.1	0.1
hsa-miR-1250-5p	0.1	0.1	0.1	0.1
hsa-miR-1251-3p	0.1	0.1	0.1	0.1
hsa-miR-1251-5p	0.1	0.1	0.1	0.1
hsa-miR-1252-3p	0.1	0.1	0.1	0.1
hsa-miR-1252-5p	0.1	0.1	0.1	0.1
hsa-miR-1253	0.1	0.1	0.1	0.1
hsa-miR-1254	0.1	0.1	0.1	0.1

hsa-miR-1255a	0.1	0.1	0.1	0.1
hsa-miR-1255b-2-3p	0.1	0.1	0.1	0.1
hsa-miR-1255b-5p	0.1	0.1	0.1	0.1
hsa-miR-1256	0.1	0.1	0.1	0.1
hsa-miR-1257	0.1	0.1	0.1	0.1
hsa-miR-1258	0.1	0.1	0.1	0.1
hsa-miR-125a-3p	5.4593	6.06916	3.71725	4.97975
hsa-miR-125a-5p	54.8335	77.9028	19.4877	86.4494
hsa-miR-125b-1-3p	0.1	0.1	0.1	0.1
hsa-miR-125b-2-3p	0.1	0.1	0.1	0.1
hsa-miR-125b-5p	89.3087	155.108	77.2147	181.402
hsa-miR-1260a	89.4479	241.867	125.303	330.356
hsa-miR-1260b	116.006	264.886	150.387	381.086
hsa-miR-1261	0.1	0.1	0.1	0.1
hsa-miR-1262	0.1	0.1	0.1	0.1
hsa-miR-1263	0.1	0.1	0.1	0.1
hsa-miR-126-3p	8.51213	7.84399	6.36112	8.36438
hsa-miR-1264	0.1	0.1	0.1	0.1
hsa-miR-1265	0.1	0.1	0.1	0.1
hsa-miR-126-5p	0.1	0.1	0.1	0.1
hsa-miR-1266-3p	0.1	0.1	0.1	0.1
hsa-miR-1266-5p	0.1	0.1	0.1	0.1
hsa-miR-1267	0.1	0.1	0.1	0.1
hsa-miR-1268a	6.91557	11.1827	51.7512	7.88996
hsa-miR-1268b	0.1	0.1	0.1	0.1
hsa-miR-1269a	0.1	0.1	0.1	0.1
hsa-miR-1269b	0.1	0.1	0.1	0.1
hsa-miR-1270	0.1	0.1	0.1	0.1
hsa-miR-1271-3p	0.1	0.1	0.1	0.1
hsa-miR-1271-5p	0.1	0.1	0.1	0.1
hsa-miR-1272	0.1	0.1	0.1	0.1
hsa-miR-1273a	0.1	0.1	0.1	0.1
hsa-miR-1273c	0.1	0.1	0.1	0.1
hsa-miR-1273d	0.1	0.1	0.1	0.1
hsa-miR-1273e	0.1	0.1	0.1	0.1
hsa-miR-1273f	0.1	0.1	3.72478	0.1
hsa-miR-1273g-3p	698.203	765.186	1292.65	1003.48
hsa-miR-1273g-5p	0.1	0.1	0.1	0.1
hsa-miR-1273h-3p	0.1	0.1	0.1	0.1
hsa-miR-1273h-5p	0.1	0.1	0.1	0.1
hsa-miR-127-3p	0.1	0.1	0.1	0.1
hsa-miR-1275	12.3334	12.133	10.8777	8.37309
hsa-miR-127-5p	0.1	0.1	0.1	0.1
hsa-miR-1276	0.1	0.1	0.1	0.1
hsa-miR-1277-3p	0.1	0.1	0.1	0.1
hsa-miR-1278	0.1	0.1	0.1	0.1
hsa-miR-1279	0.1	0.1	0.1	0.1
hsa-miR-1281	0.1	0.1	0.1	0.1
hsa-miR-128-1-5p	0.1	0.1	0.1	0.1
hsa-miR-1282	0.1	0.1	0.1	0.1
hsa-miR-128-2-5p	0.1	0.1	0.1	0.1
hsa-miR-1283	0.1	0.1	0.1	0.1
hsa-miR-128-3p	11.0671	8.20704	8.69734	10.4719

hsa-miR-1284	0.1	0.1	0.1	0.1
hsa-miR-1285-3p	0.1	0.1	0.1	0.1
hsa-miR-1285-5p	0.1	0.1	0.1	0.1
hsa-miR-1286	0.1	0.1	0.1	0.1
hsa-miR-1287-3p	0.1	0.1	0.1	0.1
hsa-miR-1287-5p	0.1	0.1	6.32102	1.69999
hsa-miR-1288-3p	1.5505	0.1	0.1	1.76478
hsa-miR-1288-5p	0.1	0.1	0.1	0.1
hsa-miR-1289	0.1	0.1	0.1	0.1
hsa-miR-1290	2.17096	6.00376	28.0232	5.86481
hsa-miR-1291	0.1	0.1	0.1	0.1
hsa-miR-129-1-3p	0.1	0.1	0.1	1.8685
hsa-miR-1292-3p	0.1	0.1	0.1	0.1
hsa-miR-129-2-3p	0.1	0.1	0.1	1.67454
hsa-miR-1292-5p	0.1	0.1	0.1	0.1
hsa-miR-1293	0.1	0.1	0.1	0.1
hsa-miR-1294	0.1	0.1	0.1	0.1
hsa-miR-1295a	0.1	0.1	0.1	0.1
hsa-miR-1295b-3p	0.1	0.1	0.1	0.1
hsa-miR-1295b-5p	0.1	0.1	0.1	0.1
hsa-miR-129-5p	0.1	0.1	0.1	0.1
hsa-miR-1296-3p	0.1	0.1	0.1	0.1
hsa-miR-1296-5p	0.1	0.1	0.1	0.1
hsa-miR-1297	0.1	0.1	0.1	0.1
hsa-miR-1298-3p	0.1	0.1	0.1	0.1
hsa-miR-1298-5p	0.1	0.1	0.1	0.1
hsa-miR-1299	0.1	0.1	0.1	0.1
hsa-miR-1301-3p	0.1	0.1	0.1	0.1
hsa-miR-1301-5p	0.1	0.1	0.1	0.1
hsa-miR-1302	0.1	0.1	0.1	0.1
hsa-miR-1303	0.1	0.1	0.1	0.1
hsa-miR-1304-3p	0.1	0.1	0.1	0.1
hsa-miR-1304-5p	0.1	0.1	0.1	0.1
hsa-miR-1305	9.63932	9.25758	9.18199	11.0083
hsa-miR-1306-3p	0.1	0.1	0.1	0.1
hsa-miR-1306-5p	0.1	0.1	0.1	0.1
hsa-miR-1307-3p	0.1	0.1	0.1	0.1
hsa-miR-1307-5p	0.1	0.1	0.1	0.1
hsa-miR-130a-3p	116.018	125.131	105.44	132.997
hsa-miR-130a-5p	0.1	0.1	0.1	0.1
hsa-miR-130b-3p	10.6947	15.5106	7.98623	16.7573
hsa-miR-130b-5p	0.1	0.1	0.1	0.1
hsa-miR-1321	0.1	0.1	0.1	0.1
hsa-miR-1322	0.1	0.1	0.1	0.1
hsa-miR-1323	0.1	0.1	0.1	0.1
hsa-miR-132-3p	0.1	0.1	12.5157	2.13265
hsa-miR-1324	0.1	0.1	0.1	0.1
hsa-miR-132-5p	0.1	0.1	0.1	0.1
hsa-miR-133a-3p	0.1	0.1	0.1	0.1
hsa-miR-133a-5p	0.1	0.1	0.1	0.1
hsa-miR-133b	0.1	0.1	0.1	0.1
hsa-miR-1343-3p	0.1	0.1	0.1	0.1
hsa-miR-1343-5p	0.1	0.1	0.1	0.1

hsa-miR-134-3p	0.1	0.1	0.1	0.1
hsa-miR-134-5p	10.7655	8.50413	14.7789	5.74026
hsa-miR-135a-3p	0.1	0.1	0.1	0.1
hsa-miR-135a-5p	0.1	0.1	0.1	0.1
hsa-miR-135b-3p	0.1	0.1	0.1	0.1
hsa-miR-135b-5p	0.1	4.5114	0.1	4.62761
hsa-miR-136-3p	0.1	0.1	0.1	0.1
hsa-miR-136-5p	0.1	0.1	0.1	0.1
hsa-miR-137	3.39783	5.78683	0.1	8.91231
hsa-miR-138-1-3p	0.1	0.1	0.1	0.1
hsa-miR-138-2-3p	0.1	0.1	0.1	0.1
hsa-miR-138-5p	5.69316	2.37111	10.9933	3.15206
hsa-miR-139-3p	0.1	0.1	0.1	0.1
hsa-miR-139-5p	0.1	0.1	0.1	0.1
hsa-miR-1-3p	0.1	0.1	0.1	0.1
hsa-miR-140-3p	10.1487	8.19456	8.47767	8.31451
hsa-miR-140-5p	10.9719	9.21798	6.25013	9.68916
hsa-miR-141-3p	0.1	0.1	0.1	0.1
hsa-miR-141-5p	0.1	0.1	0.1	0.1
hsa-miR-142-3p	0.1	0.1	0.1	0.1
hsa-miR-142-5p	0.1	0.1	0.1	0.1
hsa-miR-143-3p	0.1	0.1	0.1	0.1
hsa-miR-143-5p	0.1	0.1	0.1	0.1
hsa-miR-144-3p	0.1	0.1	0.1	0.1
hsa-miR-144-5p	0.1	0.1	0.1	0.1
hsa-miR-145-3p	0.1	0.1	0.1	0.1
hsa-miR-145-5p	0.1	0.1	0.1	0.1
hsa-miR-1468-3p	0.1	0.1	0.1	0.1
hsa-miR-1468-5p	0.1	0.1	0.1	0.1
hsa-miR-1469	0.1	0.1	0.1	0.1
hsa-miR-146a-3p	0.1	0.1	0.1	0.1
hsa-miR-146a-5p	0.1	0.1	0.1	0.1
hsa-miR-146b-3p	0.1	0.1	0.1	0.1
hsa-miR-146b-5p	0.1	0.1	0.1	0.1
hsa-miR-1470	0.1	0.1	0.1	0.1
hsa-miR-1471	0.1	0.1	0.1	0.1
hsa-miR-147a	0.1	0.1	0.1	0.1
hsa-miR-147b	0.1	0.1	0.1	0.1
hsa-miR-148a-3p	5.67374	0.1	0.1	0.1
hsa-miR-148a-5p	0.1	0.1	0.1	0.1
hsa-miR-148b-3p	10.2598	7.32114	5.69306	11.435
hsa-miR-148b-5p	0.1	0.1	0.1	0.1
hsa-miR-149-3p	0.1	0.1	0.1	0.1
hsa-miR-149-5p	2.27288	2.10626	0.1	2.34681
hsa-miR-150-3p	1.1488	1.54922	8.58649	0.990472
hsa-miR-150-5p	0.1	0.1	0.1	0.1
hsa-miR-151a-3p	16.5823	18.9815	14.4579	22.8759
hsa-miR-151a-5p	59.2107	64.2104	72.3252	81.5449
hsa-miR-151b	29.199	33.7053	31.5282	39.8117
hsa-miR-152-3p	0.1	0.1	0.1	0.1
hsa-miR-152-5p	0.1	0.1	0.1	0.1
hsa-miR-153-3p	0.1	0.1	0.1	0.1
hsa-miR-153-5p	0.1	0.1	0.1	0.1

hsa-miR-1537-3p	0.1	0.1	0.1	0.1
hsa-miR-1537-5p	0.1	0.1	0.1	0.1
hsa-miR-1538	0.1	0.1	0.1	0.1
hsa-miR-1539	0.1	0.1	0.1	0.1
hsa-miR-154-3p	0.1	0.1	0.1	1.92458
hsa-miR-154-5p	0.1	0.1	0.1	0.1
hsa-miR-155-3p	0.1	0.1	0.1	0.1
hsa-miR-155-5p	0.1	0.1	0.1	0.1
hsa-miR-1587	8.76698	9.62748	12.3398	7.84752
hsa-miR-15a-3p	0.1	0.1	0.1	0.1
hsa-miR-15a-5p	56.6189	54.3719	48.9565	68.3459
hsa-miR-15b-3p	0.790755	1.10143	0.1	1.27457
hsa-miR-15b-5p	321.584	313.987	247.91	357.466
hsa-miR-1-5p	0.1	0.1	0.1	0.1
hsa-miR-16-1-3p	0.1	0.1	0.1	0.1
hsa-miR-16-2-3p	2.33823	3.28391	1.99633	2.5927
hsa-miR-16-5p	466.566	481.523	406.755	510.086
hsa-miR-17-3p	3.20849	3.98437	0.1	2.56696
hsa-miR-17-5p	97.5544	97.1912	61.7061	83.729
hsa-miR-181a-2-3p	0.1	2.36484	0.1	2.74539
hsa-miR-181a-3p	0.1	0.1	0.1	1.74865
hsa-miR-181a-5p	15.2733	40.1894	28.4765	44.1969
hsa-miR-181b-2-3p	0.1	0.1	0.1	0.1
hsa-miR-181b-3p	0.1	0.1	0.1	0.1
hsa-miR-181b-5p	6.16952	18.3422	11.72	23.0121
hsa-miR-181c-3p	0.1	0.1	0.1	0.1
hsa-miR-181c-5p	0.1	0.1	0.1	0.1
hsa-miR-181d-3p	0.1	0.1	0.1	0.1
hsa-miR-181d-5p	0.1	2.05482	0.1	2.7405
hsa-miR-182-3p	0.1	0.1	0.1	0.1
hsa-miR-1825	0.1	0.1	0.1	0.1
hsa-miR-182-5p	3.02022	4.29087	2.02042	4.89969
hsa-miR-1827	0.1	0.1	0.1	0.1
hsa-miR-183-3p	0.1	0.1	0.1	0.1
hsa-miR-183-5p	2.17779	2.40535	2.90691	4.34678
hsa-miR-184	0.1	0.1	0.1	0.1
hsa-miR-185-3p	0.1	0.1	0.1	0.1
hsa-miR-185-5p	7.51614	7.8449	5.54607	9.78651
hsa-miR-186-3p	0.1	0.1	0.1	0.1
hsa-miR-186-5p	1.87782	0.1	3.18453	2.20567
hsa-miR-187-3p	0.1	0.1	0.1	0.1
hsa-miR-187-5p	0.1	0.1	0.1	0.1
hsa-miR-188-3p	0.1	0.1	0.1	0.1
hsa-miR-188-5p	3.73347	0.1	5.58633	0.1
hsa-miR-18a-3p	0.1	0.1	0.1	0.1
hsa-miR-18a-5p	10.9753	12.1498	2.35792	8.67464
hsa-miR-18b-3p	0.1	0.1	0.1	0.1
hsa-miR-18b-5p	2.92445	2.98795	1.22156	2.37744
hsa-miR-1908-3p	0.1	0.1	0.1	0.1
hsa-miR-1908-5p	0.1	0.1	0.1	0.1
hsa-miR-1909-3p	0.1	0.1	0.1	0.1
hsa-miR-1909-5p	0.1	0.1	0.1	0.1
hsa-miR-190a-3p	0.1	0.1	0.1	0.1

hsa-miR-190a-5p	0.1	0.1	0.1	0.1
hsa-miR-190b	0.1	0.1	0.1	0.1
hsa-miR-1910-3p	0.1	0.1	0.1	0.1
hsa-miR-1910-5p	0.1	0.1	0.1	0.1
hsa-miR-1911-3p	0.1	0.1	0.1	0.1
hsa-miR-1911-5p	0.1	0.1	0.1	0.1
hsa-miR-1912	0.1	0.1	0.1	0.1
hsa-miR-1913	0.1	0.1	0.1	0.1
hsa-miR-191-3p	0.1	0.1	0.1	0.1
hsa-miR-1914-3p	3.13398	0.1	1.9375	1.82469
hsa-miR-1914-5p	0.1	0.1	0.1	0.1
hsa-miR-1915-3p	25.659	23.775	25.9002	15.1081
hsa-miR-1915-5p	0.1	0.1	0.1	0.1
hsa-miR-191-5p	0.1	0.1	0.1	0.1
hsa-miR-192-3p	3.79074	1.75367	2.0373	2.14499
hsa-miR-192-5p	328.163	173.349	268.686	227.28
hsa-miR-193a-3p	3.93681	8.20868	3.62876	7.95082
hsa-miR-193a-5p	0.1	0.1	0.1	0.1
hsa-miR-193b-3p	129.223	263.916	153.311	267.915
hsa-miR-193b-5p	0.1	2.71474	0.1	1.9467
hsa-miR-194-3p	0.1	0.1	0.1	0.1
hsa-miR-194-5p	139.266	86.3635	119.511	117.972
hsa-miR-195-3p	0.1	0.1	0.1	0.1
hsa-miR-195-5p	2.61628	2.93218	2.74647	2.44582
hsa-miR-196a-3p	0.1	0.1	0.1	0.1
hsa-miR-196a-5p	6.36498	5.88468	3.31587	7.37517
hsa-miR-196b-3p	0.1	0.1	0.1	0.1
hsa-miR-196b-5p	2.5107	2.56799	2.22135	3.04566
hsa-miR-1972	0.1	0.1	3.76156	0.1
hsa-miR-1973	34.2023	27.6989	35.4183	29.2851
hsa-miR-197-3p	2.13218	2.22202	2.15819	1.99049
hsa-miR-197-5p	27.9701	28.673	30.2469	26.0978
hsa-miR-1976	0.1	0.1	0.1	0.1
hsa-miR-198	0.1	0.1	0.1	0.1
hsa-miR-199a-3p	0.1	0.1	0.1	0.1
hsa-miR-199a-5p	0.1	0.1	0.1	0.1
hsa-miR-199b-5p	0.1	0.1	0.1	0.1
hsa-miR-19a-3p	53.2501	65.7195	16.2307	58.6204
hsa-miR-19a-5p	0.1	0.1	0.1	0.1
hsa-miR-19b-1-5p	0.1	0.1	0.1	0.1
hsa-miR-19b-2-5p	0.1	0.1	0.1	0.1
hsa-miR-19b-3p	205.89	225.405	71.7765	197.399
hsa-miR-200a-3p	0.1	0.1	0.1	1.89373
hsa-miR-200a-5p	0.1	0.1	0.1	0.1
hsa-miR-200b-3p	2.065	6.45235	1.97192	6.60081
hsa-miR-200b-5p	0.1	0.1	0.1	0.1
hsa-miR-200c-3p	0.1	0.1	0.1	0.1
hsa-miR-200c-5p	0.1	0.1	0.1	0.1
hsa-miR-202-3p	0.1	0.1	0.1	0.1
hsa-miR-202-5p	0.1	0.1	0.1	0.1
hsa-miR-203a-3p	0.1	0.1	0.1	0.1
hsa-miR-203a-5p	0.1	0.1	0.1	0.1
hsa-miR-203b-3p	0.1	0.1	0.1	0.1

hsa-miR-203b-5p	0.1	0.1	0.1	0.1
hsa-miR-204-3p	0.1	0.1	0.1	0.1
hsa-miR-204-5p	0.1	0.1	0.1	0.1
hsa-miR-2052	0.1	0.1	0.1	0.1
hsa-miR-2053	0.1	0.1	0.1	0.1
hsa-miR-205-3p	0.1	0.1	0.1	0.1
hsa-miR-205-5p	0.1	0.1	0.1	0.1
hsa-miR-206	0.1	0.1	0.1	0.1
hsa-miR-208a-3p	0.1	0.1	0.1	0.1
hsa-miR-208a-5p	0.1	0.1	0.1	0.1
hsa-miR-208b-3p	0.1	0.1	0.1	0.1
hsa-miR-208b-5p	0.1	0.1	0.1	0.1
hsa-miR-20a-3p	0.1	1.88671	0.1	0.1
hsa-miR-20a-5p	185.537	195.419	131.377	156.317
hsa-miR-20b-3p	0.1	0.1	0.1	0.1
hsa-miR-20b-5p	52.1499	56.7959	37.4412	44.9273
hsa-miR-210-3p	16.7008	19.8483	8.41841	20.7808
hsa-miR-210-5p	0.1	0.1	0.1	0.1
hsa-miR-2110	0.1	0.1	0.1	0.1
hsa-miR-2113	0.1	0.1	0.1	0.1
hsa-miR-211-3p	0.1	0.1	0.1	0.1
hsa-miR-2114-3p	0.1	0.1	0.1	0.1
hsa-miR-2114-5p	0.1	0.1	0.1	0.1
hsa-miR-2115-3p	0.1	0.1	0.1	0.1
hsa-miR-2115-5p	0.1	0.1	0.1	0.1
hsa-miR-211-5p	0.1	0.1	0.1	0.1
hsa-miR-2116-3p	0.1	0.1	0.1	0.1
hsa-miR-2116-5p	0.1	0.1	0.1	0.1
hsa-miR-2117	0.1	0.1	0.1	0.1
hsa-miR-212-3p	0.1	0.1	0.1	0.1
hsa-miR-212-5p	0.1	0.1	0.1	0.1
hsa-miR-21-3p	30.251	61.6796	4.13878	34.8386
hsa-miR-214-3p	0.1	0.1	0.1	0.1
hsa-miR-214-5p	0.1	0.1	0.1	0.1
hsa-miR-215-3p	0.1	0.1	0.1	0.1
hsa-miR-215-5p	221.818	115.907	182.286	155.219
hsa-miR-21-5p	6615.52	8331.83	4902.56	8788.46
hsa-miR-216a-3p	0.1	0.1	0.1	0.1
hsa-miR-216a-5p	0.1	0.1	0.1	0.1
hsa-miR-216b-3p	0.1	0.1	0.1	0.1
hsa-miR-216b-5p	0.1	0.1	0.1	0.1
hsa-miR-217	0.1	0.1	0.1	0.1
hsa-miR-218-1-3p	0.1	0.1	0.1	0.1
hsa-miR-218-2-3p	0.1	0.1	0.1	0.1
hsa-miR-218-5p	1.63166	7.02087	0.1	6.81268
hsa-miR-219a-1-3p	0.1	0.1	0.1	0.1
hsa-miR-219a-2-3p	0.1	0.1	0.1	0.1
hsa-miR-219a-5p	0.1	0.1	0.1	0.1
hsa-miR-219b-3p	0.1	0.1	0.1	0.1
hsa-miR-219b-5p	0.1	0.1	0.1	0.1
hsa-miR-221-3p	40.3524	33.3154	36.7892	42.5429
hsa-miR-221-5p	0.1	0.1	0.1	1.77584
hsa-miR-222-3p	17.6726	11.2002	32.2092	14.0242

hsa-miR-222-5p	0.1	0.1	0.1	0.1
hsa-miR-223-3p	0.1	0.1	0.1	0.1
hsa-miR-223-5p	0.1	0.1	0.1	0.1
hsa-miR-22-3p	130.916	199.878	109.131	222.804
hsa-miR-224-3p	6.52484	3.01983	5.1502	5.30981
hsa-miR-224-5p	156.602	101.511	157.34	119.401
hsa-miR-22-5p	3.1984	5.66334	0.1	4.68756
hsa-miR-2276-3p	0.1	0.1	0.1	0.1
hsa-miR-2276-5p	0.1	0.1	0.1	0.1
hsa-miR-2277-3p	0.1	0.1	0.1	0.1
hsa-miR-2277-5p	0.1	0.1	0.1	0.1
hsa-miR-2278	0.1	0.1	0.1	0.1
hsa-miR-2355-3p	0.1	0.1	0.1	0.1
hsa-miR-2355-5p	0.1	0.1	0.1	0.1
hsa-miR-2392	0.1	0.1	0.1	0.1
hsa-miR-23a-3p	394.126	825.429	463.932	927.99
hsa-miR-23a-5p	0.1	0.1	0.1	0.1
hsa-miR-23b-3p	299.862	221.302	324.003	279.889
hsa-miR-23b-5p	0.1	0.1	0.1	0.1
hsa-miR-23c	0.1	0.1	0.1	0.1
hsa-miR-24-1-5p	0.1	0.1	0.1	0.1
hsa-miR-24-2-5p	0.1	0.1	0.1	0.1
hsa-miR-24-3p	287.439	406.282	249.984	483.559
hsa-miR-2467-3p	0.1	0.1	0.1	0.1
hsa-miR-2467-5p	0.1	0.1	0.1	0.1
hsa-miR-25-3p	88.2441	86.9416	69.6381	91.3267
hsa-miR-25-5p	0.1	0.1	0.1	0.1
hsa-miR-2681-3p	0.1	0.1	0.1	0.1
hsa-miR-2681-5p	0.1	0.1	0.1	0.1
hsa-miR-2682-3p	0.1	0.1	0.1	0.1
hsa-miR-2682-5p	0.1	0.1	0.1	0.1
hsa-miR-26a-1-3p	0.1	0.1	0.1	0.1
hsa-miR-26a-2-3p	0.1	0.1	0.1	0.1
hsa-miR-26a-5p	57.4323	60.6612	49.759	54.6455
hsa-miR-26b-3p	0.1	0.1	0.1	0.1
hsa-miR-26b-5p	86.9928	72.4115	72.195	79.6017
hsa-miR-27a-3p	218.098	509.619	163.317	503.194
hsa-miR-27a-5p	0.1	0.1	0.1	0.1
hsa-miR-27b-3p	359.971	278.554	281.264	333.293
hsa-miR-27b-5p	0.1	0.1	0.1	0.1
hsa-miR-28-3p	0.1	0.1	0.1	0.1
hsa-miR-28-5p	7.95081	7.7159	8.57142	9.39147
hsa-miR-2861	17.788	19.4794	23.8102	14.9938
hsa-miR-2909	0.1	0.1	0.1	0.1
hsa-miR-296-3p	0.1	0.1	0.1	0.1
hsa-miR-296-5p	0.1	0.1	0.1	0.1
hsa-miR-297	0.1	0.1	0.1	0.1
hsa-miR-298	0.1	0.1	0.1	0.1
hsa-miR-299-3p	0.1	0.1	0.1	0.1
hsa-miR-299-5p	0.1	0.1	0.1	0.1
hsa-miR-29a-3p	534.378	546.386	383.653	650.999
hsa-miR-29a-5p	0.1	0.1	0.1	0.1
hsa-miR-29b-1-5p	6.13247	7.38601	0.1	4.02032

hsa-miR-29b-2-5p	0.1	0.1	0.1	0.1
hsa-miR-29b-3p	236.945	268.924	195.603	366.579
hsa-miR-29c-3p	164.523	143.844	96.171	160.225
hsa-miR-29c-5p	0.1	0.1	0.1	0.1
hsa-miR-300	0.1	0.1	0.1	0.1
hsa-miR-301a-3p	5.78439	7.22344	2.86061	11.153
hsa-miR-301a-5p	0.1	0.1	0.1	0.1
hsa-miR-301b-3p	0.1	0.1	0.1	0.1
hsa-miR-301b-5p	0.1	0.1	0.1	0.1
hsa-miR-302a-3p	0.1	0.1	0.1	0.1
hsa-miR-302a-5p	0.1	0.1	0.1	0.1
hsa-miR-302b-3p	0.1	0.1	0.1	0.1
hsa-miR-302b-5p	0.1	0.1	0.1	0.1
hsa-miR-302c-3p	0.1	0.1	0.1	0.1
hsa-miR-302c-5p	0.1	0.1	0.1	0.1
hsa-miR-302d-3p	0.1	0.1	0.1	0.1
hsa-miR-302d-5p	0.1	0.1	0.1	0.1
hsa-miR-302e	0.1	0.1	0.1	0.1
hsa-miR-302f	0.1	0.1	0.1	0.1
hsa-miR-3064-3p	0.1	0.1	0.1	0.1
hsa-miR-3064-5p	0.1	0.1	0.1	0.1
hsa-miR-3065-3p	0.1	0.1	0.1	0.1
hsa-miR-3065-5p	0.1	0.1	0.1	0.1
hsa-miR-3074-3p	0.1	0.1	0.1	0.1
hsa-miR-3074-5p	0.1	0.1	0.1	0.1
hsa-miR-30a-3p	12.9178	14.4282	11.2442	12.3415
hsa-miR-30a-5p	100.395	100.155	112.417	103.018
hsa-miR-30b-3p	0.1	0.1	0.1	0.1
hsa-miR-30b-5p	73.9505	51.3855	54.4978	56.7747
hsa-miR-30c-1-3p	0.1	0.1	0.1	0.1
hsa-miR-30c-2-3p	0.1	0.1	0.1	0.1
hsa-miR-30c-5p	35.6957	29.2843	39.6078	28.5233
hsa-miR-30d-3p	0.1	0.1	0.1	0.1
hsa-miR-30d-5p	26.8696	23.2426	30.358	27.8272
hsa-miR-30e-3p	5.62847	5.93406	3.34636	4.37713
hsa-miR-30e-5p	15.3284	18.6238	12.8286	19.7245
hsa-miR-3115	0.1	0.1	0.1	0.1
hsa-miR-3116	0.1	0.1	0.1	0.1
hsa-miR-3117-3p	0.1	0.1	0.1	0.1
hsa-miR-3117-5p	0.1	0.1	0.1	0.1
hsa-miR-3118	0.1	0.1	0.1	0.1
hsa-miR-3119	0.1	0.1	0.1	0.1
hsa-miR-3120-3p	0.1	0.1	0.1	0.1
hsa-miR-3120-5p	0.1	0.1	0.1	0.1
hsa-miR-3121-3p	0.1	0.1	0.1	0.1
hsa-miR-3121-5p	0.1	0.1	0.1	0.1
hsa-miR-3122	0.1	0.1	0.1	0.1
hsa-miR-3123	0.1	0.1	0.1	0.1
hsa-miR-3124-3p	0.1	0.1	0.1	0.1
hsa-miR-3124-5p	0.1	0.1	0.1	0.1
hsa-miR-3125	0.1	0.1	0.1	0.1
hsa-miR-3126-3p	0.1	0.1	0.1	0.1
hsa-miR-3126-5p	0.1	0.1	0.1	0.1

hsa-miR-3127-3p	0.1	0.1	0.1	0.1
hsa-miR-3127-5p	0.1	0.1	0.1	0.1
hsa-miR-3128	0.1	0.1	0.1	0.1
hsa-miR-3129-3p	0.1	0.1	0.1	0.1
hsa-miR-3129-5p	0.1	0.1	0.1	0.1
hsa-miR-3130-3p	0.1	0.1	0.1	0.1
hsa-miR-3130-5p	0.1	0.1	0.1	0.1
hsa-miR-3131	0.1	0.1	0.1	0.1
hsa-miR-3132	0.1	0.1	0.1	0.1
hsa-miR-3133	0.1	0.1	0.1	0.1
hsa-miR-3134	0.1	0.1	0.1	0.1
hsa-miR-3135a	0.1	0.1	0.1	0.1
hsa-miR-3135b	2.7995	3.64069	3.18777	2.82218
hsa-miR-3136-3p	0.1	0.1	0.1	0.1
hsa-miR-3136-5p	0.1	0.1	0.1	0.1
hsa-miR-3137	0.1	0.1	0.1	0.1
hsa-miR-3138	0.1	0.1	0.1	0.1
hsa-miR-3139	0.1	0.1	0.1	0.1
hsa-miR-31-3p	62.3403	191.451	31.4383	177.09
hsa-miR-3140-3p	0.1	0.1	0.1	0.1
hsa-miR-3140-5p	0.1	0.1	0.1	0.1
hsa-miR-3141	0.1	0.1	0.1	0.1
hsa-miR-3142	0.1	0.1	0.1	0.1
hsa-miR-3143	0.1	0.1	0.1	0.1
hsa-miR-3144-3p	0.1	0.1	0.1	0.1
hsa-miR-3144-5p	0.1	0.1	0.1	0.1
hsa-miR-3145-3p	0.1	0.1	0.1	0.1
hsa-miR-3145-5p	0.1	0.1	0.1	0.1
hsa-miR-3146	0.1	0.1	0.1	0.1
hsa-miR-3147	0.1	0.1	0.1	0.1
hsa-miR-3148	0.1	0.1	0.1	0.1
hsa-miR-3149	0.1	0.1	0.1	0.1
hsa-miR-3150a-3p	0.1	0.1	0.1	0.1
hsa-miR-3150a-5p	0.1	0.1	0.1	0.1
hsa-miR-3150b-3p	0.1	0.1	0.1	0.1
hsa-miR-3150b-5p	0.1	0.1	0.1	0.1
hsa-miR-3151-3p	0.1	0.1	0.1	0.1
hsa-miR-3151-5p	0.1	0.1	0.1	0.1
hsa-miR-3152-3p	0.1	0.1	0.1	0.1
hsa-miR-3152-5p	0.1	0.1	0.1	0.1
hsa-miR-3153	0.1	0.1	0.1	0.1
hsa-miR-3154	0.1	0.1	0.1	0.1
hsa-miR-3155a	0.1	0.1	0.1	0.1
hsa-miR-3155b	0.1	0.1	0.1	0.1
hsa-miR-3156-3p	0.1	0.1	0.1	0.1
hsa-miR-3156-5p	0.1	0.1	0.1	0.1
hsa-miR-3157-3p	0.1	0.1	0.1	0.1
hsa-miR-3157-5p	0.1	0.1	0.1	0.1
hsa-miR-3158-3p	0.1	0.1	0.1	0.1
hsa-miR-3158-5p	0.1	0.1	0.1	0.1
hsa-miR-3159	0.1	0.1	0.1	0.1
hsa-miR-31-5p	139.986	332.586	153.205	304.068
hsa-miR-3160-3p	0.1	0.1	0.1	0.1

hsa-miR-3160-5p	0.1	0.1	0.1	0.1
hsa-miR-3161	0.1	0.1	0.1	0.1
hsa-miR-3162-3p	0.1	0.1	0.1	0.1
hsa-miR-3162-5p	49.9301	60.0999	65.2393	62.7895
hsa-miR-3163	0.1	0.1	0.1	0.1
hsa-miR-3164	0.1	0.1	0.1	0.1
hsa-miR-3165	0.1	0.1	0.1	0.1
hsa-miR-3166	0.1	0.1	0.1	0.1
hsa-miR-3167	0.1	0.1	0.1	0.1
hsa-miR-3168	0.1	0.1	0.1	0.1
hsa-miR-3169	0.1	0.1	0.1	0.1
hsa-miR-3170	0.1	0.1	0.1	0.1
hsa-miR-3171	0.1	0.1	0.1	0.1
hsa-miR-3173-3p	0.1	0.1	0.1	0.1
hsa-miR-3173-5p	0.1	0.1	0.1	0.1
hsa-miR-3174	0.1	0.1	0.1	0.1
hsa-miR-3175	0.1	0.1	0.1	0.1
hsa-miR-3176	0.1	0.1	0.1	0.1
hsa-miR-3177-3p	0.1	0.1	0.1	0.1
hsa-miR-3177-5p	0.1	0.1	0.1	0.1
hsa-miR-3178	0.1	0.1	0.1	0.1
hsa-miR-3179	0.1	0.1	0.1	0.1
hsa-miR-3180	0.1	0.1	0.1	0.1
hsa-miR-3180-3p	0.1	0.1	0.1	0.1
hsa-miR-3180-5p	0.1	0.1	0.1	0.1
hsa-miR-3181	0.1	0.1	0.1	0.1
hsa-miR-3182	0.1	0.1	0.1	0.1
hsa-miR-3183	0.1	0.1	0.1	0.1
hsa-miR-3184-3p	0.1	0.1	0.1	0.1
hsa-miR-3184-5p	0.1	0.1	0.1	0.1
hsa-miR-3185	0.1	0.1	0.1	0.1
hsa-miR-3186-3p	0.1	0.1	0.1	0.1
hsa-miR-3186-5p	0.1	0.1	0.1	0.1
hsa-miR-3187-3p	0.1	0.1	0.1	0.1
hsa-miR-3187-5p	0.1	0.1	0.1	0.1
hsa-miR-3188	0.1	0.1	0.1	0.1
hsa-miR-3189-3p	0.1	0.1	0.1	0.1
hsa-miR-3189-5p	0.1	0.1	0.1	0.1
hsa-miR-3190-3p	0.1	0.1	0.1	0.1
hsa-miR-3190-5p	0.1	0.1	0.1	0.1
hsa-miR-3191-3p	0.1	0.1	0.1	0.1
hsa-miR-3191-5p	0.1	0.1	0.1	0.1
hsa-miR-3192-3p	0.1	0.1	0.1	0.1
hsa-miR-3192-5p	0.1	0.1	0.1	0.1
hsa-miR-3193	0.1	0.1	0.1	0.1
hsa-miR-3194-3p	0.1	0.1	0.1	0.1
hsa-miR-3194-5p	0.1	0.1	0.1	0.1
hsa-miR-3195	7.26253	10.9607	3.48287	12.2666
hsa-miR-3196	11.4032	12.803	10.4639	10.898
hsa-miR-3197	0.1	0.1	0.1	0.1
hsa-miR-3198	8.34492	8.54268	8.60257	10.0996
hsa-miR-3199	0.1	0.1	0.1	0.1
hsa-miR-3200-3p	0.1	0.1	0.1	0.1

hsa-miR-3200-5p	0.1	0.1	0.1	0.1
hsa-miR-3201	0.1	0.1	0.1	0.1
hsa-miR-3202	0.1	0.1	0.1	0.1
hsa-miR-320a	26.9665	27.9064	73.0187	45.9115
hsa-miR-320b	40.7636	45.8782	103.686	71.4328
hsa-miR-320c	33.205	36.0841	79.9948	51.6107
hsa-miR-320d	46.0511	47.4246	112.26	72.7508
hsa-miR-320e	39.0851	39.3696	94.6616	61.1138
hsa-miR-323a-3p	0.1	0.1	0.1	0.1
hsa-miR-323a-5p	0.1	0.1	0.1	0.1
hsa-miR-323b-3p	0.1	0.1	0.1	0.1
hsa-miR-323b-5p	0.1	0.1	0.1	0.1
hsa-miR-32-3p	0.1	0.1	0.1	0.1
hsa-miR-324-3p	16.6317	19.4317	14.6542	22.6652
hsa-miR-324-5p	10.6694	8.7843	8.73373	13.7577
hsa-miR-325	0.1	0.1	0.1	0.1
hsa-miR-32-5p	0.1	0.1	0.1	0.1
hsa-miR-326	0.1	0.1	0.1	0.1
hsa-miR-328-3p	1.75061	0.1	0.1	1.67107
hsa-miR-328-5p	0.1	0.1	0.1	0.1
hsa-miR-329-3p	0.1	0.1	0.1	0.1
hsa-miR-329-5p	0.1	0.1	0.1	0.1
hsa-miR-330-3p	0.1	0.1	0.1	0.1
hsa-miR-330-5p	0.1	0.1	0.1	0.1
hsa-miR-331-3p	52.5638	52.5663	37.7813	75.4835
hsa-miR-331-5p	0.1	0.1	0.1	0.1
hsa-miR-335-3p	0.1	0.913878	0.1	0.1
hsa-miR-335-5p	3.70328	3.06401	2.10247	3.67026
hsa-miR-337-3p	0.1	0.1	0.1	0.1
hsa-miR-337-5p	0.1	0.1	0.1	0.1
hsa-miR-338-3p	0.1	0.1	0.1	0.1
hsa-miR-338-5p	0.1	0.1	0.1	0.1
hsa-miR-339-3p	0.1	0.1	0.1	0.1
hsa-miR-339-5p	0.1	0.1	0.1	0.1
hsa-miR-33a-3p	0.1	0.1	0.1	0.1
hsa-miR-33a-5p	0.1	0.1	0.1	0.1
hsa-miR-33b-3p	0.1	0.1	0.1	0.1
hsa-miR-33b-5p	0.1	0.1	0.1	0.1
hsa-miR-340-3p	0.1	0.1	0.1	0.1
hsa-miR-340-5p	5.4699	5.41993	0.1	5.09979
hsa-miR-342-3p	8.5294	11.0283	10.4549	15.5051
hsa-miR-342-5p	0.1	0.1	0.1	0.1
hsa-miR-345-3p	0.1	0.1	0.1	0.1
hsa-miR-345-5p	0.1	0.1	0.1	0.1
hsa-miR-346	0.1	0.1	0.1	0.1
hsa-miR-34a-3p	0.1	0.1	0.1	0.1
hsa-miR-34a-5p	94.8349	76.9308	76.1134	100.228
hsa-miR-34b-3p	0.1	0.1	0.1	0.1
hsa-miR-34b-5p	9.89465	8.86202	10.4741	11.3499
hsa-miR-34c-3p	0.1	0.1	0.1	0.1
hsa-miR-34c-5p	0.1	0.1	0.1	0.1
hsa-miR-3529-3p	0.1	0.1	0.1	0.1
hsa-miR-3529-5p	0.1	0.1	0.1	0.1

hsa-miR-3591-3p	0.1	0.1	0.1	0.1
hsa-miR-3591-5p	0.1	0.1	0.1	0.1
hsa-miR-3605-3p	0.1	0.1	0.1	0.1
hsa-miR-3605-5p	0.1	0.1	0.1	0.1
hsa-miR-3606-3p	0.1	0.1	0.1	0.1
hsa-miR-3606-5p	0.1	0.1	0.1	0.1
hsa-miR-3607-3p	0.1	0.1	0.1	0.1
hsa-miR-3607-5p	0.1	0.1	0.1	0.1
hsa-miR-3609	0.1	0.1	0.1	0.1
hsa-miR-3610	0.1	0.1	0.1	0.1
hsa-miR-3611	0.1	0.1	0.1	0.1
hsa-miR-3612	0.1	0.1	0.1	0.1
hsa-miR-3613-3p	0.1	0.1	0.1	0.1
hsa-miR-3613-5p	0.1	0.1	0.1	0.1
hsa-miR-361-3p	3.35126	3.14447	3.20633	3.49586
hsa-miR-3614-3p	0.1	0.1	0.1	0.1
hsa-miR-3614-5p	0.1	0.1	0.1	0.1
hsa-miR-3615	0.1	0.1	0.1	0.1
hsa-miR-361-5p	13.3819	15.8911	10.8337	19.9574
hsa-miR-3616-3p	0.1	0.1	0.1	0.1
hsa-miR-3616-5p	0.1	0.1	0.1	0.1
hsa-miR-3617-3p	0.1	0.1	0.1	0.1
hsa-miR-3617-5p	0.1	0.1	0.1	0.1
hsa-miR-3618	0.1	0.1	0.1	0.1
hsa-miR-3619-3p	0.1	0.1	0.1	0.1
hsa-miR-3619-5p	0.1	0.1	0.1	0.1
hsa-miR-3620-3p	0.1	0.1	0.1	0.1
hsa-miR-3620-5p	0.1	0.1	0.1	0.1
hsa-miR-3621	0.1	0.1	0.1	0.1
hsa-miR-3622a-3p	0.1	0.1	0.1	0.1
hsa-miR-3622a-5p	0.1	0.1	0.1	0.1
hsa-miR-3622b-3p	0.1	0.1	0.1	0.1
hsa-miR-3622b-5p	0.1	0.1	0.1	0.1
hsa-miR-362-3p	2.05352	2.03239	0.1	2.32749
hsa-miR-362-5p	0.1	0.1	0.1	0.1
hsa-miR-363-3p	0.1	0.1	0.1	0.1
hsa-miR-363-5p	0.1	0.1	0.1	0.1
hsa-miR-3646	0.1	0.1	0.1	0.1
hsa-miR-3648	0.1	0.1	0.1	0.1
hsa-miR-3649	0.1	0.1	0.1	0.1
hsa-miR-3650	0.1	0.1	0.1	0.1
hsa-miR-3651	70.0739	57.5453	64.5748	43.0988
hsa-miR-3652	0.1	0.1	0.1	0.1
hsa-miR-3653-3p	2.19451	2.01258	2.95611	2.21713
hsa-miR-3653-5p	0.1	0.1	0.1	0.1
hsa-miR-3654	0.1	0.1	0.1	0.1
hsa-miR-3655	0.1	0.1	0.1	0.1
hsa-miR-3656	17.902	14.5406	27.2417	10.2191
hsa-miR-3657	0.1	0.1	0.1	0.1
hsa-miR-3658	0.1	0.1	0.1	0.1
hsa-miR-3659	0.1	0.1	0.1	5.64393
hsa-miR-365a-3p	227.258	406.225	208.617	418.263
hsa-miR-365a-5p	0.1	0.1	0.1	0.1

hsa-miR-365b-5p	0.1	0.1	0.1	0.1
hsa-miR-3660	0.1	0.1	0.1	0.1
hsa-miR-3661	0.1	0.1	0.1	0.1
hsa-miR-3662	0.1	0.1	0.1	0.1
hsa-miR-3663-3p	0.1	2.12658	0.1	0.1
hsa-miR-3663-5p	0.1	0.1	0.1	0.1
hsa-miR-3664-3p	0.1	0.1	0.1	0.1
hsa-miR-3664-5p	0.1	0.1	0.1	0.1
hsa-miR-3665	38.6992	42.3608	41.8024	36.9247
hsa-miR-3666	0.1	0.1	0.1	0.1
hsa-miR-3667-3p	0.1	0.1	0.1	0.1
hsa-miR-3667-5p	0.1	0.1	0.1	0.1
hsa-miR-3668	0.1	0.1	0.1	0.1
hsa-miR-3670	0.1	0.1	0.1	0.1
hsa-miR-3671	0.1	0.1	0.1	0.1
hsa-miR-3672	0.1	0.1	0.1	0.1
hsa-miR-367-3p	0.1	0.1	0.1	0.1
hsa-miR-3674	0.1	0.1	0.1	0.1
hsa-miR-3675-3p	0.1	0.1	0.1	0.1
hsa-miR-3675-5p	0.1	0.1	0.1	0.1
hsa-miR-367-5p	0.1	0.1	0.1	0.1
hsa-miR-3677-3p	0.1	0.1	0.1	0.1
hsa-miR-3677-5p	0.1	0.1	0.1	0.1
hsa-miR-3678-3p	0.1	0.1	0.1	0.1
hsa-miR-3678-5p	0.1	0.1	0.1	0.1
hsa-miR-3679-3p	0.1	0.1	0.1	0.1
hsa-miR-3679-5p	12.6905	15.5699	17.7593	11.1812
hsa-miR-3680-3p	0.1	0.1	0.1	0.1
hsa-miR-3680-5p	0.1	0.1	0.1	0.1
hsa-miR-3681-3p	0.1	0.1	0.1	0.1
hsa-miR-3681-5p	0.1	0.1	0.1	0.1
hsa-miR-3682-3p	0.1	0.1	0.1	0.1
hsa-miR-3682-5p	0.1	0.1	0.1	0.1
hsa-miR-3683	0.1	0.1	0.1	0.1
hsa-miR-3684	0.1	0.1	0.1	0.1
hsa-miR-3685	0.1	0.1	0.1	0.1
hsa-miR-3686	0.1	0.1	0.1	0.1
hsa-miR-3687	0.1	0.1	0.1	0.1
hsa-miR-3688-3p	0.1	0.1	0.1	0.1
hsa-miR-3688-5p	0.1	0.1	0.1	0.1
hsa-miR-3689a-3p	0.1	0.1	0.1	0.1
hsa-miR-3689a-5p	0.1	0.1	0.1	0.1
hsa-miR-3689b-3p	0.1	0.1	0.1	0.1
hsa-miR-3689d	0.1	0.1	0.1	0.1
hsa-miR-3689f	0.1	0.1	0.1	0.1
hsa-miR-3690	0.1	0.1	0.1	0.1
hsa-miR-3691-3p	0.1	0.1	0.1	0.1
hsa-miR-3691-5p	0.1	0.1	0.1	0.1
hsa-miR-3692-3p	0.1	0.1	0.1	0.1
hsa-miR-3692-5p	0.1	0.1	0.1	0.1
hsa-miR-369-3p	0.1	0.1	0.1	0.1
hsa-miR-369-5p	0.1	0.1	0.1	0.1
hsa-miR-370-3p	0.1	0.1	0.1	0.1

hsa-miR-370-5p	0.1	0.1	0.1	0.1
hsa-miR-3713	0.1	0.1	0.1	0.1
hsa-miR-3714	0.1	0.1	0.1	0.1
hsa-miR-371a-3p	0.1	0.1	0.1	0.1
hsa-miR-371a-5p	0.1	0.1	1.64944	0.1
hsa-miR-371b-3p	0.1	0.1	0.1	0.1
hsa-miR-371b-5p	8.98138	9.43944	11.3711	6.71807
hsa-miR-372-3p	0.1	0.1	0.1	0.1
hsa-miR-372-5p	0.1	0.1	0.1	0.1
hsa-miR-373-3p	0.1	0.1	0.1	0.1
hsa-miR-373-5p	0.1	0.1	0.1	0.1
hsa-miR-374a-3p	0.1	0.1	0.1	0.1
hsa-miR-374a-5p	61.6494	51.9437	26.2731	64.0069
hsa-miR-374b-3p	0.1	0.1	0.1	0.1
hsa-miR-374b-5p	38.4599	29.1973	20.0881	33.1315
hsa-miR-374c-3p	0.1	0.1	0.1	0.1
hsa-miR-374c-5p	2.35816	0.1	0.1	1.92013
hsa-miR-375	0.1	0.1	0.1	0.1
hsa-miR-376a-2-5p	0.1	0.1	0.1	0.1
hsa-miR-376a-3p	0.1	6.10518	0.1	6.14501
hsa-miR-376a-5p	0.1	0.1	0.1	0.1
hsa-miR-376b-3p	0.1	0.1	0.1	0.1
hsa-miR-376b-5p	0.1	0.1	0.1	0.1
hsa-miR-376c-3p	0.1	10.5898	0.1	10.0266
hsa-miR-376c-5p	0.1	0.1	0.1	0.1
hsa-miR-377-3p	0.1	2.19544	0.1	2.19456
hsa-miR-377-5p	0.1	0.1	0.1	0.1
hsa-miR-378a-3p	13.0882	9.32206	8.33059	9.40629
hsa-miR-378a-5p	0.1	0.1	0.1	0.1
hsa-miR-378b	0.1	0.1	0.1	0.1
hsa-miR-378c	0.1	0.1	0.1	0.1
hsa-miR-378d	0.1	0.1	0.1	0.1
hsa-miR-378e	0.1	0.1	0.1	0.1
hsa-miR-378f	0.1	0.1	0.1	0.1
hsa-miR-378g	0.1	0.1	0.1	0.1
hsa-miR-378h	0.1	0.1	0.1	0.1
hsa-miR-378i	15.098	10.7167	8.83609	11.9064
hsa-miR-378j	0.1	0.1	0.1	0.1
hsa-miR-379-3p	0.1	0.1	0.1	0.1
hsa-miR-379-5p	0.1	0.1	0.1	0.1
hsa-miR-380-3p	0.1	0.1	0.1	0.1
hsa-miR-380-5p	0.1	0.1	0.1	0.1
hsa-miR-381-3p	0.1	2.31903	0.1	0.1
hsa-miR-381-5p	0.1	0.1	0.1	0.1
hsa-miR-382-3p	0.1	0.1	0.1	0.1
hsa-miR-382-5p	0.1	0.1	0.1	0.1
hsa-miR-383-3p	0.1	0.1	0.1	0.1
hsa-miR-383-5p	0.1	0.1	0.1	0.1
hsa-miR-384	0.1	0.1	0.1	0.1
hsa-miR-3907	0.1	0.1	0.1	0.1
hsa-miR-3908	0.1	0.1	0.1	0.1
hsa-miR-3909	0.1	0.1	0.1	0.1
hsa-miR-3910	0.1	0.1	0.1	0.1

hsa-miR-3911	0.1	0.1	0.1	0.1
hsa-miR-3912-3p	0.1	0.1	0.1	0.1
hsa-miR-3912-5p	0.1	0.1	0.1	0.1
hsa-miR-3913-3p	0.1	0.1	0.1	0.1
hsa-miR-3913-5p	0.1	0.1	0.1	0.1
hsa-miR-3914	0.1	0.1	0.1	0.1
hsa-miR-3915	0.1	0.1	0.1	0.1
hsa-miR-3916	0.1	0.1	0.1	0.1
hsa-miR-3917	0.1	0.1	0.1	0.1
hsa-miR-3918	0.1	0.1	0.1	0.1
hsa-miR-3919	0.1	0.1	0.1	0.1
hsa-miR-3920	0.1	0.1	0.1	0.1
hsa-miR-3921	0.1	0.1	0.1	0.1
hsa-miR-3922-3p	0.1	0.1	0.1	0.1
hsa-miR-3922-5p	0.1	0.1	0.1	0.1
hsa-miR-3923	0.1	0.1	0.1	0.1
hsa-miR-3924	0.1	0.1	0.1	0.1
hsa-miR-3925-3p	0.1	0.1	0.1	0.1
hsa-miR-3925-5p	0.1	0.1	0.1	0.1
hsa-miR-3926	0.1	0.1	0.1	0.1
hsa-miR-3927-3p	0.1	0.1	0.1	0.1
hsa-miR-3927-5p	0.1	0.1	0.1	0.1
hsa-miR-3928-3p	0.1	0.1	0.1	0.1
hsa-miR-3928-5p	0.1	0.1	0.1	0.1
hsa-miR-3929	0.1	0.1	0.1	0.1
hsa-miR-3934-3p	0.1	0.1	0.1	0.1
hsa-miR-3934-5p	18.4179	32.7369	122.327	24.3436
hsa-miR-3935	0.1	0.1	0.1	0.1
hsa-miR-3936	0.1	0.1	0.1	0.1
hsa-miR-3937	0.1	0.1	0.1	0.1
hsa-miR-3938	0.1	0.1	0.1	0.1
hsa-miR-3939	0.1	0.1	0.1	0.1
hsa-miR-3940-3p	0.1	0.1	0.1	0.1
hsa-miR-3940-5p	4.15948	4.39773	5.26132	2.75453
hsa-miR-3941	0.1	0.1	0.1	0.1
hsa-miR-3942-3p	0.1	0.1	0.1	0.1
hsa-miR-3942-5p	0.1	0.1	0.1	0.1
hsa-miR-3943	0.1	0.1	0.1	0.1
hsa-miR-3944-3p	0.1	0.1	0.1	0.1
hsa-miR-3944-5p	0.1	0.1	0.1	0.1
hsa-miR-3945	0.1	0.1	0.1	0.1
hsa-miR-3960	75.7907	74.3314	89.5175	48.7852
hsa-miR-3972	0.1	0.1	0.1	0.1
hsa-miR-3973	0.1	0.1	0.1	0.1
hsa-miR-3974	0.1	0.1	0.1	0.1
hsa-miR-3975	0.1	0.1	0.1	0.1
hsa-miR-3976	0.1	0.1	0.1	0.1
hsa-miR-3977	0.1	0.1	0.1	0.1
hsa-miR-3978	0.1	0.1	0.1	0.1
hsa-miR-409-3p	0.1	0.1	0.1	0.1
hsa-miR-409-5p	0.1	0.1	0.1	0.1
hsa-miR-410-3p	0.1	0.1	0.1	0.1
hsa-miR-410-5p	0.1	0.1	0.1	0.1

hsa-miR-411-3p	0.1	0.1	0.1	0.1
hsa-miR-411-5p	0.1	0.1	0.1	0.1
hsa-miR-412-3p	0.1	0.1	0.1	0.1
hsa-miR-412-5p	0.1	0.1	0.1	0.1
hsa-miR-421	1.55814	0.1	0.1	0.1
hsa-miR-422a	0.1	0.1	0.1	0.1
hsa-miR-423-3p	0.1	0.1	0.1	0.1
hsa-miR-423-5p	11.4304	9.74081	9.83569	10.3305
hsa-miR-424-3p	0.1	1.81764	0.1	0.1
hsa-miR-424-5p	119.569	380.303	79.8575	386.722
hsa-miR-4251	0.1	0.1	0.1	0.1
hsa-miR-4252	0.1	0.1	0.1	0.1
hsa-miR-4253	0.1	0.1	0.1	0.1
hsa-miR-425-3p	0.1	0.1	0.1	0.1
hsa-miR-4254	0.1	0.1	0.1	0.1
hsa-miR-4255	0.1	0.1	0.1	0.1
hsa-miR-425-5p	18.3279	14.1208	17.215	13.3263
hsa-miR-4256	0.1	0.1	0.1	0.1
hsa-miR-4257	2.28618	3.0121	6.09394	1.89408
hsa-miR-4258	0.1	0.1	0.1	0.1
hsa-miR-4259	0.1	0.1	0.1	0.1
hsa-miR-4260	0.1	0.1	0.1	0.1
hsa-miR-4261	0.1	0.1	0.1	0.1
hsa-miR-4262	0.1	0.1	0.1	0.1
hsa-miR-4263	0.1	0.1	0.1	0.1
hsa-miR-4264	0.1	0.1	0.1	0.1
hsa-miR-4265	0.1	0.1	0.1	0.1
hsa-miR-4266	0.1	0.1	0.1	0.1
hsa-miR-4267	0.1	0.1	0.1	0.1
hsa-miR-4268	0.1	0.1	0.1	0.1
hsa-miR-4269	0.1	0.1	0.1	0.1
hsa-miR-4270	0.1	0.1	1.92804	0.1
hsa-miR-4271	1.49888	0.1	4.718	0.1
hsa-miR-4272	0.1	0.1	0.1	0.1
hsa-miR-4273	0.1	0.1	0.1	0.1
hsa-miR-4274	0.1	0.1	0.1	0.1
hsa-miR-4275	0.1	0.1	0.1	0.1
hsa-miR-4276	0.1	0.1	0.1	0.1
hsa-miR-4277	0.1	0.1	0.1	0.1
hsa-miR-4278	0.1	0.1	0.1	0.1
hsa-miR-4279	0.1	0.1	0.1	0.1
hsa-miR-4280	0.1	0.1	0.1	0.1
hsa-miR-4281	28.0075	30.086	31.1567	22.4809
hsa-miR-4282	0.1	0.1	0.1	0.1
hsa-miR-4283	0.1	0.1	0.1	0.1
hsa-miR-4284	1391.55	1054.89	980.697	1423.15
hsa-miR-4285	0.1	0.1	0.1	0.1
hsa-miR-4286	41.4902	124.566	40.3273	174.868
hsa-miR-4287	0.1	0.1	0.1	0.1
hsa-miR-4288	0.1	0.1	0.1	0.1
hsa-miR-4289	0.1	0.1	0.1	0.1
hsa-miR-429	0.1	0.1	0.1	0.1
hsa-miR-4290	0.1	0.1	0.1	0.1

hsa-miR-4291	12.124	18.0644	10.5848	22.5631
hsa-miR-4292	0.1	0.1	0.1	0.1
hsa-miR-4293	0.1	0.1	0.1	0.1
hsa-miR-4294	0.1	0.1	0.1	0.1
hsa-miR-4295	0.1	0.1	0.1	0.1
hsa-miR-4296	0.1	0.1	0.1	0.1
hsa-miR-4297	0.1	0.1	0.1	0.1
hsa-miR-4298	1.69193	1.89246	9.34329	0.1
hsa-miR-4299	72.912	82.2657	85.9139	70.5878
hsa-miR-4300	0.1	0.1	0.1	0.1
hsa-miR-4301	0.1	0.1	0.1	0.1
hsa-miR-4302	0.1	0.1	0.1	0.1
hsa-miR-4303	0.1	0.1	0.1	0.1
hsa-miR-4304	0.1	0.1	0.1	0.1
hsa-miR-4305	0.1	0.1	0.1	0.1
hsa-miR-4306	11.9103	12.4708	11.8134	17.3069
hsa-miR-4307	0.1	0.1	0.1	0.1
hsa-miR-4308	0.1	0.1	0.1	0.1
hsa-miR-4309	0.1	0.1	0.1	0.1
hsa-miR-4310	0.1	0.1	0.1	0.1
hsa-miR-4311	0.1	0.1	0.1	0.1
hsa-miR-4312	0.1	0.1	0.1	0.1
hsa-miR-4313	0.1	0.1	0.1	0.1
hsa-miR-431-3p	0.1	0.1	0.1	0.1
hsa-miR-4314	0.1	0.1	0.1	0.1
hsa-miR-4315	0.1	0.1	0.1	0.1
hsa-miR-431-5p	0.1	0.1	0.1	0.1
hsa-miR-4316	0.1	0.1	0.1	0.1
hsa-miR-4317	0.1	2.74384	0.1	3.17479
hsa-miR-4318	0.1	0.1	0.1	0.1
hsa-miR-4319	0.1	0.1	0.1	0.1
hsa-miR-4320	0.1	0.1	0.1	0.1
hsa-miR-4321	0.1	0.1	0.1	0.1
hsa-miR-4322	0.1	0.1	0.1	0.1
hsa-miR-4323	0.1	0.1	0.1	0.1
hsa-miR-432-3p	0.1	0.1	0.1	0.1
hsa-miR-4324	1.19649	1.96567	1.3843	2.73288
hsa-miR-4325	0.1	0.1	0.1	0.1
hsa-miR-432-5p	0.1	0.1	0.1	0.1
hsa-miR-4326	0.1	0.1	0.1	0.1
hsa-miR-4327	0.1	0.1	0.1	0.1
hsa-miR-4328	0.1	0.1	0.1	0.1
hsa-miR-4329	0.1	0.1	0.1	0.1
hsa-miR-4330	0.1	0.1	0.1	0.1
hsa-miR-433-3p	0.1	0.1	0.1	0.1
hsa-miR-433-5p	0.1	0.1	0.1	0.1
hsa-miR-4417	0.1	0.1	0.1	0.1
hsa-miR-4418	0.1	0.1	0.1	0.1
hsa-miR-4419a	0.1	0.1	0.1	0.1
hsa-miR-4419b	0.1	0.1	0.1	0.1
hsa-miR-4420	0.1	0.1	0.1	0.1
hsa-miR-4421	0.1	0.1	0.1	0.1
hsa-miR-4422	0.1	0.1	0.1	0.1

hsa-miR-4423-3p	0.1	0.1	0.1	0.1
hsa-miR-4423-5p	0.1	0.1	0.1	0.1
hsa-miR-4424	0.1	0.1	0.1	0.1
hsa-miR-4425	0.1	0.1	0.1	0.1
hsa-miR-4426	0.1	0.1	0.1	0.1
hsa-miR-4427	0.1	0.1	0.1	0.1
hsa-miR-4428	16.9306	18.717	12.4728	15.3476
hsa-miR-4429	0.1	0.1	0.1	0.1
hsa-miR-4430	43.9213	43.7606	98.0802	31.4364
hsa-miR-4431	0.1	0.1	0.1	0.1
hsa-miR-4432	0.1	0.1	0.1	0.1
hsa-miR-4433a-3p	0.1	0.1	0.1	0.1
hsa-miR-4433a-5p	0.1	0.1	0.1	0.1
hsa-miR-4433b-3p	0.1	0.1	0.1	0.1
hsa-miR-4434	0.1	0.1	0.1	0.1
hsa-miR-4435	0.1	0.1	0.1	0.1
hsa-miR-4436a	0.1	0.1	0.1	0.1
hsa-miR-4436b-3p	0.1	0.1	0.1	0.1
hsa-miR-4436b-5p	0.1	0.1	0.1	0.1
hsa-miR-4437	0.1	0.1	0.1	0.1
hsa-miR-4438	0.1	0.1	0.1	0.1
hsa-miR-4439	0.1	0.1	0.1	0.1
hsa-miR-4440	0.1	0.1	0.1	0.1
hsa-miR-4441	0.1	0.1	0.1	0.1
hsa-miR-4442	7.11919	7.13877	9.97065	6.41275
hsa-miR-4443	15.0602	18.4468	21.1287	16.3111
hsa-miR-4444	0.1	0.1	0.1	0.1
hsa-miR-4445-3p	0.1	0.1	0.1	0.1
hsa-miR-4445-5p	0.1	0.1	0.1	0.1
hsa-miR-4446-3p	0.1	0.1	0.1	0.1
hsa-miR-4446-5p	0.1	0.1	0.1	0.1
hsa-miR-4447	0.1	0.1	0.1	0.1
hsa-miR-4448	0.1	0.1	0.1	0.1
hsa-miR-4449	0.1	0.1	0.1	0.1
hsa-miR-4450	0.1	0.1	0.1	0.1
hsa-miR-4451	0.1	0.1	0.1	0.1
hsa-miR-4452	0.1	0.1	0.1	0.1
hsa-miR-4453	0.1	0.1	0.1	0.1
hsa-miR-4455	0.1	0.1	0.1	0.1
hsa-miR-4456	0.1	0.1	0.1	0.1
hsa-miR-4457	0.1	0.1	0.1	0.1
hsa-miR-4458	0.1	0.1	0.1	0.1
hsa-miR-4459	150.195	172.802	177.343	148.599
hsa-miR-4460	0.1	0.1	0.1	0.1
hsa-miR-4461	0.1	0.1	0.1	0.1
hsa-miR-4462	0.1	0.1	0.1	0.1
hsa-miR-4463	1.66454	0.1	3.90919	0.1
hsa-miR-4464	0.1	0.1	0.1	0.1
hsa-miR-4465	26.7508	19.2959	20.0145	14.3408
hsa-miR-4466	22.2948	20.8094	25.3445	14.1869
hsa-miR-4467	0.1	0.1	0.1	0.1
hsa-miR-4468	0.1	0.1	0.1	0.1
hsa-miR-4469	0.1	0.1	0.1	0.1

hsa-miR-4470	0.1	0.1	0.1	0.1
hsa-miR-4471	0.1	0.1	0.1	0.1
hsa-miR-4472	0.1	0.1	0.1	0.1
hsa-miR-4473	0.1	0.1	0.1	0.1
hsa-miR-4474-3p	0.1	0.1	0.1	0.1
hsa-miR-4474-5p	0.1	0.1	0.1	0.1
hsa-miR-4475	0.1	0.1	0.1	0.1
hsa-miR-4476	0.1	0.1	0.1	0.1
hsa-miR-4477a	0.1	0.1	0.1	0.1
hsa-miR-4477b	0.1	0.1	0.1	0.1
hsa-miR-4478	8.99148	8.22828	10.2622	7.07031
hsa-miR-4479	0.1	0.1	0.1	0.1
hsa-miR-448	0.1	0.1	0.1	0.1
hsa-miR-4480	0.1	0.1	0.1	0.1
hsa-miR-4481	0.1	0.1	0.1	0.1
hsa-miR-4482-3p	0.1	0.1	0.1	0.1
hsa-miR-4482-5p	0.1	0.1	0.1	0.1
hsa-miR-4483	0.1	0.1	0.1	0.1
hsa-miR-4484	0.1	0.1	0.1	0.1
hsa-miR-4485-3p	15.4342	12.1622	19.6751	11.925
hsa-miR-4485-5p	120.323	98.9308	204.732	109.524
hsa-miR-4486	0.1	0.1	0.1	0.1
hsa-miR-4487	0.1	0.1	2.50005	0.1
hsa-miR-4488	0.1	0.1	2.43947	0.1
hsa-miR-4489	0.1	0.1	0.1	0.1
hsa-miR-4490	0.1	0.1	0.1	0.1
hsa-miR-4491	0.1	0.1	0.1	0.1
hsa-miR-4492	0.1	0.1	0.1	0.1
hsa-miR-4493	0.1	0.1	0.1	0.1
hsa-miR-4494	0.1	0.1	0.1	0.1
hsa-miR-4495	0.1	0.1	0.1	0.1
hsa-miR-4496	0.1	0.1	0.1	0.1
hsa-miR-4497	11.1596	11.6245	10.408	9.8214
hsa-miR-4498	0.1	0.1	0.1	0.1
hsa-miR-4499	7.71996	5.68889	6.15204	3.90669
hsa-miR-449a	0.1	0.1	0.1	0.1
hsa-miR-449b-3p	0.1	0.1	0.1	0.1
hsa-miR-449b-5p	0.1	0.1	0.1	0.1
hsa-miR-449c-3p	0.1	0.1	0.1	0.1
hsa-miR-449c-5p	0.1	0.1	0.1	0.1
hsa-miR-4500	0.1	0.1	0.1	0.1
hsa-miR-4501	0.1	0.1	0.1	0.1
hsa-miR-4502	0.1	0.1	0.1	0.1
hsa-miR-4503	0.1	0.1	0.1	0.1
hsa-miR-4504	0.1	0.1	0.1	0.1
hsa-miR-4505	45.2324	48.0455	51.56	40.2529
hsa-miR-4506	0.1	0.1	0.1	0.1
hsa-miR-4507	39.8543	40.2202	45.932	35.8981
hsa-miR-4508	0.1	0.1	0.1	0.1
hsa-miR-4509	0.1	0.1	0.1	0.1
hsa-miR-450a-1-3p	0.1	0.1	0.1	0.1
hsa-miR-450a-2-3p	0.1	0.1	0.1	0.1
hsa-miR-450a-5p	6.98493	11.19	5.12409	17.4655

hsa-miR-450b-3p	0.1	0.1	0.1	0.1
hsa-miR-450b-5p	0.1	0.1	0.1	0.1
hsa-miR-4510	0.1	0.1	0.1	0.1
hsa-miR-4511	0.1	0.1	0.1	0.1
hsa-miR-4512	0.1	0.1	0.1	0.1
hsa-miR-4513	0.1	0.1	0.1	0.1
hsa-miR-4514	0.1	0.1	0.1	0.1
hsa-miR-4515	0.1	0.1	0.1	0.1
hsa-miR-4516	130.859	119.303	141.909	83.6025
hsa-miR-4517	0.1	0.1	0.1	0.1
hsa-miR-4518	0.1	0.1	0.1	0.1
hsa-miR-4519	0.1	0.1	0.1	0.1
hsa-miR-451a	0.1	0.1	0.1	0.1
hsa-miR-451b	0.1	0.1	0.1	0.1
hsa-miR-4520-2-3p	0.1	0.1	0.1	0.1
hsa-miR-4520-3p	0.1	0.1	0.1	0.1
hsa-miR-4520-5p	0.1	0.1	0.1	0.1
hsa-miR-4521	1.79479	0.1	4.91098	0.1
hsa-miR-4522	0.1	0.1	0.1	0.1
hsa-miR-4523	0.1	0.1	0.1	0.1
hsa-miR-452-3p	0.1	0.1	0.1	0.1
hsa-miR-4524a-3p	0.1	0.1	0.1	0.1
hsa-miR-4524a-5p	0.1	0.1	0.1	0.1
hsa-miR-4524b-3p	0.1	0.1	0.1	0.1
hsa-miR-4524b-5p	0.1	0.1	0.1	0.1
hsa-miR-4525	0.1	0.1	0.1	0.1
hsa-miR-452-5p	9.20155	5.12187	9.29683	7.5908
hsa-miR-4526	0.1	0.1	0.1	0.1
hsa-miR-4527	0.1	0.1	0.1	0.1
hsa-miR-4528	0.1	0.1	0.1	0.1
hsa-miR-4529-3p	0.1	0.1	0.1	0.1
hsa-miR-4529-5p	0.1	0.1	0.1	0.1
hsa-miR-4530	73.8861	69.4312	71.6951	49.9743
hsa-miR-4531	0.1	0.1	0.1	0.1
hsa-miR-4532	10.8484	7.33903	16.935	5.3998
hsa-miR-4533	0.1	0.1	0.1	0.1
hsa-miR-4534	0.1	0.1	0.1	0.1
hsa-miR-4535	0.1	0.1	0.1	0.1
hsa-miR-4536-3p	0.1	0.1	0.1	0.1
hsa-miR-4536-5p	0.1	0.1	0.1	0.1
hsa-miR-4537	0.1	0.1	0.1	0.1
hsa-miR-4538	0.1	0.1	0.1	0.1
hsa-miR-4539	0.1	0.1	0.1	0.1
hsa-miR-4540	0.1	0.1	0.1	0.1
hsa-miR-454-3p	3.80208	3.80192	4.63588	4.03433
hsa-miR-454-5p	0.1	0.1	1.03362	0.1
hsa-miR-455-3p	3.44541	23.7368	2.8511	25.9739
hsa-miR-455-5p	0.1	2.43483	0.1	2.81788
hsa-miR-4632-3p	0.1	0.1	0.1	0.1
hsa-miR-4632-5p	0.1	0.1	0.1	0.1
hsa-miR-4633-3p	0.1	0.1	0.1	0.1
hsa-miR-4633-5p	0.1	0.1	0.1	0.1
hsa-miR-4634	0.1	0.1	0.1	0.1

hsa-miR-4635	0.1	0.1	0.1	0.1
hsa-miR-4636	0.1	0.1	0.1	0.1
hsa-miR-4637	0.1	0.1	0.1	0.1
hsa-miR-4638-3p	0.1	0.1	0.1	0.1
hsa-miR-4638-5p	0.1	0.1	0.1	0.1
hsa-miR-4639-3p	0.1	0.1	0.1	0.1
hsa-miR-4639-5p	0.1	0.1	0.1	0.1
hsa-miR-4640-3p	0.1	0.1	0.1	0.1
hsa-miR-4640-5p	0.1	0.1	0.1	0.1
hsa-miR-4641	0.1	0.1	0.1	0.1
hsa-miR-4642	0.1	0.1	0.1	0.1
hsa-miR-4643	0.1	0.1	0.1	0.1
hsa-miR-4644	0.1	0.1	0.1	0.1
hsa-miR-4645-3p	0.1	0.1	0.1	0.1
hsa-miR-4645-5p	0.1	0.1	0.1	0.1
hsa-miR-4646-3p	0.1	0.1	0.1	0.1
hsa-miR-4646-5p	0.1	0.1	0.1	0.1
hsa-miR-4647	0.1	0.1	0.1	0.1
hsa-miR-4648	0.1	0.1	0.1	0.1
hsa-miR-4649-3p	1.56848	0.1	0.1	0.1
hsa-miR-4649-5p	0.1	0.1	0.1	0.1
hsa-miR-4650-3p	0.1	0.1	0.1	0.1
hsa-miR-4650-5p	0.1	0.1	0.1	0.1
hsa-miR-4651	0.1	0.1	0.1	0.1
hsa-miR-4652-3p	0.1	0.1	0.1	0.1
hsa-miR-4652-5p	0.1	0.1	0.1	0.1
hsa-miR-4653-3p	7.55247	7.03082	6.78211	7.52295
hsa-miR-4653-5p	0.1	0.1	0.1	0.1
hsa-miR-4654	0.1	0.1	0.1	0.1
hsa-miR-4655-3p	0.1	0.1	0.1	0.1
hsa-miR-4655-5p	0.1	0.1	0.1	0.1
hsa-miR-4656	3.79456	3.65956	3.07688	2.92432
hsa-miR-4657	0.1	0.1	0.1	0.1
hsa-miR-4658	0.1	0.1	0.1	0.1
hsa-miR-4659a-3p	0.1	0.1	0.1	0.1
hsa-miR-4659a-5p	0.1	0.1	0.1	0.1
hsa-miR-4659b-3p	0.1	0.1	0.1	0.1
hsa-miR-4659b-5p	0.1	0.1	0.1	0.1
hsa-miR-466	0.1	0.1	0.1	0.1
hsa-miR-4660	0.1	0.1	0.1	0.1
hsa-miR-4661-3p	0.1	0.1	0.1	0.1
hsa-miR-4661-5p	0.1	0.1	0.1	0.1
hsa-miR-4662a-3p	0.1	0.1	0.1	0.1
hsa-miR-4662a-5p	0.1	0.1	0.1	0.1
hsa-miR-4662b	0.1	0.1	0.1	0.1
hsa-miR-4663	0.1	0.1	0.1	0.1
hsa-miR-4664-3p	0.1	0.1	0.1	0.1
hsa-miR-4664-5p	0.1	0.1	0.1	0.1
hsa-miR-4665-3p	0.1	0.1	0.1	0.1
hsa-miR-4665-5p	0.1	0.1	0.1	0.1
hsa-miR-4666a-3p	0.1	0.1	0.1	0.1
hsa-miR-4666a-5p	0.1	0.1	0.1	0.1
hsa-miR-4666b	0.1	0.1	0.1	0.1

hsa-miR-4667-3p	0.1	0.1	0.1	0.1
hsa-miR-4667-5p	0.1	0.1	0.1	0.1
hsa-miR-4668-3p	0.1	0.1	0.1	0.1
hsa-miR-4668-5p	0.1	0.1	0.1	0.1
hsa-miR-4669	6.10553	6.14013	6.02786	4.09671
hsa-miR-4670-3p	0.1	0.1	0.1	0.1
hsa-miR-4670-5p	0.1	0.1	0.1	0.1
hsa-miR-4671-3p	0.1	0.1	0.1	0.1
hsa-miR-4671-5p	0.1	0.1	0.1	0.1
hsa-miR-4672	89.5601	59.7859	104.578	52.2676
hsa-miR-4673	0.1	0.1	0.1	0.1
hsa-miR-4674	0.1	0.1	0.1	0.1
hsa-miR-4675	0.1	0.1	0.1	0.1
hsa-miR-4676-3p	0.1	0.1	0.1	0.1
hsa-miR-4676-5p	0.1	0.1	0.1	0.1
hsa-miR-4677-3p	0.1	0.1	0.1	0.1
hsa-miR-4677-5p	0.1	0.1	0.1	0.1
hsa-miR-4678	0.1	0.1	0.1	0.1
hsa-miR-4679	0.1	0.1	0.1	0.1
hsa-miR-4680-3p	0.1	0.1	0.1	0.1
hsa-miR-4680-5p	0.1	0.1	0.1	0.1
hsa-miR-4681	0.1	0.1	0.1	0.1
hsa-miR-4682	0.1	0.1	0.1	0.1
hsa-miR-4683	0.1	0.1	0.1	0.1
hsa-miR-4684-3p	0.1	0.1	0.1	0.1
hsa-miR-4684-5p	0.1	0.1	0.1	0.1
hsa-miR-4685-3p	0.1	0.1	0.1	0.1
hsa-miR-4685-5p	0.1	0.1	0.1	0.1
hsa-miR-4686	0.1	0.1	0.1	0.1
hsa-miR-4687-3p	15.2082	17.3587	19.2429	12.0618
hsa-miR-4687-5p	0.1	0.1	0.1	0.1
hsa-miR-4688	0.1	0.1	0.1	0.1
hsa-miR-4689	0.1	0.1	0.1	0.1
hsa-miR-4690-3p	0.1	0.1	0.1	0.1
hsa-miR-4690-5p	0.1	0.1	0.1	0.1
hsa-miR-4691-3p	0.1	0.1	0.1	0.1
hsa-miR-4691-5p	0.1	0.1	0.1	0.1
hsa-miR-4692	0.1	0.1	0.1	0.1
hsa-miR-4693-3p	0.1	0.1	0.1	0.1
hsa-miR-4693-5p	0.1	0.1	0.1	0.1
hsa-miR-4694-3p	0.1	0.1	0.1	0.1
hsa-miR-4694-5p	0.1	0.1	0.1	0.1
hsa-miR-4695-3p	0.1	0.1	0.1	0.1
hsa-miR-4695-5p	0.1	0.1	0.1	0.1
hsa-miR-4696	0.1	0.1	0.1	0.1
hsa-miR-4697-3p	0.1	0.1	0.1	0.1
hsa-miR-4697-5p	0.1	0.1	0.1	0.1
hsa-miR-4698	0.1	0.1	0.1	0.1
hsa-miR-4699-3p	0.1	0.1	0.1	0.1
hsa-miR-4699-5p	0.1	0.1	0.1	0.1
hsa-miR-4700-3p	0.1	0.1	0.1	0.1
hsa-miR-4700-5p	0.1	0.1	0.1	0.1
hsa-miR-4701-3p	0.1	0.1	0.1	0.1

hsa-miR-4701-5p	0.1	0.1	0.1	0.1
hsa-miR-4703-3p	0.1	0.1	0.1	0.1
hsa-miR-4703-5p	0.1	0.1	0.1	0.1
hsa-miR-4704-3p	0.1	0.1	0.1	0.1
hsa-miR-4704-5p	0.1	0.1	0.1	0.1
hsa-miR-4705	0.1	0.1	0.1	0.1
hsa-miR-4706	0.1	0.1	0.1	0.1
hsa-miR-4707-3p	0.1	0.1	0.1	0.1
hsa-miR-4707-5p	0.1	0.1	0.1	0.1
hsa-miR-4708-3p	0.1	0.1	0.1	0.1
hsa-miR-4708-5p	0.1	0.1	0.1	0.1
hsa-miR-4709-3p	0.1	0.1	0.1	0.1
hsa-miR-4709-5p	0.1	0.1	0.1	0.1
hsa-miR-4710	0.1	0.1	0.1	0.1
hsa-miR-4711-3p	0.1	0.1	0.1	0.1
hsa-miR-4711-5p	0.1	0.1	0.1	0.1
hsa-miR-4712-3p	0.1	0.1	0.1	0.1
hsa-miR-4712-5p	0.1	0.1	0.1	0.1
hsa-miR-4713-3p	18.3116	18.2822	16.5934	20.5511
hsa-miR-4713-5p	0.1	0.1	0.1	0.1
hsa-miR-4714-3p	0.1	0.1	0.1	0.1
hsa-miR-4714-5p	0.1	0.1	0.1	0.1
hsa-miR-4715-3p	0.1	0.1	0.1	0.1
hsa-miR-4715-5p	0.1	0.1	0.1	0.1
hsa-miR-4716-3p	4.71197	4.72807	5.41665	4.92472
hsa-miR-4716-5p	0.1	0.1	0.1	0.1
hsa-miR-4717-3p	0.1	0.1	0.1	0.1
hsa-miR-4717-5p	0.1	0.1	0.1	0.1
hsa-miR-4718	0.1	0.1	0.1	0.1
hsa-miR-4719	0.1	0.1	0.1	0.1
hsa-miR-4720-3p	0.1	0.1	0.1	0.1
hsa-miR-4720-5p	0.1	0.1	0.1	0.1
hsa-miR-4721	11.138	10.5844	13.7326	9.59905
hsa-miR-4722-3p	0.1	0.1	0.1	0.1
hsa-miR-4722-5p	0.1	0.1	0.1	0.1
hsa-miR-4723-3p	0.1	0.1	0.1	0.1
hsa-miR-4723-5p	0.1	0.1	0.1	0.1
hsa-miR-4724-3p	0.1	0.1	0.1	0.1
hsa-miR-4724-5p	0.1	0.1	0.1	0.1
hsa-miR-4725-3p	0.1	0.1	0.1	0.1
hsa-miR-4725-5p	0.1	0.1	0.1	0.1
hsa-miR-4726-3p	0.1	0.1	0.1	0.1
hsa-miR-4726-5p	0.1	0.1	0.1	0.1
hsa-miR-4727-3p	0.1	0.1	0.1	0.1
hsa-miR-4727-5p	0.1	0.1	0.1	0.1
hsa-miR-4728-3p	0.1	0.1	0.1	0.1
hsa-miR-4728-5p	21.849	21.5353	25.8723	21.9524
hsa-miR-4729	0.1	0.1	0.1	0.1
hsa-miR-4730	0.1	0.1	0.1	0.1
hsa-miR-4731-3p	0.1	0.1	0.1	0.1
hsa-miR-4731-5p	0.1	0.1	0.1	0.1
hsa-miR-4732-3p	0.1	0.1	0.1	0.1
hsa-miR-4732-5p	0.1	0.1	0.1	0.1

hsa-miR-4733-3p	0.1	0.1	0.1	0.1
hsa-miR-4733-5p	0.1	0.1	0.1	0.1
hsa-miR-4734	0.1	0.1	0.1	0.1
hsa-miR-4735-3p	0.1	0.1	0.1	0.1
hsa-miR-4735-5p	0.1	0.1	0.1	0.1
hsa-miR-4736	0.1	0.1	0.1	0.1
hsa-miR-4737	0.1	0.1	0.1	0.1
hsa-miR-4738-3p	0.1	0.1	0.1	0.1
hsa-miR-4738-5p	0.1	0.1	0.1	0.1
hsa-miR-4739	6.28922	5.93873	7.72145	4.74066
hsa-miR-4740-3p	0.1	0.1	0.1	0.1
hsa-miR-4740-5p	0.1	0.1	0.1	0.1
hsa-miR-4741	1.50626	1.96221	5.28149	0.1
hsa-miR-4742-3p	0.1	0.1	0.1	0.1
hsa-miR-4742-5p	0.1	0.1	0.1	0.1
hsa-miR-4743-3p	0.1	0.1	0.1	0.1
hsa-miR-4743-5p	0.1	0.1	0.1	0.1
hsa-miR-4744	0.1	0.1	0.1	0.1
hsa-miR-4745-3p	0.1	0.1	0.1	0.1
hsa-miR-4745-5p	0.1	0.1	0.1	0.1
hsa-miR-4746-3p	6.36476	4.76702	9.70366	3.41283
hsa-miR-4746-5p	0.1	0.1	0.1	0.1
hsa-miR-4747-3p	0.1	0.1	0.1	0.1
hsa-miR-4747-5p	0.1	0.1	0.1	0.1
hsa-miR-4748	0.1	0.1	0.1	0.1
hsa-miR-4749-3p	0.1	0.1	0.1	0.1
hsa-miR-4749-5p	0.1	0.1	0.1	0.1
hsa-miR-4750-3p	0.1	0.1	0.1	0.1
hsa-miR-4750-5p	0.1	0.1	0.1	0.1
hsa-miR-4751	0.1	0.1	0.1	0.1
hsa-miR-4752	0.1	0.1	0.1	0.1
hsa-miR-4753-3p	0.1	0.1	0.1	0.1
hsa-miR-4753-5p	0.1	0.1	0.1	0.1
hsa-miR-4754	0.1	0.1	1.98089	0.1
hsa-miR-4755-3p	0.1	0.1	0.1	0.1
hsa-miR-4755-5p	0.1	0.1	0.1	0.1
hsa-miR-4756-3p	0.1	0.1	0.1	0.1
hsa-miR-4756-5p	0.1	0.1	0.1	0.1
hsa-miR-4757-3p	0.1	0.1	0.1	0.1
hsa-miR-4757-5p	0.1	0.1	0.1	0.1
hsa-miR-4758-3p	0.1	0.1	0.1	0.1
hsa-miR-4758-5p	0.1	0.1	0.1	0.1
hsa-miR-4759	0.1	0.1	0.1	0.1
hsa-miR-4760-3p	0.1	0.1	0.1	0.1
hsa-miR-4760-5p	0.1	0.1	0.1	0.1
hsa-miR-4761-3p	0.1	0.1	0.1	0.1
hsa-miR-4761-5p	0.1	0.1	0.1	0.1
hsa-miR-4762-3p	0.1	0.1	0.1	0.1
hsa-miR-4762-5p	0.1	0.1	0.1	0.1
hsa-miR-4763-3p	7.32581	7.40442	9.34523	4.24792
hsa-miR-4763-5p	0.1	0.1	0.1	0.1
hsa-miR-4764-3p	0.1	0.1	0.1	0.1
hsa-miR-4764-5p	0.1	0.1	0.1	0.1

hsa-miR-4765	0.1	0.1	0.1	0.1
hsa-miR-4766-3p	0.1	0.1	0.1	0.1
hsa-miR-4766-5p	0.1	0.1	0.1	0.1
hsa-miR-4767	0.1	0.1	0.1	0.1
hsa-miR-4768-3p	0.1	0.1	0.1	0.1
hsa-miR-4768-5p	0.1	0.1	0.1	0.1
hsa-miR-4769-3p	0.1	0.1	0.1	0.1
hsa-miR-4769-5p	0.1	0.1	0.1	0.1
hsa-miR-4770	0.1	0.1	0.1	0.1
hsa-miR-4771	0.1	0.1	0.1	0.1
hsa-miR-4772-3p	0.1	0.1	0.1	0.1
hsa-miR-4772-5p	0.1	0.1	0.1	0.1
hsa-miR-4773	0.1	0.1	0.1	0.1
hsa-miR-4774-3p	0.1	0.1	0.1	0.1
hsa-miR-4774-5p	0.1	0.1	0.1	0.1
hsa-miR-4775	0.1	0.1	0.1	0.1
hsa-miR-4776-3p	0.1	0.1	0.1	0.1
hsa-miR-4776-5p	0.1	0.1	0.1	0.1
hsa-miR-4777-3p	0.1	0.1	0.1	0.1
hsa-miR-4777-5p	0.1	0.1	0.1	0.1
hsa-miR-4778-3p	0.1	0.1	0.1	0.1
hsa-miR-4778-5p	0.1	0.1	0.1	0.1
hsa-miR-4779	0.1	0.1	0.1	0.1
hsa-miR-4780	0.1	0.1	0.1	0.1
hsa-miR-4781-3p	0.1	0.1	0.1	0.1
hsa-miR-4781-5p	0.1	0.1	0.1	0.1
hsa-miR-4782-3p	0.1	0.1	0.1	0.1
hsa-miR-4782-5p	0.1	0.1	0.1	0.1
hsa-miR-4783-3p	0.1	0.1	0.1	0.1
hsa-miR-4783-5p	0.1	0.1	0.1	0.1
hsa-miR-4784	0.1	0.1	0.1	0.1
hsa-miR-4785	0.1	0.1	0.1	0.1
hsa-miR-4786-3p	0.1	0.1	0.1	0.1
hsa-miR-4786-5p	0.1	0.1	0.1	0.1
hsa-miR-4787-3p	0.1	0.1	0.1	0.1
hsa-miR-4787-5p	18.7862	21.3606	25.3471	18.5132
hsa-miR-4788	18.3858	13.0648	55.9701	9.48473
hsa-miR-4789-3p	0.1	0.1	0.1	0.1
hsa-miR-4789-5p	0.1	0.1	0.1	0.1
hsa-miR-4790-3p	0.1	0.1	0.1	0.1
hsa-miR-4790-5p	0.1	0.1	0.1	0.1
hsa-miR-4791	0.1	0.1	0.1	0.1
hsa-miR-4792	0.1	0.1	0.1	0.1
hsa-miR-4793-3p	0.1	0.1	0.1	0.1
hsa-miR-4793-5p	0.1	0.1	0.1	0.1
hsa-miR-4794	0.1	0.1	0.1	0.1
hsa-miR-4795-3p	0.1	0.1	0.1	0.1
hsa-miR-4795-5p	0.1	0.1	0.1	0.1
hsa-miR-4796-3p	0.1	0.1	0.1	0.1
hsa-miR-4796-5p	0.1	0.1	0.1	0.1
hsa-miR-4797-3p	0.1	0.1	0.1	0.1
hsa-miR-4797-5p	0.1	0.1	0.1	0.1
hsa-miR-4798-3p	0.1	0.1	0.1	0.1

hsa-miR-4798-5p	0.1	0.1	0.1	0.1
hsa-miR-4799-3p	0.1	0.1	0.1	0.1
hsa-miR-4799-5p	0.1	0.1	0.1	0.1
hsa-miR-4800-3p	0.1	0.1	0.1	0.1
hsa-miR-4800-5p	0.1	0.1	7.50369	0.1
hsa-miR-4801	0.1	0.1	0.1	0.1
hsa-miR-4802-3p	0.1	0.1	0.1	0.1
hsa-miR-4802-5p	0.1	0.1	0.1	0.1
hsa-miR-4803	0.1	0.1	0.1	0.1
hsa-miR-4804-3p	0.1	0.1	0.1	0.1
hsa-miR-4804-5p	0.1	0.1	0.1	0.1
hsa-miR-483-3p	0.1	0.1	0.1	1.61483
hsa-miR-483-5p	2.02164	3.1734	7.91017	1.84785
hsa-miR-484	6.01518	6.34769	11.2216	9.45559
hsa-miR-485-3p	0.1	0.1	0.1	0.1
hsa-miR-485-5p	0.1	0.1	0.1	0.1
hsa-miR-486-3p	0.1	0.1	0.1	0.1
hsa-miR-486-5p	0.1	0.1	0.1	0.1
hsa-miR-487a-3p	0.1	0.1	0.1	0.1
hsa-miR-487a-5p	0.1	0.1	0.1	0.1
hsa-miR-487b-3p	0.1	0.1	0.1	1.87659
hsa-miR-487b-5p	0.1	0.1	0.1	0.1
hsa-miR-488-3p	0.1	0.1	0.1	0.1
hsa-miR-488-5p	0.1	0.1	0.1	0.1
hsa-miR-489-3p	0.1	0.1	0.1	0.1
hsa-miR-489-5p	0.1	0.1	0.1	0.1
hsa-miR-490-3p	0.1	0.1	0.1	0.1
hsa-miR-490-5p	0.1	0.1	0.1	0.1
hsa-miR-491-3p	0.1	0.1	0.1	0.1
hsa-miR-491-5p	0.1	0.1	0.1	0.1
hsa-miR-492	0.1	0.1	0.1	0.1
hsa-miR-493-3p	0.1	0.1	0.1	0.1
hsa-miR-493-5p	0.1	0.1	0.1	0.1
hsa-miR-494-3p	167.306	183.048	162.34	170.648
hsa-miR-494-5p	0.1	0.1	0.1	0.1
hsa-miR-495-3p	0.1	0.1	0.1	2.03783
hsa-miR-495-5p	0.1	0.1	0.1	0.1
hsa-miR-496	0.1	0.1	0.1	0.1
hsa-miR-497-3p	0.1	0.1	0.1	0.1
hsa-miR-497-5p	0.1	0.1	0.1	0.1
hsa-miR-498	0.1	0.1	0.1	0.1
hsa-miR-4999-3p	0.1	0.1	0.1	0.1
hsa-miR-4999-5p	0.1	0.1	0.1	0.1
hsa-miR-499a-3p	0.1	0.1	0.1	0.1
hsa-miR-499a-5p	0.1	0.1	0.1	0.1
hsa-miR-499b-3p	0.1	0.1	0.1	0.1
hsa-miR-499b-5p	0.1	0.1	0.1	0.1
hsa-miR-5000-3p	0.1	0.1	0.1	0.1
hsa-miR-5000-5p	0.1	0.1	0.1	0.1
hsa-miR-5001-3p	0.1	0.1	0.1	0.1
hsa-miR-5001-5p	26.1111	22.4582	32.1354	18.4121
hsa-miR-5002-3p	0.1	0.1	0.1	0.1
hsa-miR-5002-5p	0.1	0.1	0.1	0.1

hsa-miR-5003-3p	0.1	0.1	0.1	0.1
hsa-miR-5003-5p	0.1	0.1	0.1	0.1
hsa-miR-5004-3p	0.1	0.1	0.1	0.1
hsa-miR-5004-5p	0.1	0.1	0.1	0.1
hsa-miR-5006-3p	0.1	0.1	0.1	0.1
hsa-miR-5006-5p	5.72018	5.65652	5.1686	4.04688
hsa-miR-5007-3p	0.1	0.1	0.1	0.1
hsa-miR-5007-5p	0.1	0.1	0.1	0.1
hsa-miR-5008-3p	0.1	0.1	0.1	0.1
hsa-miR-5008-5p	0.1	0.1	0.1	0.1
hsa-miR-5009-3p	0.1	0.1	0.1	0.1
hsa-miR-5009-5p	0.1	0.1	0.1	0.1
hsa-miR-500a-3p	0.1	0.1	0.1	0.1
hsa-miR-500a-5p	0.1	0.1	0.1	0.1
hsa-miR-500b-3p	0.1	0.1	0.1	0.1
hsa-miR-500b-5p	0.1	0.1	0.1	0.1
hsa-miR-5010-3p	0.1	0.1	0.1	0.1
hsa-miR-5010-5p	0.1	0.1	0.1	0.1
hsa-miR-5011-3p	0.1	0.1	0.1	0.1
hsa-miR-5011-5p	0.1	0.1	0.1	0.1
hsa-miR-501-3p	0.1	0.1	0.1	0.1
hsa-miR-501-5p	0.1	0.1	0.1	0.1
hsa-miR-502-3p	0.1	0.1	0.1	0.1
hsa-miR-502-5p	0.1	0.1	0.1	0.1
hsa-miR-503-3p	0.1	0.1	0.1	0.1
hsa-miR-503-5p	3.87002	23.5832	0.1	28.5039
hsa-miR-504-3p	0.1	0.1	0.1	0.1
hsa-miR-504-5p	0.1	0.1	0.1	0.1
hsa-miR-5047	0.1	0.1	0.1	0.1
hsa-miR-505-3p	1.76987	0.1	0.1	0.1
hsa-miR-505-5p	0.1	0.1	0.1	0.1
hsa-miR-506-3p	0.1	0.1	0.1	0.1
hsa-miR-506-5p	0.1	0.1	0.1	0.1
hsa-miR-507	0.1	0.1	0.1	0.1
hsa-miR-508-3p	0.1	0.1	0.1	0.1
hsa-miR-508-5p	0.1	0.1	0.1	0.1
hsa-miR-5087	0.1	0.1	0.1	0.1
hsa-miR-5088-3p	0.1	0.1	0.1	0.1
hsa-miR-5088-5p	0.1	0.1	0.1	0.1
hsa-miR-5089-3p	0.1	0.1	0.1	0.1
hsa-miR-5089-5p	0.1	0.1	0.1	0.1
hsa-miR-5090	0.1	0.1	0.1	0.1
hsa-miR-5091	0.1	0.1	0.1	0.1
hsa-miR-5092	0.1	0.1	0.1	0.1
hsa-miR-5093	0.1	0.1	0.1	0.1
hsa-miR-509-3-5p	0.1	0.1	0.1	0.1
hsa-miR-509-3p	0.1	0.1	0.1	0.1
hsa-miR-5094	0.1	0.1	0.1	0.1
hsa-miR-5095	0.1	0.1	0.1	0.1
hsa-miR-509-5p	0.1	0.1	0.1	0.1
hsa-miR-5096	0.1	0.1	0.1	0.1
hsa-miR-5100	204.934	648.982	170.116	1108.44
hsa-miR-510-3p	0.1	0.1	0.1	0.1

hsa-miR-510-5p	0.1	0.1	0.1	0.1
hsa-miR-511-3p	0.1	0.1	0.1	0.1
hsa-miR-511-5p	0.1	0.1	0.1	0.1
hsa-miR-512-3p	0.1	0.1	0.1	0.1
hsa-miR-512-5p	0.1	0.1	0.1	0.1
hsa-miR-513a-3p	0.1	0.1	0.1	0.1
hsa-miR-513a-5p	4.77834	2.82654	1.915	1.80526
hsa-miR-513b-3p	0.1	0.1	0.1	0.1
hsa-miR-513b-5p	1.46954	1.43079	1.15595	1.1109
hsa-miR-513c-3p	0.1	0.1	0.1	0.1
hsa-miR-513c-5p	0.1	0.1	0.1	0.1
hsa-miR-514a-3p	0.1	0.1	0.1	0.1
hsa-miR-514a-5p	0.1	0.1	0.1	0.1
hsa-miR-514b-3p	0.1	0.1	0.1	0.1
hsa-miR-514b-5p	0.1	0.1	0.1	0.1
hsa-miR-515-3p	0.1	0.1	0.1	0.1
hsa-miR-515-5p	0.1	0.1	0.1	0.1
hsa-miR-516a-3p	0.1	0.1	0.1	0.1
hsa-miR-516a-5p	0.1	0.1	0.1	0.1
hsa-miR-516b-5p	0.1	0.1	0.1	0.1
hsa-miR-517-5p	0.1	0.1	0.1	0.1
hsa-miR-517a-3p	0.1	0.1	0.1	0.1
hsa-miR-517c-3p	0.1	0.1	0.1	0.1
hsa-miR-5186	0.1	0.1	0.1	0.1
hsa-miR-5187-3p	0.1	0.1	0.1	0.1
hsa-miR-5187-5p	0.1	0.1	0.1	0.1
hsa-miR-5188	1.5752	1.8591	11.5498	0.1
hsa-miR-5189-3p	0.1	0.1	0.1	0.1
hsa-miR-5189-5p	0.1	0.1	0.1	0.1
hsa-miR-518a-3p	0.1	0.1	0.1	0.1
hsa-miR-518a-5p	0.1	0.1	0.1	0.1
hsa-miR-518b	0.1	0.1	0.1	0.1
hsa-miR-518c-3p	0.1	0.1	0.1	0.1
hsa-miR-518c-5p	0.1	0.1	0.1	0.1
hsa-miR-518d-3p	0.1	0.1	0.1	0.1
hsa-miR-518e-3p	0.1	0.1	0.1	0.1
hsa-miR-518e-5p	0.1	0.1	0.1	0.1
hsa-miR-518f-3p	0.1	0.1	0.1	0.1
hsa-miR-518f-5p	0.1	0.1	0.1	0.1
hsa-miR-5190	0.1	0.1	0.1	0.1
hsa-miR-5191	0.1	0.1	0.1	0.1
hsa-miR-5192	0.1	0.1	0.1	0.1
hsa-miR-5193	0.1	0.1	0.1	0.1
hsa-miR-5194	3.47877	1.77137	7.6738	3.54358
hsa-miR-5195-3p	0.1	0.1	0.1	0.1
hsa-miR-5195-5p	0.1	0.1	0.1	0.1
hsa-miR-5196-3p	0.1	0.1	0.1	0.1
hsa-miR-5196-5p	0.1	0.1	0.1	0.1
hsa-miR-5197-3p	0.1	0.1	0.1	0.1
hsa-miR-5197-5p	0.1	0.1	0.1	0.1
hsa-miR-519b-3p	0.1	0.1	0.1	0.1
hsa-miR-519c-3p	0.1	0.1	0.1	0.1
hsa-miR-519d-3p	0.1	0.1	0.1	0.1

hsa-miR-519d-5p	0.1	0.1	0.1	0.1
hsa-miR-519e-3p	0.1	0.1	0.1	0.1
hsa-miR-519e-5p	0.1	0.1	0.1	0.1
hsa-miR-520a-3p	0.1	0.1	0.1	0.1
hsa-miR-520a-5p	0.1	0.1	0.1	0.1
hsa-miR-520b	0.1	0.1	0.1	0.1
hsa-miR-520c-3p	0.1	0.1	0.1	0.1
hsa-miR-520d-3p	0.1	0.1	0.1	0.1
hsa-miR-520e	0.1	0.1	0.1	0.1
hsa-miR-520f-3p	0.1	0.1	0.1	0.1
hsa-miR-520f-5p	0.1	0.1	0.1	0.1
hsa-miR-520g-3p	0.1	0.1	0.1	0.1
hsa-miR-520g-5p	0.1	0.1	0.1	0.1
hsa-miR-520h	0.1	0.1	0.1	0.1
hsa-miR-521	0.1	0.1	0.1	0.1
hsa-miR-522-3p	0.1	0.1	0.1	0.1
hsa-miR-523-3p	0.1	0.1	0.1	0.1
hsa-miR-524-3p	0.1	0.1	0.1	0.1
hsa-miR-525-3p	0.1	0.1	0.1	0.1
hsa-miR-525-5p	0.1	0.1	0.1	0.1
hsa-miR-526b-3p	0.1	0.1	0.1	0.1
hsa-miR-526b-5p	0.1	0.1	0.1	0.1
hsa-miR-532-3p	0.1	0.1	0.1	0.1
hsa-miR-532-5p	2.01409	0.1	0.1	1.81855
hsa-miR-539-3p	0.1	0.1	0.1	0.1
hsa-miR-539-5p	0.1	0.1	0.1	0.1
hsa-miR-541-3p	0.1	0.1	0.1	0.1
hsa-miR-541-5p	0.1	0.1	0.1	0.1
hsa-miR-542-3p	0.1	2.84704	0.1	6.87496
hsa-miR-542-5p	0.1	0.1	0.1	2.11544
hsa-miR-543	0.1	0.1	0.1	0.1
hsa-miR-544a	0.1	0.1	0.1	0.1
hsa-miR-544b	0.1	0.1	0.1	0.1
hsa-miR-545-3p	0.1	0.1	0.1	0.1
hsa-miR-545-5p	0.1	0.1	0.1	0.1
hsa-miR-548a-3p	0.1	0.1	0.1	0.1
hsa-miR-548a-5p	0.1	0.1	0.1	0.1
hsa-miR-548aa	0.1	0.1	0.1	0.1
hsa-miR-548ab	0.1	0.1	0.1	0.1
hsa-miR-548ac	0.1	0.1	0.1	0.1
hsa-miR-548ad-3p	0.1	0.1	0.1	0.1
hsa-miR-548ad-5p	0.1	0.1	0.1	0.1
hsa-miR-548ae-3p	0.1	0.1	0.1	0.1
hsa-miR-548ag	0.1	0.1	0.1	0.1
hsa-miR-548ah-5p	0.1	0.1	0.1	0.1
hsa-miR-548ai	0.1	0.1	0.1	0.1
hsa-miR-548aj-3p	0.1	0.1	0.1	0.1
hsa-miR-548aj-5p	0.1	0.1	0.1	0.1
hsa-miR-548ak	0.1	0.1	0.1	0.1
hsa-miR-548al	0.1	0.1	0.1	0.1
hsa-miR-548am-3p	0.1	0.1	0.1	0.1
hsa-miR-548am-5p	0.1	0.1	0.1	0.1
hsa-miR-548an	0.1	0.1	0.1	0.1

hsa-miR-548ao-3p	0.1	0.1	0.1	0.1
hsa-miR-548ao-5p	0.1	0.1	0.1	0.1
hsa-miR-548ap-3p	0.1	0.1	0.1	0.1
hsa-miR-548ap-5p	0.1	0.1	0.1	0.1
hsa-miR-548aq-5p	0.1	0.1	0.1	0.1
hsa-miR-548ar-3p	0.1	0.1	0.1	0.1
hsa-miR-548ar-5p	0.1	0.1	0.1	0.1
hsa-miR-548as-3p	0.1	0.1	0.1	0.1
hsa-miR-548as-5p	0.1	0.1	0.1	0.1
hsa-miR-548at-3p	0.1	0.1	0.1	0.1
hsa-miR-548at-5p	0.1	0.1	0.1	0.1
hsa-miR-548au-3p	0.1	0.1	0.1	0.1
hsa-miR-548au-5p	0.1	0.1	0.1	0.1
hsa-miR-548av-3p	0.1	0.1	0.1	0.1
hsa-miR-548av-5p	0.1	0.1	0.1	0.1
hsa-miR-548aw	0.1	0.1	0.1	0.1
hsa-miR-548ax	0.1	0.1	0.1	0.1
hsa-miR-548ay-3p	0.1	0.1	0.1	0.1
hsa-miR-548ay-5p	0.1	0.1	0.1	0.1
hsa-miR-548az-3p	0.1	0.1	0.1	0.1
hsa-miR-548az-5p	0.1	0.1	0.1	0.1
hsa-miR-548b-3p	0.1	0.1	0.1	0.1
hsa-miR-548b-5p	0.1	0.1	0.1	0.1
hsa-miR-548ba	0.1	0.1	0.1	0.1
hsa-miR-548bb-3p	0.1	0.1	0.1	0.1
hsa-miR-548bb-5p	0.1	0.1	0.1	0.1
hsa-miR-548c-3p	0.1	0.945076	0.1	0.1
hsa-miR-548d-3p	0.1	0.1	0.1	0.1
hsa-miR-548d-5p	0.1	0.1	0.1	0.1
hsa-miR-548e-3p	0.1	0.1	0.1	0.1
hsa-miR-548e-5p	0.1	0.1	0.1	0.1
hsa-miR-548f-3p	0.1	0.1	0.1	0.1
hsa-miR-548f-5p	0.1	0.1	0.1	0.1
hsa-miR-548g-3p	0.1	0.1	0.1	0.1
hsa-miR-548h-3p	0.1	0.1	0.1	0.1
hsa-miR-548h-5p	0.1	0.1	0.1	0.1
hsa-miR-548i	0.1	0.1	0.1	0.1
hsa-miR-548j-3p	0.1	0.1	0.1	0.1
hsa-miR-548j-5p	0.1	0.1	0.1	0.1
hsa-miR-548k	0.1	0.1	0.1	0.1
hsa-miR-548l	0.1	0.1	0.1	0.1
hsa-miR-548m	0.1	0.1	0.1	0.1
hsa-miR-548n	0.1	0.1	0.1	0.1
hsa-miR-548p	0.1	0.1	0.1	0.1
hsa-miR-548q	0.1	0.1	0.1	0.1
hsa-miR-548s	0.1	0.1	0.1	0.1
hsa-miR-548t-5p	0.1	0.1	0.1	0.1
hsa-miR-548u	0.1	0.1	0.1	0.1
hsa-miR-548v	0.1	0.1	0.1	0.1
hsa-miR-548w	0.1	0.1	0.1	0.1
hsa-miR-548x-3p	0.1	0.1	0.1	0.1
hsa-miR-548y	0.1	0.1	0.1	0.1
hsa-miR-549a	0.1	0.1	0.1	0.1

hsa-miR-550a-3-5p	0.1	0.1	0.1	0.1
hsa-miR-550a-3p	5.44168	5.08239	3.03806	3.68621
hsa-miR-550a-5p	0.1	0.1	0.1	0.1
hsa-miR-550b-2-5p	0.1	0.1	0.1	0.1
hsa-miR-550b-3p	0.1	0.1	0.1	0.1
hsa-miR-551a	0.1	0.1	0.1	0.1
hsa-miR-551b-3p	0.1	0.1	0.1	0.1
hsa-miR-551b-5p	0.1	0.1	0.1	0.1
hsa-miR-552-3p	0.1	0.1	0.1	0.1
hsa-miR-552-5p	0.1	0.1	0.1	0.1
hsa-miR-553	0.1	0.1	0.1	0.1
hsa-miR-554	0.1	0.1	0.1	0.1
hsa-miR-555	0.1	0.1	0.1	0.1
hsa-miR-556-3p	0.1	0.1	0.1	0.1
hsa-miR-556-5p	0.1	0.1	0.1	0.1
hsa-miR-557	0.1	0.1	0.1	0.1
hsa-miR-5571-3p	0.1	0.1	0.1	0.1
hsa-miR-5571-5p	0.1	0.1	0.1	0.1
hsa-miR-5572	0.1	0.1	0.1	0.1
hsa-miR-5579-3p	0.1	0.1	0.1	0.1
hsa-miR-5579-5p	0.1	0.1	0.1	0.1
hsa-miR-558	0.1	0.1	0.1	0.1
hsa-miR-5580-3p	0.1	0.1	0.1	0.1
hsa-miR-5580-5p	0.1	0.1	0.1	0.1
hsa-miR-5581-3p	0.1	0.1	0.1	0.1
hsa-miR-5581-5p	4.68948	4.49703	4.59388	5.61856
hsa-miR-5582-3p	0.1	0.1	0.1	0.1
hsa-miR-5582-5p	0.1	0.1	0.1	0.1
hsa-miR-5583-3p	0.1	0.1	0.1	0.1
hsa-miR-5583-5p	0.1	0.1	0.1	0.1
hsa-miR-5584-3p	0.1	0.1	0.1	0.1
hsa-miR-5584-5p	0.1	0.1	0.1	0.1
hsa-miR-5585-3p	1.88423	0.1	0.1	0.1
hsa-miR-5585-5p	0.1	0.1	0.1	0.1
hsa-miR-5586-3p	0.1	0.1	0.1	0.1
hsa-miR-5586-5p	0.1	0.1	0.1	0.1
hsa-miR-5587-3p	0.1	0.1	0.1	0.1
hsa-miR-5587-5p	0.1	0.1	0.1	0.1
hsa-miR-5588-3p	0.1	0.1	0.1	0.1
hsa-miR-5588-5p	0.1	0.1	0.1	0.1
hsa-miR-5589-3p	0.1	0.1	0.1	0.1
hsa-miR-5589-5p	0.1	0.1	0.1	0.1
hsa-miR-559	0.1	0.1	0.1	0.1
hsa-miR-5590-3p	0.1	0.1	0.1	0.1
hsa-miR-5590-5p	0.1	0.1	0.1	0.1
hsa-miR-5591-3p	0.1	0.1	0.1	0.1
hsa-miR-5591-5p	0.1	0.1	0.1	0.1
hsa-miR-561-3p	0.1	0.1	0.1	0.1
hsa-miR-561-5p	0.1	0.1	0.1	0.1
hsa-miR-562	0.1	0.1	0.1	0.1
hsa-miR-563	0.1	0.1	0.1	0.1
hsa-miR-564	0.1	0.1	1.14914	0.1
hsa-miR-566	0.1	0.1	0.1	0.1

hsa-miR-567	0.1	0.1	0.1	0.1
hsa-miR-568	0.1	0.1	0.1	0.1
hsa-miR-5680	0.1	0.1	0.1	0.1
hsa-miR-5681a	0.1	0.1	0.1	0.1
hsa-miR-5681b	0.1	0.1	0.1	0.1
hsa-miR-5682	0.1	0.1	0.1	0.1
hsa-miR-5683	0.1	0.1	0.1	0.1
hsa-miR-5684	0.1	0.1	2.83431	0.1
hsa-miR-5685	0.1	0.1	0.1	0.1
hsa-miR-5687	0.1	0.1	0.1	0.1
hsa-miR-5688	0.1	0.1	0.1	0.1
hsa-miR-5689	0.1	0.1	0.1	0.1
hsa-miR-569	0.1	0.1	0.1	0.1
hsa-miR-5690	0.1	0.1	0.1	0.1
hsa-miR-5691	0.1	0.1	0.1	0.1
hsa-miR-5692a	0.1	0.1	0.1	0.1
hsa-miR-5692b	0.1	0.1	0.1	0.1
hsa-miR-5692c	0.1	0.1	0.1	0.1
hsa-miR-5693	0.1	0.1	0.1	0.1
hsa-miR-5694	0.1	0.1	0.1	0.1
hsa-miR-5695	0.1	0.1	0.1	0.1
hsa-miR-5696	0.1	0.1	0.1	0.1
hsa-miR-5697	0.1	0.1	0.1	0.1
hsa-miR-5698	0.1	0.1	0.1	0.1
hsa-miR-5699-3p	0.1	0.1	0.1	0.1
hsa-miR-5699-5p	0.1	0.1	0.1	0.1
hsa-miR-5700	0.1	0.1	0.1	0.1
hsa-miR-5701	0.1	0.1	0.1	0.1
hsa-miR-5702	0.1	0.1	0.1	0.1
hsa-miR-5703	0.1	19.1952	28.6958	10.5272
hsa-miR-570-3p	0.1	0.1	0.1	0.1
hsa-miR-5704	0.1	0.1	0.1	0.1
hsa-miR-5705	0.1	0.1	0.1	0.1
hsa-miR-5706	0.1	0.1	0.1	0.1
hsa-miR-5707	0.1	0.1	0.1	0.1
hsa-miR-5708	0.1	0.1	0.1	0.1
hsa-miR-571	3.33667	0.1	0.1	0.1
hsa-miR-572	8.1385	8.27918	10.3478	7.87671
hsa-miR-573	0.1	0.1	0.1	0.1
hsa-miR-5739	52.766	75.61	83.4647	89.115
hsa-miR-574-3p	0.1	0.1	0.1	0.1
hsa-miR-574-5p	9.70208	13.2147	14.5463	12.1607
hsa-miR-575	5.46836	9.19842	6.79499	7.31572
hsa-miR-576-3p	0.1	0.1	0.1	0.1
hsa-miR-576-5p	0.1	0.1	0.1	0.1
hsa-miR-577	0.1	0.1	0.1	0.1
hsa-miR-578	0.1	0.1	0.1	0.1
hsa-miR-5787	3.98055	14.0863	22.9307	7.28632
hsa-miR-579-3p	0.1	0.1	0.1	0.1
hsa-miR-579-5p	0.1	0.1	0.1	0.1
hsa-miR-580-3p	0.1	0.1	0.1	0.1
hsa-miR-580-5p	0.1	0.1	0.1	0.1
hsa-miR-581	0.1	0.1	0.1	0.1

hsa-miR-582-3p	0.1	1.2672	0.1	1.06934
hsa-miR-582-5p	32.471	58.5791	14.3979	54.2365
hsa-miR-583	0.1	0.1	0.1	0.1
hsa-miR-584-3p	0.1	0.1	0.1	0.1
hsa-miR-584-5p	0.1	0.1	5.3346	0.1
hsa-miR-585-3p	0.1	0.1	0.1	0.1
hsa-miR-585-5p	0.1	0.1	0.1	0.1
hsa-miR-586	0.1	0.1	0.1	0.1
hsa-miR-587	0.1	0.1	0.1	0.1
hsa-miR-588	0.1	0.1	0.1	0.1
hsa-miR-589-3p	0.1	0.1	0.1	0.1
hsa-miR-589-5p	0.1	0.1	0.1	0.1
hsa-miR-590-3p	0.1	0.1	0.1	0.1
hsa-miR-590-5p	8.90021	7.86356	4.78255	9.03291
hsa-miR-591	0.1	0.1	0.1	0.1
hsa-miR-592	0.1	0.1	0.1	0.1
hsa-miR-593-3p	0.1	0.1	0.1	0.1
hsa-miR-593-5p	0.1	0.1	0.1	0.1
hsa-miR-595	0.1	0.1	0.1	0.1
hsa-miR-596	0.1	0.1	0.1	0.1
hsa-miR-597-3p	0.1	0.1	0.1	0.1
hsa-miR-597-5p	0.1	0.1	0.1	0.1
hsa-miR-598-3p	0.1	0.1	0.1	0.1
hsa-miR-598-5p	0.1	0.1	0.1	0.1
hsa-miR-599	0.1	0.1	0.1	0.1
hsa-miR-600	0.1	0.1	0.1	0.1
hsa-miR-601	0.1	0.1	0.1	0.1
hsa-miR-602	0.1	0.1	0.1	0.1
hsa-miR-603	0.1	0.1	0.1	0.1
hsa-miR-604	0.1	0.1	0.1	0.1
hsa-miR-605-3p	0.1	0.1	0.1	0.1
hsa-miR-605-5p	0.1	0.1	0.1	0.1
hsa-miR-606	0.1	0.1	0.1	0.1
hsa-miR-6068	18.0932	17.7981	23.4747	15.1915
hsa-miR-6069	0.1	0.1	0.1	0.1
hsa-miR-607	0.1	0.1	0.1	0.1
hsa-miR-6070	0.1	0.1	0.1	0.1
hsa-miR-6071	0.1	0.1	0.1	0.1
hsa-miR-6072	0.1	0.1	0.1	0.1
hsa-miR-6073	0.1	0.1	0.1	0.1
hsa-miR-6074	0.1	0.1	0.1	0.1
hsa-miR-6075	0.1	0.1	0.1	0.1
hsa-miR-6076	0.1	0.1	6.00934	0.1
hsa-miR-6077	0.1	0.1	0.1	0.1
hsa-miR-6078	0.1	0.1	0.1	0.1
hsa-miR-6079	0.1	0.1	0.1	0.1
hsa-miR-608	0.1	0.1	0.1	0.1
hsa-miR-6080	0.1	0.1	0.1	0.1
hsa-miR-6081	0.1	0.1	0.1	0.1
hsa-miR-6082	0.1	0.1	0.1	0.1
hsa-miR-6083	0.1	0.1	0.1	0.1
hsa-miR-6084	0.1	0.1	0.1	0.1
hsa-miR-6085	28.7686	52.7304	52.6528	64.9587

hsa-miR-6086	0.1	0.1	0.1	0.1
hsa-miR-6087	145.568	131.632	190.81	102.637
hsa-miR-6088	38.1516	34.4155	43.7807	25.3721
hsa-miR-6089	148.095	162.352	156.172	127.75
hsa-miR-609	0.1	0.1	0.1	0.1
hsa-miR-6090	65.8778	64.9762	50.9652	44.1193
hsa-miR-610	0.1	0.1	0.1	0.1
hsa-miR-611	0.1	0.1	0.1	0.1
hsa-miR-612	0.1	0.1	0.1	0.1
hsa-miR-6124	8.20401	9.16464	9.71086	7.64311
hsa-miR-6125	71.0958	73.2403	79.0395	63.1061
hsa-miR-6126	3.0884	3.29037	3.65924	2.05691
hsa-miR-6127	64.3912	69.5292	58.7127	78.1359
hsa-miR-6128	0.1	0.1	0.1	0.1
hsa-miR-6129	0.1	0.1	0.1	0.1
hsa-miR-613	0.1	0.1	0.1	0.1
hsa-miR-6130	0.1	0.1	0.1	0.1
hsa-miR-6131	10.5961	11.1303	9.71072	12.7818
hsa-miR-6132	15.9411	18.5858	16.1878	17.1183
hsa-miR-6133	0.1	0.1	0.1	0.1
hsa-miR-6134	0.1	0.1	0.1	0.1
hsa-miR-614	0.1	0.1	0.1	0.1
hsa-miR-615-3p	0.1	0.1	0.1	0.1
hsa-miR-615-5p	0.1	0.1	0.1	0.1
hsa-miR-616-3p	0.1	0.1	0.1	0.1
hsa-miR-6165	23.301	37.4482	42.3481	45.9841
hsa-miR-616-5p	0.1	0.1	0.1	0.1
hsa-miR-617	0.1	0.1	0.1	0.1
hsa-miR-618	0.1	0.1	0.1	0.1
hsa-miR-619-3p	0.1	0.1	0.1	0.1
hsa-miR-619-5p	0.1	0.1	0.1	0.1
hsa-miR-620	0.1	0.1	0.1	0.1
hsa-miR-621	0.1	0.1	0.1	0.1
hsa-miR-622	0.1	0.1	0.1	0.1
hsa-miR-623	0.1	0.1	0.1	0.1
hsa-miR-624-3p	0.1	0.1	0.1	0.1
hsa-miR-624-5p	0.1	0.1	0.1	0.1
hsa-miR-625-3p	0.1	0.1	0.1	0.1
hsa-miR-625-5p	0.1	0.1	0.1	0.1
hsa-miR-626	0.1	0.1	0.1	0.1
hsa-miR-627-3p	0.1	0.1	0.1	0.1
hsa-miR-627-5p	0.1	0.1	0.1	0.1
hsa-miR-628-3p	0.1	0.1	0.1	0.1
hsa-miR-628-5p	0.1	0.1	0.1	0.1
hsa-miR-629-3p	0.1	0.1	0.1	0.1
hsa-miR-629-5p	0.843699	0.1	0.1	0.1
hsa-miR-630	4.2459	33.1959	46.3747	18.3226
hsa-miR-631	0.1	0.1	0.1	0.1
hsa-miR-632	0.1	0.1	0.1	0.1
hsa-miR-633	0.1	0.1	0.1	0.1
hsa-miR-634	0.1	0.1	0.1	0.1
hsa-miR-635	0.1	0.1	0.1	0.1
hsa-miR-636	0.1	0.1	0.1	0.1

hsa-miR-637	0.1	0.1	0.1	0.1
hsa-miR-638	32.2143	36.8139	44.0704	33.7565
hsa-miR-639	0.1	0.1	0.1	0.1
hsa-miR-640	0.1	0.1	0.1	0.1
hsa-miR-641	0.1	0.1	0.1	0.1
hsa-miR-642a-3p	29.4801	34.1902	40.9389	25.8886
hsa-miR-642a-5p	0.1	0.1	0.1	0.1
hsa-miR-642b-3p	8.81119	9.20228	7.61081	6.3874
hsa-miR-642b-5p	0.1	0.1	0.1	0.1
hsa-miR-643	0.1	0.1	0.1	0.1
hsa-miR-644a	0.1	0.1	0.1	0.1
hsa-miR-645	0.1	0.1	0.1	0.1
hsa-miR-646	0.1	0.1	0.1	0.1
hsa-miR-647	0.1	0.1	0.1	0.1
hsa-miR-648	0.1	0.1	0.1	0.1
hsa-miR-649	0.1	0.1	0.1	0.1
hsa-miR-6499-3p	0.1	0.1	0.1	0.1
hsa-miR-6499-5p	0.1	0.1	0.1	0.1
hsa-miR-650	0.1	0.1	0.1	0.1
hsa-miR-6500-3p	0.1	0.1	0.1	0.1
hsa-miR-6500-5p	0.1	0.1	0.1	0.1
hsa-miR-6501-3p	0.1	0.1	0.1	0.1
hsa-miR-6501-5p	0.1	0.1	0.1	0.1
hsa-miR-6502-3p	0.1	0.1	0.1	0.1
hsa-miR-6502-5p	0.1	0.1	0.1	0.1
hsa-miR-6503-3p	0.1	0.1	0.1	0.1
hsa-miR-6503-5p	0.1	0.1	0.1	0.1
hsa-miR-6504-3p	0.1	0.1	0.1	0.1
hsa-miR-6504-5p	0.1	0.1	0.1	0.1
hsa-miR-6505-3p	0.1	0.1	0.1	0.1
hsa-miR-6505-5p	0.1	0.1	0.1	0.1
hsa-miR-6506-3p	0.1	0.1	0.1	0.1
hsa-miR-6506-5p	0.1	0.1	0.1	0.1
hsa-miR-6507-3p	1.60529	0.1	0.1	0.1
hsa-miR-6507-5p	0.1	0.1	0.1	0.1
hsa-miR-6508-3p	0.1	0.1	0.1	0.1
hsa-miR-6508-5p	0.1	0.1	0.1	0.1
hsa-miR-6509-3p	0.1	0.1	0.1	0.1
hsa-miR-6509-5p	0.1	0.1	0.1	0.1
hsa-miR-6510-3p	0.1	0.1	0.1	0.1
hsa-miR-6510-5p	2.35477	5.46326	2.58406	2.49827
hsa-miR-6511a-3p	0.1	0.1	0.1	0.1
hsa-miR-6511a-5p	0.1	0.1	0.1	0.1
hsa-miR-6511b-3p	0.1	0.1	0.1	0.1
hsa-miR-6511b-5p	0.1	0.1	0.1	0.1
hsa-miR-6512-3p	0.1	0.1	0.1	0.1
hsa-miR-6512-5p	0.923357	0.1	0.1	0.1
hsa-miR-6513-3p	0.1	0.1	0.1	0.1
hsa-miR-6513-5p	0.1	0.1	0.1	0.1
hsa-miR-651-3p	0.1	0.1	0.1	0.1
hsa-miR-6514-3p	0.1	0.1	0.1	0.1
hsa-miR-6514-5p	0.1	0.1	0.1	0.1
hsa-miR-6515-3p	0.1	0.1	0.1	0.1

hsa-miR-6515-5p	0.1	0.1	0.1	0.1
hsa-miR-651-5p	0.1	0.1	0.1	0.1
hsa-miR-6516-3p	4.56841	3.86222	4.0593	3.31475
hsa-miR-6516-5p	0.1	0.1	0.1	0.1
hsa-miR-652-3p	3.08734	1.91349	2.84721	2.28693
hsa-miR-652-5p	0.1	0.1	0.1	0.1
hsa-miR-653-3p	0.1	0.1	0.1	0.1
hsa-miR-653-5p	0.1	0.1	0.1	0.1
hsa-miR-654-3p	0.1	2.19932	0.1	2.58348
hsa-miR-654-5p	0.1	0.1	0.1	0.1
hsa-miR-655-3p	0.1	0.1	0.1	0.1
hsa-miR-655-5p	0.1	0.1	0.1	0.1
hsa-miR-656-3p	0.1	0.1	0.1	0.1
hsa-miR-656-5p	0.1	0.1	0.1	0.1
hsa-miR-657	0.1	0.1	0.1	0.1
hsa-miR-658	0.1	0.1	1.94301	0.1
hsa-miR-659-3p	0.1	0.1	0.1	0.1
hsa-miR-659-5p	0.1	0.1	0.1	0.1
hsa-miR-660-3p	0.1	0.1	0.1	0.1
hsa-miR-660-5p	3.10239	2.69887	3.06968	3.76723
hsa-miR-661	0.1	0.1	0.1	0.1
hsa-miR-662	0.1	0.1	0.1	0.1
hsa-miR-663a	0.1	0.1	0.1	0.1
hsa-miR-663b	0.1	0.1	0.1	0.1
hsa-miR-664a-3p	0.1	0.1	0.1	0.1
hsa-miR-664a-5p	0.1	0.1	0.1	0.1
hsa-miR-664b-3p	4.23697	5.52005	5.17148	4.59675
hsa-miR-664b-5p	0.1	0.1	0.1	0.1
hsa-miR-665	0.1	0.1	0.1	0.1
hsa-miR-668-3p	0.1	0.1	0.1	0.1
hsa-miR-668-5p	0.1	0.1	0.1	0.1
hsa-miR-670-3p	0.1	0.1	0.1	0.1
hsa-miR-670-5p	0.1	0.1	0.1	0.1
hsa-miR-671-3p	0.1	0.1	0.1	0.1
hsa-miR-6715a-3p	0.1	0.1	0.1	0.1
hsa-miR-6715b-3p	0.1	0.1	0.1	0.1
hsa-miR-6715b-5p	0.1	0.1	0.1	0.1
hsa-miR-671-5p	0.1	0.1	0.1	0.1
hsa-miR-6716-3p	0.1	4.73873	0.1	1.62169
hsa-miR-6716-5p	0.1	0.1	0.1	0.1
hsa-miR-6717-5p	15.117	13.8164	14.1111	16.1716
hsa-miR-6718-5p	0.1	0.1	0.1	0.1
hsa-miR-6719-3p	0.1	0.1	0.1	0.1
hsa-miR-6720-3p	0.1	0.1	0.1	0.1
hsa-miR-6720-5p	0.1	0.1	0.1	0.1
hsa-miR-6721-5p	0.1	0.1	0.1	0.1
hsa-miR-6722-3p	0.1	0.1	0.1	0.1
hsa-miR-6722-5p	0.1	0.1	0.1	0.1
hsa-miR-6723-5p	0.1	0.1	0.1	0.1
hsa-miR-6724-5p	7.96921	7.89748	8.52049	5.79416
hsa-miR-6726-3p	0.1	0.1	0.1	0.1
hsa-miR-6726-5p	0.1	0.1	3.06777	0.1
hsa-miR-6727-3p	0.1	0.1	0.1	0.1

hsa-miR-6727-5p	0.1	0.1	0.1	0.1
hsa-miR-6728-3p	0.1	0.1	0.1	0.1
hsa-miR-6728-5p	10.5431	7.94095	12.9309	4.63499
hsa-miR-6729-3p	0.1	0.1	0.1	0.1
hsa-miR-6729-5p	0.1	0.1	0.1	0.1
hsa-miR-6730-3p	0.1	0.1	0.1	0.1
hsa-miR-6730-5p	0.1	0.1	0.1	0.1
hsa-miR-6731-3p	0.1	0.1	0.1	0.1
hsa-miR-6731-5p	0.1	0.1	0.1	0.1
hsa-miR-6732-3p	0.1	0.1	0.1	0.1
hsa-miR-6732-5p	0.1	0.1	0.1	0.1
hsa-miR-6733-3p	0.1	0.1	0.1	0.1
hsa-miR-6733-5p	0.1	0.1	0.1	0.1
hsa-miR-6734-3p	0.1	0.1	0.1	0.1
hsa-miR-6734-5p	6.60829	6.6605	6.29247	6.69222
hsa-miR-6735-3p	0.1	0.1	0.1	0.1
hsa-miR-6735-5p	0.1	0.1	0.1	0.1
hsa-miR-6736-3p	0.1	0.1	0.1	0.1
hsa-miR-6736-5p	0.1	0.1	0.1	0.1
hsa-miR-6737-3p	0.1	0.1	0.1	0.1
hsa-miR-6737-5p	0.1	0.1	0.1	0.1
hsa-miR-6738-3p	0.1	0.1	0.1	0.1
hsa-miR-6738-5p	0.1	0.1	0.1	0.1
hsa-miR-6739-3p	0.1	0.1	0.1	0.1
hsa-miR-6739-5p	0.1	0.1	0.1	0.1
hsa-miR-6740-3p	0.1	0.1	0.1	0.1
hsa-miR-6740-5p	19.7704	20.3379	18.7383	21.0278
hsa-miR-6741-3p	0.1	0.1	0.1	0.1
hsa-miR-6741-5p	0.1	0.1	0.1	0.1
hsa-miR-6742-3p	0.1	0.1	0.1	0.1
hsa-miR-6742-5p	0.1	0.1	0.1	0.1
hsa-miR-6743-3p	0.1	0.1	0.1	0.1
hsa-miR-6743-5p	0.1	0.1	0.1	0.1
hsa-miR-6744-3p	0.1	0.1	0.1	0.1
hsa-miR-6744-5p	0.1	0.1	0.1	0.1
hsa-miR-6745	0.1	0.1	0.1	0.1
hsa-miR-6746-3p	0.1	0.1	0.1	0.1
hsa-miR-6746-5p	0.1	0.1	0.1	0.1
hsa-miR-6747-3p	0.1	0.1	0.1	0.1
hsa-miR-6747-5p	0.1	0.1	0.1	0.1
hsa-miR-6748-3p	0.1	0.1	0.1	0.1
hsa-miR-6748-5p	0.1	0.1	0.1	0.1
hsa-miR-6749-3p	0.1	0.1	0.1	0.1
hsa-miR-6749-5p	23.5086	31.4197	34.7311	32.6838
hsa-miR-6750-3p	0.1	0.1	0.1	0.1
hsa-miR-6750-5p	0.1	0.1	0.1	0.1
hsa-miR-6751-3p	0.1	0.1	0.1	0.1
hsa-miR-6751-5p	0.1	0.1	0.1	0.1
hsa-miR-6752-3p	0.1	0.1	0.1	0.1
hsa-miR-6752-5p	1.8281	0.1	2.05327	0.1
hsa-miR-6753-3p	0.1	0.1	0.1	0.1
hsa-miR-6753-5p	0.1	0.1	0.1	0.1
hsa-miR-675-3p	0.1	0.1	0.1	0.1

hsa-miR-6754-3p	0.1	0.1	0.1	0.1
hsa-miR-6754-5p	0.1	0.1	0.1	0.1
hsa-miR-6755-3p	0.1	0.1	0.1	0.1
hsa-miR-6755-5p	0.1	0.1	0.1	0.1
hsa-miR-675-5p	0.1	0.1	0.1	0.1
hsa-miR-6756-3p	0.1	0.1	0.1	0.1
hsa-miR-6756-5p	0.1	0.1	0.1	0.1
hsa-miR-6757-3p	0.1	0.1	0.1	0.1
hsa-miR-6757-5p	0.1	0.1	2.1924	0.1
hsa-miR-6758-3p	0.1	0.1	0.1	0.1
hsa-miR-6758-5p	0.1	0.1	0.1	0.1
hsa-miR-6759-3p	0.1	0.1	0.1	0.1
hsa-miR-6759-5p	0.1	0.1	0.1	0.1
hsa-miR-6760-3p	0.1	0.1	0.1	0.1
hsa-miR-6760-5p	0.1	0.1	0.1	0.1
hsa-miR-6761-3p	0.1	0.1	0.1	0.1
hsa-miR-6761-5p	0.1	0.1	0.1	0.1
hsa-miR-6762-3p	0.1	0.1	0.1	0.1
hsa-miR-6762-5p	0.1	0.1	0.1	0.1
hsa-miR-6763-3p	0.1	0.1	0.1	0.1
hsa-miR-6763-5p	10.1176	12.9018	15.2572	14.595
hsa-miR-676-3p	0.1	0.1	0.1	0.1
hsa-miR-6764-3p	0.1	0.1	0.1	0.1
hsa-miR-6764-5p	0.1	0.1	0.1	0.1
hsa-miR-6765-3p	0.1	0.1	0.1	0.1
hsa-miR-6765-5p	0.1	0.1	0.1	0.1
hsa-miR-676-5p	0.1	0.1	0.1	0.1
hsa-miR-6766-3p	0.1	0.1	0.1	0.1
hsa-miR-6766-5p	0.1	0.1	0.1	0.1
hsa-miR-6767-3p	0.1	0.1	0.1	0.1
hsa-miR-6767-5p	5.1794	5.49848	5.32431	5.31304
hsa-miR-6768-3p	0.1	0.1	0.1	0.1
hsa-miR-6768-5p	0.1	0.1	0.1	0.1
hsa-miR-6769a-3p	0.1	0.1	0.1	0.1
hsa-miR-6769a-5p	0.1	0.1	0.1	0.1
hsa-miR-6769b-3p	0.1	0.1	0.1	0.1
hsa-miR-6769b-5p	5.08466	6.70452	5.97443	5.42931
hsa-miR-6770-3p	0.1	0.1	0.1	0.1
hsa-miR-6770-5p	0.1	0.1	0.1	0.1
hsa-miR-6771-3p	0.1	0.1	0.1	0.1
hsa-miR-6771-5p	0.1	0.1	0.1	0.1
hsa-miR-6772-3p	0.1	0.1	0.1	0.1
hsa-miR-6772-5p	0.1	0.1	0.1	0.1
hsa-miR-6773-3p	0.1	0.1	0.1	0.1
hsa-miR-6773-5p	0.1	0.1	0.1	0.1
hsa-miR-6774-3p	0.1	0.1	0.1	0.1
hsa-miR-6774-5p	0.1	0.1	0.1	0.1
hsa-miR-6775-3p	0.1	0.1	0.1	0.1
hsa-miR-6775-5p	0.1	0.1	2.3206	0.1
hsa-miR-6776-3p	0.1	0.1	0.1	0.1
hsa-miR-6776-5p	0.1	0.1	0.1	0.1
hsa-miR-6777-3p	0.1	0.1	0.1	0.1
hsa-miR-6777-5p	0.1	0.1	0.1	0.1

hsa-miR-6778-3p	0.1	0.1	0.1	0.1
hsa-miR-6778-5p	0.1	0.1	0.1	0.1
hsa-miR-6779-3p	0.1	0.1	0.1	0.1
hsa-miR-6779-5p	2.48685	2.45796	3.55488	1.65858
hsa-miR-6780a-3p	0.1	0.1	0.1	0.1
hsa-miR-6780a-5p	0.1	0.1	0.1	0.1
hsa-miR-6780b-3p	0.1	0.1	0.1	0.1
hsa-miR-6780b-5p	25.2396	25.9106	24.2791	25.9952
hsa-miR-6781-3p	0.1	0.1	0.1	0.1
hsa-miR-6781-5p	0.1	0.1	0.1	0.1
hsa-miR-6782-3p	0.1	0.1	0.1	0.1
hsa-miR-6782-5p	0.1	0.1	0.1	0.1
hsa-miR-6783-3p	0.1	0.1	0.1	0.1
hsa-miR-6783-5p	0.1	0.1	0.1	0.1
hsa-miR-6784-3p	0.1	0.1	0.1	0.1
hsa-miR-6784-5p	0.1	0.1	0.1	0.1
hsa-miR-6785-3p	0.1	0.1	0.1	0.1
hsa-miR-6785-5p	27.4724	32.4484	34.8682	28.4949
hsa-miR-6786-3p	0.1	0.1	0.1	0.1
hsa-miR-6786-5p	0.1	0.1	0.1	0.1
hsa-miR-6787-3p	0.1	0.1	0.1	0.1
hsa-miR-6787-5p	0.1	0.1	0.1	0.1
hsa-miR-6788-3p	0.1	0.1	0.1	0.1
hsa-miR-6788-5p	0.1	0.1	0.1	0.1
hsa-miR-6789-3p	0.1	0.1	0.1	0.1
hsa-miR-6789-5p	1.4953	0.1	0.1	0.1
hsa-miR-6790-3p	0.1	0.1	0.1	0.1
hsa-miR-6790-5p	0.1	0.1	0.1	0.1
hsa-miR-6791-3p	0.1	0.1	0.1	0.1
hsa-miR-6791-5p	8.33571	7.08774	9.54158	5.15713
hsa-miR-6792-3p	0.1	0.1	0.1	0.1
hsa-miR-6792-5p	0.1	0.1	0.1	0.1
hsa-miR-6793-3p	0.1	0.1	0.1	0.1
hsa-miR-6793-5p	0.1	0.1	0.1	0.1
hsa-miR-6794-3p	0.1	0.1	0.1	0.1
hsa-miR-6794-5p	0.1	0.1	0.1	0.1
hsa-miR-6795-3p	0.1	0.1	0.1	0.1
hsa-miR-6795-5p	0.1	0.1	0.1	0.1
hsa-miR-6796-3p	0.1	0.1	0.1	0.1
hsa-miR-6796-5p	0.1	0.1	0.1	0.1
hsa-miR-6797-3p	1.59933	0.1	0.1	0.1
hsa-miR-6797-5p	0.1	0.1	0.1	0.1
hsa-miR-6798-3p	0.1	0.1	0.1	0.1
hsa-miR-6798-5p	0.1	0.1	0.1	0.1
hsa-miR-6799-3p	0.1	0.1	0.1	0.1
hsa-miR-6799-5p	0.1	0.1	0.1	0.1
hsa-miR-6800-3p	0.1	0.1	0.1	0.1
hsa-miR-6800-5p	23.9736	29.0327	25.2528	23.5071
hsa-miR-6801-3p	0.1	0.1	0.1	0.1
hsa-miR-6801-5p	0.1	0.1	0.1	0.1
hsa-miR-6802-3p	0.1	0.1	0.1	0.1
hsa-miR-6802-5p	0.1	0.1	0.1	0.1
hsa-miR-6803-3p	0.1	0.1	0.1	0.1

hsa-miR-6803-5p	30.1002	31.906	36.7972	27.1703
hsa-miR-6804-3p	0.1	0.1	0.1	0.1
hsa-miR-6804-5p	0.1	0.1	0.1	0.1
hsa-miR-6805-5p	0.1	0.1	0.1	0.1
hsa-miR-6806-3p	0.1	0.1	0.1	0.1
hsa-miR-6806-5p	0.1	0.1	0.1	0.1
hsa-miR-6807-3p	0.1	0.1	0.1	0.1
hsa-miR-6807-5p	0.1	0.1	0.1	0.1
hsa-miR-6808-3p	0.1	0.1	0.1	0.1
hsa-miR-6808-5p	0.1	0.1	0.1	0.1
hsa-miR-6809-3p	0.1	0.1	0.1	0.1
hsa-miR-6809-5p	0.1	0.1	0.1	0.1
hsa-miR-6810-3p	0.1	0.1	0.1	0.1
hsa-miR-6810-5p	0.1	0.1	0.1	0.1
hsa-miR-6811-3p	0.1	0.1	0.1	0.1
hsa-miR-6811-5p	0.1	0.1	0.1	0.1
hsa-miR-6812-3p	0.1	0.1	0.1	0.1
hsa-miR-6812-5p	2.05634	2.26791	0.1	0.1
hsa-miR-6813-3p	0.1	0.1	0.1	0.1
hsa-miR-6813-5p	0.1	0.1	0.1	0.1
hsa-miR-6814-3p	0.1	0.1	0.1	0.1
hsa-miR-6814-5p	0.1	0.1	0.1	0.1
hsa-miR-6815-3p	0.1	0.1	0.1	0.1
hsa-miR-6815-5p	0.1	0.1	0.1	0.1
hsa-miR-6816-3p	0.1	0.1	0.1	0.1
hsa-miR-6816-5p	0.1	0.1	0.1	0.1
hsa-miR-6817-3p	0.1	0.1	0.1	0.1
hsa-miR-6817-5p	0.1	0.1	0.1	0.1
hsa-miR-6818-3p	0.1	0.1	0.1	0.1
hsa-miR-6818-5p	0.1	0.1	0.1	0.1
hsa-miR-6819-3p	0.1	0.1	0.1	0.1
hsa-miR-6819-5p	0.1	0.1	0.1	0.1
hsa-miR-6820-3p	0.1	0.1	0.1	0.1
hsa-miR-6820-5p	0.1	0.1	0.1	0.1
hsa-miR-6821-3p	0.1	0.1	0.1	0.1
hsa-miR-6821-5p	40.9028	38.6151	47.9495	26.1826
hsa-miR-6822-3p	0.1	0.1	0.1	0.1
hsa-miR-6822-5p	0.1	0.1	0.1	0.1
hsa-miR-6823-3p	0.1	0.1	0.1	0.1
hsa-miR-6823-5p	0.1	0.1	0.1	0.1
hsa-miR-6824-3p	0.1	0.1	0.1	0.1
hsa-miR-6824-5p	0.1	0.1	0.1	0.1
hsa-miR-6825-3p	0.1	0.1	0.1	0.1
hsa-miR-6825-5p	0.1	0.1	0.1	0.1
hsa-miR-6826-3p	0.1	0.1	0.1	0.1
hsa-miR-6826-5p	12.9352	36.6505	28.3383	59.1977
hsa-miR-6827-3p	0.1	0.1	0.1	0.1
hsa-miR-6827-5p	0.1	0.1	0.1	0.1
hsa-miR-6828-3p	0.1	0.1	0.1	0.1
hsa-miR-6828-5p	0.1	0.1	0.1	0.1
hsa-miR-6829-3p	0.1	0.1	0.1	0.1
hsa-miR-6829-5p	8.52557	13.6514	35.3926	9.48756
hsa-miR-6830-3p	0.1	0.1	0.1	0.1

hsa-miR-6830-5p	0.1	0.1	0.1	0.1
hsa-miR-6831-3p	0.1	0.1	0.1	0.1
hsa-miR-6831-5p	0.1	0.1	0.1	0.1
hsa-miR-6832-3p	0.1	0.1	0.1	0.1
hsa-miR-6832-5p	0.1	0.1	0.1	0.1
hsa-miR-6833-3p	0.1	0.1	0.1	0.1
hsa-miR-6833-5p	0.1	0.1	0.1	0.1
hsa-miR-6834-3p	0.1	0.1	0.1	0.1
hsa-miR-6834-5p	0.1	0.1	0.1	0.1
hsa-miR-6835-3p	0.1	0.1	0.1	0.1
hsa-miR-6835-5p	0.1	0.1	0.1	0.1
hsa-miR-6836-3p	0.1	0.1	0.1	0.1
hsa-miR-6836-5p	0.1	0.1	0.1	0.1
hsa-miR-6837-3p	0.1	0.1	0.1	0.1
hsa-miR-6837-5p	0.1	0.1	0.1	0.1
hsa-miR-6838-3p	0.1	0.1	0.1	0.1
hsa-miR-6838-5p	0.1	0.1	0.1	0.1
hsa-miR-6839-3p	0.1	0.1	0.1	0.1
hsa-miR-6839-5p	0.1	0.1	0.1	0.1
hsa-miR-6840-3p	0.1	0.1	0.1	0.1
hsa-miR-6840-5p	0.1	0.1	0.1	0.1
hsa-miR-6841-3p	0.1	0.1	0.1	0.1
hsa-miR-6841-5p	0.1	0.1	0.1	0.1
hsa-miR-6842-3p	0.1	0.1	0.1	0.1
hsa-miR-6842-5p	0.1	0.1	0.1	0.1
hsa-miR-6843-3p	0.1	0.1	0.1	0.1
hsa-miR-6844	0.1	0.1	0.1	0.1
hsa-miR-6845-3p	0.1	0.1	0.1	0.1
hsa-miR-6845-5p	0.1	0.1	0.1	0.1
hsa-miR-6846-3p	0.1	0.1	0.1	0.1
hsa-miR-6846-5p	0.1	0.1	0.1	0.1
hsa-miR-6847-3p	0.1	0.1	0.1	0.1
hsa-miR-6847-5p	0.1	0.1	0.1	0.1
hsa-miR-6848-3p	0.1	0.1	0.1	0.1
hsa-miR-6848-5p	0.1	0.1	0.1	0.1
hsa-miR-6849-3p	0.1	0.1	0.1	0.1
hsa-miR-6849-5p	0.1	0.1	0.1	0.1
hsa-miR-6850-3p	0.1	0.1	0.1	0.1
hsa-miR-6850-5p	5.78274	4.54414	5.57743	1.62713
hsa-miR-6851-3p	0.1	0.1	0.1	0.1
hsa-miR-6851-5p	0.1	0.1	0.1	0.1
hsa-miR-6852-3p	0.1	0.1	0.1	0.1
hsa-miR-6852-5p	0.1	0.1	0.1	0.1
hsa-miR-6853-3p	0.1	0.1	0.1	0.1
hsa-miR-6853-5p	0.1	0.1	0.1	0.1
hsa-miR-6854-3p	0.1	0.1	0.1	0.1
hsa-miR-6854-5p	0.1	0.1	0.1	0.1
hsa-miR-6855-3p	0.1	0.1	0.1	0.1
hsa-miR-6855-5p	0.1	0.1	0.1	0.1
hsa-miR-6856-3p	0.1	0.1	0.1	0.1
hsa-miR-6856-5p	0.1	0.1	0.1	0.1
hsa-miR-6857-3p	0.1	0.1	0.1	0.1
hsa-miR-6857-5p	0.1	0.1	0.1	0.1

hsa-miR-6858-3p	0.1	0.1	0.1	0.1
hsa-miR-6858-5p	0.1	0.1	0.1	0.1
hsa-miR-6859-3p	0.1	0.1	0.1	0.1
hsa-miR-6859-5p	0.1	0.1	0.1	0.1
hsa-miR-6860	0.1	0.1	0.1	0.1
hsa-miR-6861-3p	0.1	0.1	0.1	0.1
hsa-miR-6861-5p	0.1	0.1	0.1	0.1
hsa-miR-6862-3p	0.1	0.1	0.1	0.1
hsa-miR-6862-5p	0.1	0.1	0.1	0.1
hsa-miR-6863	0.1	0.1	0.1	0.1
hsa-miR-6864-3p	0.1	0.1	0.1	0.1
hsa-miR-6864-5p	0.1	0.1	0.1	0.1
hsa-miR-6865-3p	0.1	0.1	0.1	0.1
hsa-miR-6865-5p	0.1	0.1	0.1	0.1
hsa-miR-6866-3p	0.1	0.1	0.1	0.1
hsa-miR-6866-5p	0.1	0.1	0.1	0.1
hsa-miR-6867-3p	0.1	0.1	0.1	0.1
hsa-miR-6867-5p	0.1	0.1	0.1	0.1
hsa-miR-6868-3p	0.1	0.1	0.1	0.1
hsa-miR-6868-5p	0.1	0.1	0.1	0.1
hsa-miR-6869-3p	0.1	0.1	0.1	0.1
hsa-miR-6869-5p	72.9881	74.5533	89.2659	58.0817
hsa-miR-6870-3p	0.1	0.1	0.1	0.1
hsa-miR-6870-5p	0.1	0.1	0.1	0.1
hsa-miR-6871-3p	0.1	0.1	0.1	0.1
hsa-miR-6871-5p	0.1	0.1	0.1	0.1
hsa-miR-6872-3p	0.1	0.1	0.1	0.1
hsa-miR-6872-5p	0.1	0.1	0.1	0.1
hsa-miR-6873-3p	0.1	0.1	0.1	0.1
hsa-miR-6873-5p	0.1	0.1	0.1	0.1
hsa-miR-6874-3p	0.1	0.1	0.1	0.1
hsa-miR-6874-5p	0.1	0.1	0.1	0.1
hsa-miR-6875-3p	0.1	0.1	0.1	0.1
hsa-miR-6875-5p	126.202	129.842	117.249	145.3
hsa-miR-6876-3p	0.1	0.1	0.1	0.1
hsa-miR-6876-5p	0.1	0.1	0.1	0.1
hsa-miR-6877-3p	0.1	0.1	0.1	0.1
hsa-miR-6877-5p	0.1	0.1	0.1	0.1
hsa-miR-6878-3p	0.1	0.1	0.1	0.1
hsa-miR-6878-5p	0.1	0.1	0.1	0.1
hsa-miR-6879-3p	0.1	0.1	0.1	0.1
hsa-miR-6879-5p	26.9375	27.3443	42.6799	27.2687
hsa-miR-6880-3p	0.1	0.1	0.1	0.1
hsa-miR-6880-5p	0.1	0.1	12.2604	0.1
hsa-miR-6881-3p	0.1	0.1	0.1	0.1
hsa-miR-6881-5p	0.1	0.1	0.1	0.1
hsa-miR-6882-3p	0.1	0.1	0.1	0.1
hsa-miR-6882-5p	0.1	0.1	0.1	0.1
hsa-miR-6883-3p	0.1	0.1	0.1	0.1
hsa-miR-6883-5p	0.1	0.1	0.1	0.1
hsa-miR-6884-3p	0.1	0.1	0.1	0.1
hsa-miR-6884-5p	0.1	0.1	0.1	0.1
hsa-miR-6885-3p	0.1	0.1	0.1	0.1

hsa-miR-6885-5p	0.1	0.1	0.1	0.1
hsa-miR-6886-3p	0.1	0.1	0.1	0.1
hsa-miR-6886-5p	0.1	0.1	0.1	0.1
hsa-miR-6887-3p	0.1	0.1	0.1	0.1
hsa-miR-6887-5p	0.1	0.1	0.1	0.1
hsa-miR-6888-3p	0.1	0.1	0.1	0.1
hsa-miR-6888-5p	0.1	0.1	0.1	0.1
hsa-miR-6889-3p	0.1	0.1	0.1	0.1
hsa-miR-6889-5p	0.1	0.1	0.1	0.1
hsa-miR-6890-3p	0.1	0.1	0.1	0.1
hsa-miR-6890-5p	0.1	0.1	0.1	0.1
hsa-miR-6891-3p	0.1	0.1	0.1	0.1
hsa-miR-6891-5p	10.1252	10.2401	12.3319	6.25901
hsa-miR-6892-3p	0.1	0.1	0.1	0.1
hsa-miR-6892-5p	0.1	0.1	0.1	0.1
hsa-miR-6893-3p	0.1	0.1	0.1	0.1
hsa-miR-6893-5p	10.6207	11.8448	11.2891	8.93935
hsa-miR-6894-3p	0.1	0.1	0.1	0.1
hsa-miR-6894-5p	0.1	0.1	0.1	0.1
hsa-miR-6895-3p	0.1	0.1	0.1	0.1
hsa-miR-6895-5p	0.1	0.1	0.1	0.1
hsa-miR-708-3p	0.1	0.1	0.1	0.1
hsa-miR-708-5p	0.1	0.1	0.1	0.1
hsa-miR-7106-3p	0.1	0.1	0.1	0.1
hsa-miR-7106-5p	0.1	0.1	0.1	0.1
hsa-miR-7107-3p	0.1	0.1	0.1	0.1
hsa-miR-7107-5p	11.0767	11.6197	17.8405	7.33056
hsa-miR-7108-3p	0.1	0.1	0.1	0.1
hsa-miR-7108-5p	0.1	0.1	2.78621	0.1
hsa-miR-7109-3p	0.1	0.1	0.1	0.1
hsa-miR-7109-5p	0.1	0.1	0.1	0.1
hsa-miR-711	0.1	0.1	0.1	0.1
hsa-miR-7110-3p	0.1	0.1	0.1	0.1
hsa-miR-7110-5p	13.3541	14.7216	14.5021	9.2837
hsa-miR-7111-3p	0.1	0.1	0.1	0.1
hsa-miR-7111-5p	0.1	0.1	0.1	0.1
hsa-miR-7112-3p	0.1	0.1	0.1	0.1
hsa-miR-7112-5p	0.1	0.1	0.1	0.1
hsa-miR-7113-3p	0.1	0.1	0.1	0.1
hsa-miR-7113-5p	0.1	0.1	0.1	0.1
hsa-miR-7114-3p	0.1	0.1	0.1	0.1
hsa-miR-7114-5p	0.1	2.01157	0.1	5.87788
hsa-miR-7-1-3p	0.1	0.1	0.1	0.1
hsa-miR-7150	8.56559	14.8266	16.8793	9.63764
hsa-miR-7151-3p	0.1	0.1	0.1	0.1
hsa-miR-7151-5p	0.1	0.1	0.1	0.1
hsa-miR-7152-3p	4.57345	2.04044	12.6767	0.1
hsa-miR-7152-5p	0.1	0.1	0.1	0.1
hsa-miR-7153-3p	0.1	0.1	0.1	0.1
hsa-miR-7153-5p	0.1	0.1	0.1	0.1
hsa-miR-7154-3p	0.1	0.1	0.1	0.1
hsa-miR-7154-5p	0.1	0.1	0.1	0.1
hsa-miR-7155-3p	0.1	0.1	0.1	0.1

hsa-miR-7155-5p	0.1	0.1	0.1	0.1
hsa-miR-7156-3p	0.1	0.1	0.1	0.1
hsa-miR-7156-5p	0.1	0.1	0.1	0.1
hsa-miR-7157-3p	0.1	0.1	0.1	0.1
hsa-miR-7157-5p	0.1	0.1	0.1	0.1
hsa-miR-7158-3p	0.1	0.1	0.1	0.1
hsa-miR-7158-5p	0.1	0.1	0.1	0.1
hsa-miR-7159-3p	0.1	0.1	0.1	0.1
hsa-miR-7159-5p	0.1	0.1	11.0541	0.1
hsa-miR-7160-3p	0.1	0.1	0.1	0.1
hsa-miR-7160-5p	0.1	0.1	0.1	0.1
hsa-miR-7161-3p	0.1	0.1	0.1	0.1
hsa-miR-7161-5p	0.1	0.1	0.1	0.1
hsa-miR-7162-3p	0.1	0.1	0.1	0.1
hsa-miR-7162-5p	0.1	0.1	0.1	0.1
hsa-miR-718	0.1	0.1	0.1	0.1
hsa-miR-7-2-3p	0.1	0.1	0.1	0.1
hsa-miR-744-3p	0.1	0.1	0.1	0.1
hsa-miR-744-5p	0.1	0.1	0.1	0.1
hsa-miR-7515	0.1	0.1	0.1	0.1
hsa-miR-758-3p	0.1	0.1	0.1	0.1
hsa-miR-758-5p	0.1	0.1	0.1	0.1
hsa-miR-759	0.1	0.1	0.1	0.1
hsa-miR-7-5p	3.96515	4.08054	3.21278	2.94017
hsa-miR-760	0.1	0.1	0.1	0.1
hsa-miR-761	0.1	0.1	0.1	0.1
hsa-miR-762	7.88971	8.59948	5.33484	4.5946
hsa-miR-764	0.1	0.1	0.1	0.1
hsa-miR-7641	289.16	356.901	287.453	351.372
hsa-miR-765	0.1	0.1	0.1	0.1
hsa-miR-766-3p	0.1	0.1	0.1	0.1
hsa-miR-766-5p	0.1	0.1	0.1	0.1
hsa-miR-767-3p	0.1	0.1	0.1	0.1
hsa-miR-767-5p	0.1	0.1	0.1	0.1
hsa-miR-769-3p	0.1	0.1	0.1	0.1
hsa-miR-769-5p	1.97061	1.83066	0.1	2.73252
hsa-miR-7702	0.1	0.1	0.1	0.1
hsa-miR-7703	0.1	0.1	0.1	0.1
hsa-miR-7704	42.5147	43.4375	57.063	37.3586
hsa-miR-7705	0.1	0.1	0.1	0.1
hsa-miR-770-5p	0.1	0.1	0.1	0.1
hsa-miR-7706	0.1	0.1	0.1	0.1
hsa-miR-7843-3p	0.1	0.1	0.1	0.1
hsa-miR-7843-5p	0.1	0.1	0.1	0.1
hsa-miR-7844-5p	0.1	0.1	0.1	0.1
hsa-miR-7845-5p	0.1	0.1	4.67859	0.1
hsa-miR-7846-3p	0.1	0.1	0.1	0.1
hsa-miR-7847-3p	7.36335	6.19066	11.1847	2.83108
hsa-miR-7848-3p	0.1	0.1	0.1	0.1
hsa-miR-7849-3p	0.1	0.1	0.1	0.1
hsa-miR-7850-5p	0.1	0.1	0.1	0.1
hsa-miR-7851-3p	0.1	0.1	0.1	0.1
hsa-miR-7852-3p	0.1	0.1	0.1	0.1

hsa-miR-7853-5p	0.1	0.1	0.1	0.1
hsa-miR-7854-3p	0.1	0.1	0.1	0.1
hsa-miR-7855-5p	0.1	0.1	0.1	0.1
hsa-miR-7856-5p	0.1	0.1	0.1	0.1
hsa-miR-7973	0.1	0.1	0.1	0.1
hsa-miR-7974	0.1	0.1	0.1	0.1
hsa-miR-7975	9607.13	19786.8	12560.9	25987
hsa-miR-7976	0.1	0.1	0.1	0.1
hsa-miR-7977	3808.74	10009.6	3789.2	13856.5
hsa-miR-7978	0.1	0.1	0.1	0.1
hsa-miR-802	0.1	0.1	0.1	0.1
hsa-miR-8052	0.1	0.1	0.1	0.1
hsa-miR-8053	0.1	0.1	0.1	0.1
hsa-miR-8054	0.1	0.1	0.1	0.1
hsa-miR-8055	0.1	0.1	0.1	0.1
hsa-miR-8056	0.1	0.1	0.1	0.1
hsa-miR-8057	0.1	0.1	0.1	0.1
hsa-miR-8058	0.1	0.1	0.1	0.1
hsa-miR-8059	0.1	0.1	0.1	0.1
hsa-miR-8060	0.1	0.1	0.1	0.1
hsa-miR-8061	0.1	0.1	0.1	0.1
hsa-miR-8062	0.1	0.1	0.1	0.1
hsa-miR-8063	8.11988	4.50258	5.65139	2.86285
hsa-miR-8064	0.1	0.1	0.1	0.1
hsa-miR-8065	0.1	0.1	0.1	0.1
hsa-miR-8066	0.1	0.1	0.1	0.1
hsa-miR-8067	0.1	0.1	0.1	0.1
hsa-miR-8068	0.1	0.1	0.1	0.1
hsa-miR-8069	574.025	659.984	1399.36	674.629
hsa-miR-8070	0.1	0.1	0.1	0.1
hsa-miR-8071	0.1	0.1	0.1	0.1
hsa-miR-8072	5.24625	5.71904	7.52741	4.80795
hsa-miR-8073	0.1	0.1	0.1	0.1
hsa-miR-8074	0.1	0.1	0.1	0.1
hsa-miR-8075	0.1	0.1	0.1	0.1
hsa-miR-8076	0.1	0.1	0.1	0.1
hsa-miR-8077	0.1	0.1	0.1	0.1
hsa-miR-8078	0.1	0.1	0.1	0.1
hsa-miR-8079	0.1	0.1	0.1	0.1
hsa-miR-8080	0.1	0.1	0.1	0.1
hsa-miR-8081	0.1	0.1	0.1	0.1
hsa-miR-8082	0.1	0.1	0.1	0.1
hsa-miR-8083	0.1	0.1	0.1	0.1
hsa-miR-8084	0.1	0.1	0.1	0.1
hsa-miR-8085	0.1	0.1	0.1	0.1
hsa-miR-8086	0.1	0.1	0.1	0.1
hsa-miR-8087	0.1	0.1	0.1	0.1
hsa-miR-8088	0.1	0.1	0.1	0.1
hsa-miR-8089	17.1932	30.9121	115.28	24.0372
hsa-miR-8485	1.69204	2.13897	17.66	1.69644
hsa-miR-873-3p	0.1	0.1	0.1	0.1
hsa-miR-873-5p	0.1	0.1	0.1	0.1
hsa-miR-874-3p	1.57864	0.1	2.93486	0.1

hsa-miR-874-5p	0.1	0.1	0.1	0.1
hsa-miR-875-3p	0.1	0.1	0.1	0.1
hsa-miR-875-5p	0.1	0.1	0.1	0.1
hsa-miR-876-3p	0.1	0.1	0.1	0.1
hsa-miR-876-5p	0.1	0.1	0.1	0.1
hsa-miR-877-5p	0.1	0.1	0.1	0.1
hsa-miR-885-3p	0.1	0.1	0.1	0.1
hsa-miR-885-5p	0.1	0.1	0.1	0.1
hsa-miR-887-3p	0.1	0.1	0.1	0.1
hsa-miR-887-5p	0.1	0.1	0.1	0.1
hsa-miR-888-3p	0.1	0.1	0.1	0.1
hsa-miR-888-5p	0.1	0.1	0.1	0.1
hsa-miR-889-3p	0.1	0.1	0.1	0.1
hsa-miR-889-5p	0.1	0.1	0.1	0.1
hsa-miR-890	0.1	0.1	0.1	0.1
hsa-miR-891a-3p	0.1	0.1	0.1	0.1
hsa-miR-891a-5p	0.1	0.1	0.1	0.1
hsa-miR-891b	0.1	0.1	0.1	0.1
hsa-miR-892a	0.1	0.1	0.1	0.1
hsa-miR-892b	0.1	0.1	0.1	0.1
hsa-miR-892c-3p	0.1	0.1	0.1	0.1
hsa-miR-892c-5p	0.1	0.1	0.1	0.1
hsa-miR-920	0.1	0.1	0.1	0.1
hsa-miR-921	0.1	0.1	0.1	0.1
hsa-miR-922	0.1	0.1	0.1	0.1
hsa-miR-924	0.1	0.1	0.1	0.1
hsa-miR-92a-1-5p	0.1	0.1	0.1	0.1
hsa-miR-92a-2-5p	0.1	0.1	0.1	0.1
hsa-miR-92a-3p	52.2052	53.2595	52.7759	40.0829
hsa-miR-92b-3p	0.1	0.1	0.1	0.1
hsa-miR-92b-5p	0.1	0.1	0.1	0.1
hsa-miR-933	0.1	0.1	0.1	0.1
hsa-miR-93-3p	0.1	0.1	0.1	0.1
hsa-miR-934	0.1	0.1	0.1	0.1
hsa-miR-935	0.1	0.1	0.1	0.1
hsa-miR-93-5p	70.6096	67.8591	39.1236	88.3826
hsa-miR-936	0.1	0.1	0.1	0.1
hsa-miR-937-3p	0.1	0.1	0.1	0.1
hsa-miR-937-5p	5.19723	4.37555	0.1	0.1
hsa-miR-938	0.1	0.1	0.1	0.1
hsa-miR-939-3p	0.1	0.1	0.1	0.1
hsa-miR-939-5p	3.05812	5.04215	4.29756	4.92583
hsa-miR-9-3p	0.1	0.1	0.1	0.1
hsa-miR-940	1.82418	1.95268	1.99909	0.1
hsa-miR-941	0.1	0.1	0.1	0.1
hsa-miR-942-3p	0.1	0.1	0.1	0.1
hsa-miR-942-5p	0.1	0.1	0.1	0.1
hsa-miR-943	0.1	0.1	0.1	0.1
hsa-miR-944	0.1	0.1	0.1	0.1
hsa-miR-9500	0.1	0.1	0.1	0.1
hsa-miR-95-3p	0.1	0.1	0.1	0.1
hsa-miR-95-5p	0.1	0.1	0.1	0.1
hsa-miR-9-5p	0.1	0.1	0.1	0.1

hsa-miR-96-3p	0.1	0.1	0.1	0.1
hsa-miR-96-5p	34.4941	47.4132	23.0938	59.1563
hsa-miR-98-3p	0.1	0.1	0.1	0.1
hsa-miR-98-5p	15.3502	13.961	14.0772	17.2799
hsa-miR-99a-3p	0.1	0.1	0.1	0.1
hsa-miR-99a-5p	24.0761	29.4514	19.6286	35.638
hsa-miR-99b-3p	0.1	0.1	0.1	0.1
hsa-miR-99b-5p	32.8368	40.2669	27.009	59.5509
